# Systematic evaluation and optimization of TaqMan qPCR assays targeting F*57*, ISMAP*02*, and IS*900* for multiplex detection of *Mycobacterium avium* subsp. *paratuberculosis*

**DOI:** 10.1128/jcm.00872-25

**Published:** 2025-12-29

**Authors:** Nathalie Bissonnette, Séverine Ollier, Jean-Philippe Brousseau, Alexander Stephan Byrne, Christian Savard, Kapil Tahlan

**Affiliations:** 1 Agriculture and Agri-Food Canada, Sherbrooke Research and Development Centre, Sherbrooke, Quebec, Canada; 2 Department of Pathology and Microbiology, Faculty of Veterinary Medicine, Université de Montréal, Saint-Hyacinthe, Quebec, Canada; 3 Department of Biology, Memorial University of Newfoundland 7512, St. John's, Newfoundland and Labrador, Canada; 4 Biovet, Saint-Hyacinthe, Quebec, Canada; University of California, Davis, Davis, California, USA

**Keywords:** Johne’s disease, paratuberculosis, diagnostic, multiplex qPCR, quantitative PCR

## Abstract

This study aimed to develop a multiplex quantitative PCR (qPCR) assay for the detection of *Mycobacterium avium* subsp. *paratuberculosis* (MAP), the etiological agent of paratuberculosis disease, a chronic and endemic infectious disease affecting ruminant livestock worldwide. Infected animals may remain asymptomatic for years while intermittently shedding MAP into their environment through feces, contributing to ongoing transmission. To develop a robust multiplex qPCR assay, we reviewed all TaqMan qPCR studies published since 1990 and selected 18 primer-probe combinations targeting the MAP-specific F*57* gene and the repetitive sequence elements ISMAP*02* and IS*900*. In samples with moderate to high MAP levels, all combinations performed well, with only minor differences in analytical performance. However, in low-abundance samples, several TaqMan designs yielded unreliable results, indicating limited specificity in complex matrices. Among the evaluated assays, the IS*900*-Herthnek design demonstrated significantly higher diagnostic sensitivity, detecting MAP in 81% of low-abundance samples, compared to 0% and 3% for the IS*900*-Kim and IS*900*-Slana assays, respectively. For ISMAP*02*, only the ISMAP*02*-Sevilla assay produced reliable results. For F*57*, Herthnek provided the most consistent and accurate quantification. Similar trends were observed in environmental sample testing. Based on these findings, we recommend a multiplex qPCR assay incorporating the IS*900*-Herthnek, ISMAP*02*-Sevilla, and F*57*-Herthnek TaqMan designs for the detection of MAP in fecal and environmental samples. This combination offers high analytical sensitivity and specificity, making it a valuable and accurate tool for the diagnosis of paratuberculosis and for environmental surveillance on dairy farms to identify herds potentially harboring MAP-infected animals.

**IMPORTANCE:**

*Mycobacterium avium* subsp. *paratuberculosis* (MAP) is the etiological agent of Johne’s disease (JD) in ruminant livestock industries and has been associated with Crohn’s disease in humans. Emerging scientific evidence also links MAP to other human conditions, including inflammatory bowel disease, autoimmune disorders, colorectal cancer, and Alzheimer’s disease. This potential public health threat has intensified interest in developing more sensitive diagnostic tools and effective control strategies to eradicate MAP from dairy herds. Infected ruminants typically remain in the subclinical stage of the disease for 2–5 years, during which they shed MAP in their feces and contaminate the environment. Diagnosis during this stage is particularly challenging, as the pathogen evades the host’s immune response, rendering serological tests insufficiently sensitive. In contrast, fecal PCR offers greater sensitivity than serum ELISA and traditional culture methods. Multiplex quantitative PCR is especially promising due to its high specificity and sensitivity for detecting MAP-infected animals and identifying herds with active shedders. Herd-level environmental screening, followed by individual animal testing, represents a robust national biosecurity strategy. This approach is a critical step toward reducing MAP transmission and improving herd health within the dairy industry.

## INTRODUCTION

*Mycobacterium avium* subsp. *paratuberculosis* (MAP) is the etiological agent of Johne’s disease (JD), a chronic and debilitating condition affecting ruminant livestock worldwide. MAP has also been associated with Crohn’s disease in humans (1–4), and increasing scientific evidence also links MAP to other human conditions, including other inflammatory bowel diseases, autoimmune disorders, colorectal cancer, and Alzheimer’s disease (5, 6). This potential zoonotic threat has intensified efforts to develop more sensitive diagnostic tools and effective control strategies to eradicate MAP from dairy herds and to safeguard the food supply through enhanced biovigilance across the farm-to-fork continuum.

The diagnosis of paratuberculosis remains challenging due to the limited sensitivity of current diagnostic methods. Historically, fecal culture has been used as an *ante-mortem* diagnostic test. However, MAP is a slow-growing bacterium, requiring several weeks to months for detection. Furthermore, the decontamination steps necessary to suppress contaminating flora are also harsh on MAP, resulting in a 1–2 log (approximately 99%) reduction in viability (7, 8). In subclinical animals, where MAP shedding is low, culture methods often fail to detect infection (7, 9). Serological detection using ELISA is widely employed due to its low cost, high throughput, and rapid turnaround time. However, its sensitivity in cattle is low (~0.44), particularly in early and subclinical stages of infection, resulting in limited diagnostic accuracy (10, 11). Since the 1990s, the development of molecular methods targeting specific bacterial DNA sequences has enabled the rapid identification of fastidious organisms such as MAP. Among these, direct fecal PCR has shown the highest correlation with fecal culture compared to blood- or milk-based diagnostics (12, 13), and it yields fewer false negatives in subclinical animals (9, 14, 15). Quantitative PCR (qPCR) methods have gained popularity due to their speed, sensitivity, and ability to quantify bacterial load. However, commercial qPCR kits remain costly and often lack transparency, as the primer and probe sequences are typically proprietary.

This study aimed to develop and validate a multiplex qPCR assay for the detection of MAP, in accordance with the Minimum Information for Publication of Quantitative real-time PCR experiments (MIQE) guidelines (16). Multiplex PCR is a powerful tool for achieving robust and meaningful diagnostic results. The F*57* gene is unique to MAP and is absent in other members of the *Mycobacterium avium* complex (MAC), making it a highly specific target for detecting MAP (17, 18). As F*57* is present in only a single copy per genome, combining it with multicopy targets such as IS*900* (17 copies) (19) and ISMAP*02* (six copies) (20) enhances assay sensitivity.

We conducted a systematic review of published TaqMan qPCR assays since 1990 and identified 18 primer-probe multiplex combinations targeting IS*900*, ISMAP*02*, and F*57*. A comprehensive *in silico* analysis was performed to assess the specificity of each qPCR design. The selected assays were assessed for analytical sensitivity and then validated for diagnostic sensitivity using fecal samples from MAP-infected cows and environmental samples from herds with a history of confirmed clinical paratuberculosis. Including multiple targets in a single reaction not only reduces reagent costs and sample volume requirements but also increases diagnostic confidence by enabling the simultaneous detection of highly sensitive MAP-specific markers. This multiplexing strategy is particularly advantageous for large-scale surveillance and routine diagnostics in veterinary settings.

## MATERIALS AND METHODS

### Systematic review of published F*57* and ISMAP*02* assays

In a previous study, we performed a systematic review to identify the best-performing IS*900* TaqMan assays (21). We used the same strategy to identify qPCR designs for F*57* and ISMAP*02*. To identify publications reporting the use of F*57* and ISMAP*02* target sequences for the detection of MAP by qPCR, the Scopus, Agricola, Biological Abstracts (Ovid), CAB Abstracts (Ovid), FSTA (Ovid), and Medline (Ovid) databases were searched using a previously developed protocol (21). Search results published between 1990 and 8 May 2024 along with the keywords, index terms, and strings used are reported in Table S1A and B for F*57* and ISMAP*02*, respectively. No distinction was made between qPCR assays designed to test milk, tissue, or fecal samples. Articles were excluded if they were (i) opinion articles, (ii) conference abstracts, (iii) theses, or (iv) not written in English or French. The librarians conducted the initial screening of the literature on MAP for the identification of F*57* or ISMAP*02* PCR-related reports, which were imported into EndNote 20 (Clarivate) for further analysis. Information specialists followed Cochrane guidelines for conducting the systematic review, using metadata (in RIS format) to remove duplicates (via EndNote’s “Find Duplicates” function) and organize publications by type. It is important to note that some databases contained metadata errors, such as inverted author names or incorrect publication types, which were identified and corrected during manual review.

### Primer and probe sequences

The annotated F*57* gene, located at positions 886,451–887,722 in the MAP K-10 genome (GenBank accession no. NC_002944.2), was used to align qPCR assay designs targeting this unique sequence. For ISMAP*02* and IS*900*, which are present in 6 and 17 copies, respectively, consensus sequences were generated to facilitate visualization of primer and probe alignments across multiple loci. For ISMAP*02*, the consensus sequence was constructed using the six annotated genomic regions from the MAP K-10 genome (NC_002944.2) at the following positions: 363,072–361,399 (C1), 2,715,954–2,714,281 (C2), 2,809,770–2,811,443 (C3), 2,885,070–2,886,743 (C4), 3,729,378–3,727,705 (C5), and 3,853,724–3,852,051 (C6). The consensus sequence for IS*900* was obtained from GenBank (accession no. PP417235). All TaqMan qPCR primer and probe sequences were mapped to their respective targets using the sequence visualization tool ApE v3.1.3 (22). To evaluate multiplexing efficiency, a total of 18 qPCR assay combinations were created using three designs each for IS*900* and F*57*, and both published designs for ISMAP*02* (3 × 3 × 2). All selected primers and probes used in these multiplex assays are listed in Table 1. As an internal amplification control (IAC) to monitor both DNA extraction and qPCR success, we designed an assay using the Integrated DNA Technologies (IDT) PrimerQuest tool (https://www.idtdna.com/PrimerQuest/Home/Index) targeting the reference β-actin gene of *Bos taurus* (*ACTB*; NCBI Gene ID 280979) using the following forward and reverse primers: 5′-ATCCTGACCCTCAAGTACCC-3′ and 5′-ACACGGAGCTCGTTGTAGA-3′, respectively, and 5′-CACCAACTGGGACGACATGGAGAA-3′ as a probe.

**TABLE 1:** Characteristics of the primers and probe used in 18 multiplex assays

Reference^*a*^	Oligo name^*b*^	Sequence 5′ – 3′	Position^*c*^	Oligo length (nt)^*d*^	Sp^*e*^	Size (bp)^*f*^
IS*900*						
Herthnek et al. (23)	MPF	CCGCTAATTGAGAGATGCGATT	139–160	22	Low	
	MPR	CCAGACAGGTTGTGCCACAA	253–234	20	–	115
	MPP	ACCTCCGTAACCGTCATTGTCCAGATCA	231–204	28	–	
Slana et al. (24)	IS900qPCRF	GATGGCCGAAGGAGATTG	94–111	18	Low	
	IS900qPCRR	CACAACCACCTCCGTAACC	238–220	19	–	145
	IS900qPCRTM	ATTGGATCGCTGTGTAAGGACACGT	158–182	25	–	
Kim et al. (25)	F2	AATGACGGTTACGGAGGTGGT	214–234	21	–	
	R2	GCAGTAATGGTCGGCCTTACC	289–269	21	–	76
	P2	TCCACGCCCGCCCAGACAGG	264–245	20	–	
ISMAP*02*						
Irenge et al. (26)	ISMAP02-for	CGCCAGGAACGCAAACAT	126–109	18	Low	
	ISMAP02-rev	GTGCAGGGTCGCTCTGATG	31–49	19	Low	96
	ISMAP02-probe	ACTCCGCATCCAACAACTCACGCTG	76–52	25	Low	
Sevilla et al. (27)	ISMap02-F	CGGCTGGACACGGAATG	1,209–1,225	17	Low	
	ISMap02-R	CATGAGCGACAGTATCTTTCGAA	1,275–1,253	23	Low	67
	ISMap02-probe	ATCCGTCCCAGTGGCGGAGTCAC	1,229–1,251	23	Low	
F*57*						
Herthnek and Bolske (28)	DH3F	AACTAAGCGGATCGACAATTC	397–377	21	–	
	DH3R	TGGTGTACCGAATGTTGTTG	318–337	20	–	80
	DH3 probe	TGCAACTCGAACACACCTGGGA	371–350	22	–	
Irenge et al. (26)	F57-for	TTCATCGATACCCAAACTCAGAGA	462–439	24	–	
	F57-rev	GTTCGCCGCTTGAATGGT	395–412	18	–	68
	F57-probe	TGCCAGCCGCCCACTCGTG	419–437	19	–	
Ricchi et al. (29)	Forward	ATAGCTTTCCTCTCCTTCGTC	875–855	21	–	
	Reverse	CAGGGCAACAACATATTCGG	736–755	20	–	140
	Probe	TCCAGGAACGCTTGGCACTCG	839–819	21	–	

^*a*^ qPCR designs targeting the respective *Mycobacterium avium* subsp. *paratuberculosis* target sequence, namely IS*900*, ISMAP*02*, and F*57*.

^*b*^ The names of the primers and the probe respect those reported in the publication. In the absence of a name, the usual designation forward, reverse, and probe is given.

^*c*^ Position of primers and probes targeting IS*900* according to the GenBank accession no. PP417235 sequence, targeting ISMAP*02* (consensus sequence of 1,674 nt, Fig. 1), and targeting F*57* according to the GenBank accession no. PP971135 sequence.

^*d*^ Length of primers and probes; nt, number of nucleotides.

^*e*^ Specificity of primer and probe sequences for MAP was evaluated using BLASTn (from NCBI) by filtering out the *Mycobacterium avium* subsp. *paratuberculosis* organism (taxid 1770); BLAST results are found in Table S3A through C, for F57, ISMAP*02*, and IS900, respectively; Sp, specificity; “–” indicates the absence of lack of specificity.

^*f*^ Size of the amplicon generated using the forward and reverse primers.

### Evaluation of analytical specificity

The analytical specificity of ISMAP*02* and F*57* primer and probe sequences for MAP was evaluated *in silico* using the NCBI BLASTn tool. To ensure specificity, searches excluded MAP (TaxID: 1770) from the database. BLASTn results for F*57*, ISMAP*02*, and IS*900* were compiled into an Excel file for further analysis. Hits with 100% query coverage and 100% identity were summarized in the first tab of the file. Potential secondary structures of primers and probes, which can impact PCR efficiency through self-folding or dimerization, were assessed using the OligoAnalyzer tool from IDT. The thermodynamic stability of hairpins and homodimers was evaluated based on Gibbs free energy (ΔG), with thresholds applied to identify sequences with minimal secondary structure interference. All TaqMan qPCR designs were further evaluated *in silico* using whole-genome sequences from MAP field isolates obtained from axenic cultures, as previously described (30). The genomic sequences of an additional 241 MAP field strains that we sequenced for this project (GenBank BioProject PRJNA925907) were deposited in the NCBI Sequence Read Archive (SRA) repository (accession numbers: SRA37985472–SRA37985606). Along with the previously deposited sequences (30, 31), this led to a total of 433 MAP field strains that were used to confirm the number of gene copies and the sequence identity of primers and probes relative to their target regions. In addition, *in silico* analyses of the other MAP strain types (Bison-type, a subcategory of C strain/Type II lineage strains, and the Telford S-type, subtype I identified in sheep or goats) were performed using the BLAST2seq tool and the respective reference genomes (CP033688.1 for Telford S-type and CP033911.1 for MAPK B-type).

### qPCR conditions

The SensiFAST Probe no-ROX kit (Meridian Bioscience) was selected to enable the use of the fourth detection channel for monitoring DNA extraction and qPCR efficiency via an endogenous and internal control. For each assay, the qPCR mixture contained 12.5 µL of master mix, 400 nM of each primer (IDT), 100 nM of the IS*900*, ISMAP*02*, F*57*, and *ACTB* probes (Thermo Fisher Scientific), and 8 µL of DNA, for a final volume of 25 µL, in accordance with the manufacturer’s recommendations. Each probe was labeled with a distinct fluorophore at the 5′ end and a QSY quencher at the 3′ end. The custom TaqMan QSY quencher is compatible with FAM, VIC, ABY, and JUN dyes and is suitable for use with the 7500 Fast Real-Time PCR thermocycler (Applied Biosystems). To ensure clear spectral separation in the multiplex design, each of the four gene-specific probes was assigned a unique dye. The FAM dye, known for its high fluorescence intensity, was used for the single-copy gene F*57*. VIC and ABY were assigned to ISMAP*02* and IS*900*, respectively, while JUN was used for the endogenous IAC, *ACTB*, which also served as an extraction control.

Biological samples were analyzed as duplicates, and each 96-well plate used for qPCR included both negative controls (no template) and positive controls containing MAP K10 genomic DNA (gDNA). The 18 selected multiplex qPCR combinations were compared using a standardized thermal cycling program on a single calibrated thermocycler. The program consisted of an initial denaturation and polymerase activation cycle at 95°C for 10 min, followed by 45 cycles of denaturation at 95°C for 15 s and annealing/extension at 60°C for 1 min, as recommended by the manufacturer. Amplification plots from all runs were visually inspected to confirm the presence and quality of amplification signals.

As a qPCR control, we used 100 genome equivalents (Ge) of DNA from the MAP K-10 reference strain (ATCC BAA-968) in all plates to monitor inter-run variability. The Ge concentration was calculated based on the molecular weight of MAP chromosomal DNA. The MAP K-10 genome is 4,829,781 bp in size (32). DNA from two *Mycobacterium avium* subsp. *hominissuis* (MAH) reference strains: ATCC 700898 and MAH 101 were included for specificity testing, which were kindly provided by Dr. Marcel Behr (McGill University, Canada). For comparison, the genome size of MAH, based on the MAV101 assembly, is approximately 5.5 Mb, with an estimated molecular weight of 3,575,000,000 g/mol. The number of genome copies per reaction was calculated by multiplying the DNA mass in ng by Avogadro’s number and dividing by the molecular weight in ng/mol (33).

### Evaluation of analytical sensitivity

According to MIQE guidelines, it is essential to assess both the amplification efficiency (%Eff) and the limit of detection (LOD) of qPCR assays. For multiplex assays, these parameters must be determined for each target (16). The performance of each primer-probe design was evaluated across the 18 multiplex qPCR assays. The LOD was determined using serial dilutions corresponding to 0.1, 0.2, 0.5, 1, 10, 20, 100, 200, 1,000, 2,000, and 10,000 Ge. Given that stochastic amplification of low-copy DNA templates follows a Poisson distribution, a higher number of replicates (*n* = 7) was used for the lowest DNA concentrations (0.1, 0.2, 0.5, and 1 Ge) to improve statistical confidence. For 10 Ge, three replicates were included, and for concentrations ≥20 Ge, two replicates were used. An arbitrary quantification cycle (Cq) threshold of 38 was selected to define the LOD, in accordance with commonly accepted guidelines (34).

The %Eff of each primer-probe design was calculated for the 18 multiplex assays. The %Eff was calculated from the slope of the standard curve using the formula: Efficiency = 10 (−1/slope), and is expressed as percent efficiency: %Eff = (E − 1) × 100%. In practice, acceptable amplification efficiencies range from 90% to 110%, corresponding to slope values between −3.2 and −3.5 (35). For each multiplex qPCR design, %Eff was calculated from the slope of standard curves generated across three independent runs (i.e., three separate 96-well plate assays) using serial dilutions of MAP K10 DNA.

### Animals and environmental samples

Herds selected for the study were part of a longitudinal investigation involving 22 dairy herds, recruited based on veterinary confirmation that at least one cow with JD had been detected on the farm within the previous year. All animals (>24 months) were sampled (blood and feces) twice a year (every 5–7 months) during a 3-year period by technicians or animal handlers under the supervision of the herd veterinarian and research scientist from Agriculture and Agri-Food Canada (AAFC), as previously described (36). Information about fecal consistency, presence of diarrhea, body condition score, and overall health status was collected on the farm by AAFC at each herd visit. Individual cow feces were collected directly from the rectum using a single-use veterinary glove. Concurrently, individual blood samples were collected in dry tubes (BD Biosciences) for serum separation. In total, 3,458 cows were sampled, yielding 12,087 blood and fecal samples for analysis.

All fecal and blood samples were analyzed using the USDA-licensed VetMAX-Gold MAP Detection Kit (VetMAX; Thermo Fisher Scientific) for fecal qPCR and the IDEXX MAP Ab Test Kit (IDEXX Laboratories) for serum ELISA, as previously described (9, 30, 36). Because the sensitivity and specificity of tests for the diagnosis of paratuberculosis vary significantly depending on the infection stage, it is recommended to include a consistent classification of the animal instead of using a case definition (10, 37). Therefore, we used the longitudinal profile of MAP-fecal excretion and serum ELISA results to classify cows (Fig. S1). We previously determined by culture that MAP-fecal excretion levels can be correlated with VetMAX fecal qPCR results, corresponding to low (Cq > 35, <10 cfu per tube), moderate (Cq ~ 32, 10–100 cfu per tube), and high (Cq ~ 20, too many to count) levels (9). A comparison of MAP shedding levels was performed between qPCR and culture using the same sample, which was processed according to protocols specifically adapted for either molecular (9) or culture (30) analysis, as previously described. Based on this classification, 21 cows were selected: 6 with low (F05–F10; Fig. S1A), 6 with moderate (F11–F16; Fig. S1B), and 9 with high MAP burden (F17–F25; Fig. S1D). Additionally, four cows negative by both fecal qPCR and serum ELISA were included as controls (F01–F04). Each cow’s fecal sample was used to compare all multiplex qPCR assay combinations.

Environmental samples from 22 herds were collected using a standardized description sheet and procedure guided by schematic farm maps for documentation. Briefly, 3–5 environmental samples at each visit were collected from manure storage areas (manure pit or pre-pit) and from lactation alleys (tie-stall) or scraping lines (free-stall). A total of 538 environmental samples were analyzed by qPCR using VetMAX. For the comparison of the 18 multiplex-qPCR combinations, two environmental samples each from four herds (*n* = 8) were used (farms A–D; Fig. S1D). For additional validation of problematic TaqMan designs, environmental samples (pool of 3–5 samples per herd) were prepared from each of the remaining 14 herds, as previously described (38).

To confirm that the multiplex format maintains the analytical sensitivity of each target, we evaluated the diagnostic performance of the final multiplex qPCR assay and compared it to the results obtained from individual singleplex qPCR assays targeting F*57*, ISMAP*02*, and IS*900*. Four samples were tested, with five replicates each, in both multiplex and singleplex conditions, to assess repeatability and concordance between the two techniques. The final version of the multiplex qPCR was validated using 2,100 cows and 463 environmental samples. All reactions were run in parallel on the same 7500 Fast Real-Time PCR thermocycler to ensure consistent amplification conditions. DNA from both fecal and environmental samples was extracted using the Quick-DNA Fecal/Soil Microbe Miniprep Kit (Zymo Research Corp.). The use of a bead beater and zirconium beads in the lysis buffer ensured MAP cell wall disruption. The protocol for DNA extraction, which had been optimized in a previous study (9), included the following steps: two 1-min bead beating periods with a 5-min break interval to prevent the sample from overheating. The purified DNA was finally recovered in 100 µL of elution buffer.

### Statistical analysis

Data were analyzed through a one-way analysis of variance (Statistical Analysis System, Release 9.4, 2002–2012, SAS Institute Inc.) with the 18 selected multiplex qPCR combinations treated as a fixed effect in the model. The 17 degrees of freedom were further partitioned according to a 3 × 2 × 3 factorial design to evaluate the effect of each of the three factors (IS*900* [three designs], ISMAP*02* [two designs], and F*57* [three designs]) and their interactions. For each gene target and within each MAP excretion level (low, medium, or high MAP abundance, or the environmental samples), the outcome variable was the adjusted mean value of Cq for every combination (3 × 2 × 3 for 18 combinations) with their estimated SE. Mean differences between designs (levels of the factors) corresponding to the gene target were evaluated using multiple comparisons with a Tukey adjustment (for the two factors with three levels). Results were considered statistically significant if *P* < 0.05. For the environmental samples and cows with low-MAP abundance excretion, detection of a signal (yes/no) was evaluated through a Chi-square association test. In all cases, a difference was considered significant if the *P* value for the Chi-square test was <0.05. The concordance correlation coefficient (r_c_) was calculated to assess agreement on a repetitive measure obtained with singleplex and multiplex methods, or to evaluating alternative annealing temperatures of the pPCR program.

## RESULTS

### Selection of qPCR designs for F*57*, ISMAP*02,* and IS*900*

The three primary gene targets used for MAP detection, IS*900*, ISMAP*02,* and F*57,* were selected to develop a multiplex qPCR. We applied the same strategy previously used for IS*900* TaqMan assays (21) to identify suitable designs for ISMAP*02* and F*57*. The initial literature search yielded 19,154 records. After removing duplicate publications and excluding articles that did not involve PCR, as well as conference abstracts, reviews, and publications in languages other than English or French, 86 articles on F*57* and 23 articles on ISMAP*02* were retained. Further exclusion of studies that employed SYBR Green chemistry, commercial assays, or PCR assays designed for nested-PCR, loop-mediated-PCR, or standard PCR (e.g., agarose gel electrophoresis) led to the retention of 39 publications that used TaqMan assays for detecting MAP based on the F*57* gene and four publications used ISMAP*02* (Table S2A and B, respectively). Following additional screening to remove redundant citations using the same primer/probe sets, we identified seven unique TaqMan assay designs targeting F*57* and two targeting ISMAP*02* (Table S2A and B, respectively).

### *In silico* analysis of the ISMAP*02* designs

**Fig 1:**
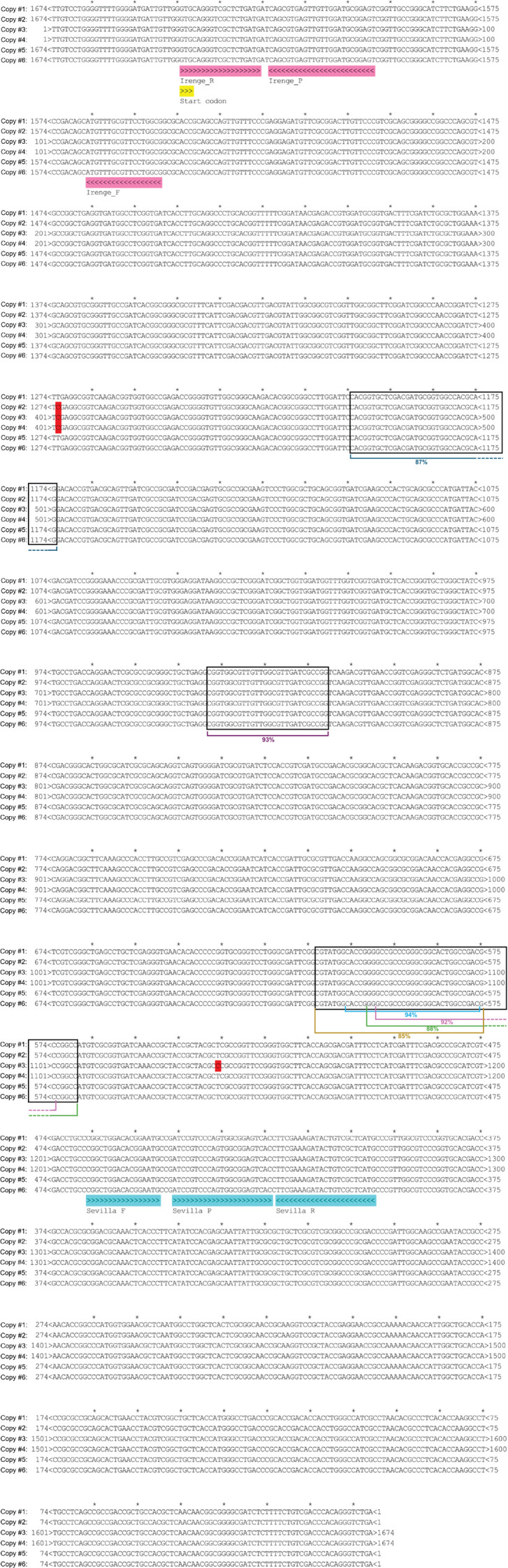
Alignment of the six copies of ISMAP*02* and location of primers and probe from each TaqMan assay design. The C1–C6 sequences were extracted from GenBank accession no. NC_002944.2, at genomic positions 363,072–361,399, 2,715,954–2,714,281, 2,809,770–2,811,443, 2,885,070–2,886,743, 3,729,378–3,727,705, and 3,853,724–3,852,051, respectively. Differences between copies are highlighted in red. Two mismatches were identified in ISMAP*02*, notably in position 402 for copies nos. C2, C3, and C4, and one additional genetic variation was detected in position 1,139 of C3. Primer (F for forward and R for reverse, as described in the original publication) and probe (P) annotations include directionality (>>> for sense and <<< for reverse strand). The GTG translation initiation codon (yellow box) of ISMAP*02* is at positions 31–33. Regions showing similarity (blastn) to MAH strains 101 and 104 are boxed in black, and percentage of similarity is reported.

We mapped the TaqMan designs to the respective MAP gene sequence. For ISMAP*02*, we extracted the six repetitive sequences (C1–C6) from the MAP K-10 reference genome (GenBank accession No. NC_002944.2). Although the six ISMAP*02* copies are similar, two DNA mismatches were identified, one at position 402 in C2, C3, and C4, and one genetic variation was detected at position 1,139 in C3 (Fig. 1). The two ISMAP*02*-Irenge (26) and ISMAP*02*-Sevilla designs (27) do not overlap these genetic variations (Fig. 1). Furthermore, no genetic variations were identified in the six ISMAP*02* genomic copies in the 433 MAP strains we sequenced (30, 31), including 129 that were analyzed most recently (GenBank BioProject acc. No. PRJNA925907).

*In silico* BLAST analysis showed that while primers and probes of the two ISMAP*02* TaqMan designs are specific to MAP, matches were found in 14 deposited genomes of MAH (including GenBank accessions AP020326.1, CP040247.1, CP040250.1, CP035744.1, CP018019.2, CP018363.1, AP012555.1, CP036220.2, CP040255.1, CP060405.1, CP018014.3, CP018020.3, CP016818.3, and CP083998.1) (Table S3A). A further screen of 190 MAH genomes, including 70 well-characterized isolates (39), had no similarity between the ISMAP*02* gene and these MAH strains, except for the 14 deposited genomes reported above. Moreover, the limited similarity observed between ISMAP*02* gene and MAH strains 101 or 104 was restricted to three short regions that did not overlap the primer-probe TaqMan binding regions (Fig. 1, black boxes). To further evaluate the analytical specificity of the ISMAP*02* designs, we performed qPCR using DNA from two reference strains, MAH ATCC 700898 and MAH 101, using both ISMAP*02-*Irenge and ISMAP*02-*Sevilla designs, as well as the commercial USDA-licensed VetMAX qPCR assay. There was no detection of MAH with up to 1,000 Ge of MAH ATCC 700898 or MAH 101 DNA using both in-house ISMAP*02* TaqMan designs and the VetMAX qPCR assay.

### *In silico* analysis of the F*57* designs

**Fig 2:**
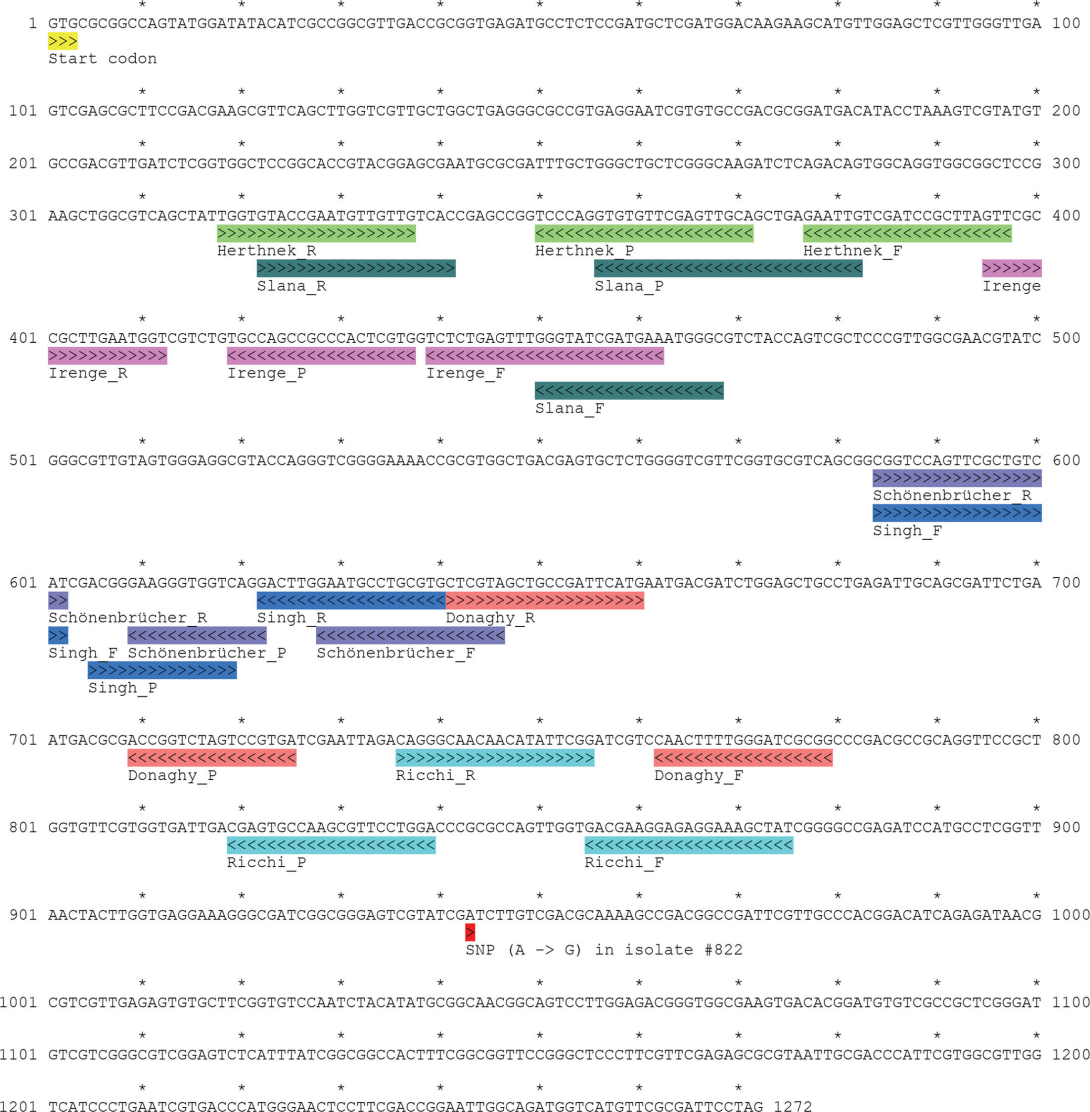
Location of primers and probe from each TaqMan assay design on the GenBank accession no. PP971135 corresponding to the F*57* gene at MAP genomic position 886,451–887,722 (GenBank accession no. NC_002944.2). Primers (F for forward or R for reverse, as described in the original publication) and probe (P) annotations include directionality (>>> for sense and <<< for reverse strand). Genetic variation identified at position 943 (GenBank accession no. PP971134) is represented by a red box. The GTG translation initiation codon (yellow box) of F*57* is at positions 1–3.

The F*57* sequence (GenBank accession no. PP971135) corresponds to a unique locus (positions 886,451–887,722) within the MAP K-10 genome (GenBank accession no. NC_002944.2). This uniqueness was further validated through comparison with the whole-genome sequences from 433 field strains (data not shown). Seven published qPCR designs were mapped along the F*57* gene (see Fig. 2), none of which overlapped with the only genetic variation identified among the field strains; a single-nucleotide polymorphism (A > G) at position 943 in strain AAFC_MAP_#822 (GenBank accession no. PP971134).

An *in silico* BLAST analysis was performed for the seven F*57* TaqMan probe designs, and a summary of the results is provided in Table S3B. Two designs were excluded: F*57-*Schönenbrücher (40) and F*57-*Singh (41). Both utilize short probes (14 and 15 nucleotides, respectively), which exhibited numerous non-specific alignments to microbial genomes, including *Escherichia coli*, a fecal commensal (Table S3B). Such non-specific interactions could impair assay performance by reducing analytical sensitivity (42). The F*57-*Slana design (43) generated the largest amplicon (147 nt, Table S3D). Its reverse primer (Slana_R) and probe (Slana_P) demonstrated low predicted specificity (Table S3B), and the 27-nt-long probe was prone to self-dimerization (ΔG = −10.24 kcal/mol, data not shown). The F*57*-Herthnek (28) and F*57-*Irenge (26) designs (primers and probe) showed the greatest specificity. They overlap the region of the F*57-*Slana amplicon while being both adjacent (Fig. 2) and were selected to develop the multiplex TaqMan qPCR assay. Conversely, the F*57-*Donaghy design (34) was excluded due to poor specificity of both the forward primer and probe (see Table S3B), and a strong tendency to form self-dimers (ΔG = −10.36 and −12.43 kcal/mol, respectively; data not shown). The third F*57* design selected for multiplex testing was the F*57-*Ricchi design (29), which targets a distinct 3′ downstream region of the F*57* gene. According to *in silico* analysis (Table S3B), the F*57-*Ricchi primer-probe sequences are specific to MAP. The three selected TaqMan probe designs, F*57-*Herthnek, F*57-*Irenge, and F*57-*Ricchi, were retained for inclusion in the development of multiplex qPCR assays in combination with other gene targets.

### *In silico* analysis of IS*900* designs

In a previous study (21), we identified the three most robust TaqMan qPCR designs for IS*900* detection as IS*900-*Kim (25), IS*900-*Slana (24), and IS*900-*Herthnek (23). The *in silico* BLAST analysis for the three IS*900* qPCR designs was updated (Table S3C) and includes the identification of a newly reported non-MAP species (*Mycobacterium europaeum*). Although isolated cases of perfect sequence identity were observed between individual assay components (either a primer or probe) and non-MAP species, none of the complete TaqMan designs (i.e., both primers and probe in combination) matched non-target organisms. Using both the reference MAP K-10 genome and the 433 MAP field strains we had previously sequenced, we confirmed *in silico* the presence of 17 copies of IS*900* (data not shown). The characteristics of primers and probes of IS*900-*Kim, IS*900-*Slana, and IS*900-*Herthnek designs are described in Table 1, and their positions were mapped to the IS*900* reference sequence (GenBank accession No. PP417235).

### qPCR efficiency and detection limit

In total, 18 multiplex qPCR assay combinations (3 × 2 × 3) were tested using TaqMan probe qPCR designs including: three IS*900*: IS*900-*Kim (25), IS*900-*Slana (24), and IS*900-*Herthnek (23); two ISMAP*02*: ISMAP*02-*Irenge (26) and ISMAP*02-*Sevilla (27); and three F*57* designs: F*57-*Herthnek (28), F*57-*Irenge (26), and F*57-*Ricchi (29). According to MIQE guidelines, it is mandatory to evaluate the amplification efficiency and the detection limit of qPCR assays. The %Eff of each gene TaqMan design (IS*900*, ISMAP*02,* and F*57*) was calculated for the 18 multiplex qPCR assays using the SensiFAST master mix. The %Eff of amplification of IS*900* (*P* = 0.45), ISMAP*02* (*P* = 0.47), or F*57* (*P* = 0.18) was not affected by multiplexing (Table 2). The SensiFAST master mix provided an excellent %Eff over a broad range of MAP excretion and across all multiplexing qPCR assays, nearing 100% (Table 2). A second parameter to consider when evaluating PCR efficiency is the formation of secondary structures in pooled primers or probes during multiplexing. We calculated the ΔG values for potential primer/probe secondary structures (self-folding or dimerization) in the 18 different multiplex-qPCR combinations (Table S4). No substantial differences were observed among the 18 multiplex-qPCR combinations in terms of number of secondary structures or interaction stability (ΔG).

**TABLE 2:** Efficiency of qPCR of the 18 multiplex qPCR designs^*a*^

Design (IS*900*-ISMAP*02*-F*57*)	IS*900* target	ISMAP*02* target	F*57* target
Herthnek-Irenge-Herthnek	100.0 ± 2.3^a^	98.2 ± 2.7^a^	98.8 ± 2.0^a^
Herthnek-Irenge-Irenge	98.4 ± 1.2^a^	98.7 ± 1.6^a^	103.9 ± 0.3^a^
Herthnek-Irenge-Ricchi	98.7 ± 2.7^a^	100.1 ± 1.9^a^	98.6 ± 3.9^a^
Herthnek-Sevilla-Herthnek	98.2 ± 3.3^a^	96.9 ± 1.2^a^	94.1 ± 0.8^a^
Herthnek-Sevilla-Irenge	97.2 ± 2.1^a^	98.5 ± 1.4^a^	95.5 ± 2.2^a^
Herthnek-Sevilla-Ricchi	99.6 ± 2.4^a^	100.7 ± 1.2^a^	98.7 ± 0.8^a^
Kim-Irenge-Herthnek	100.0 ± 1.4^a^	99.8 ± 0.7^a^	97.5 ± 1.7^a^
Kim-Irenge-Irenge	98.5 ± 1.3^a^	99.6 ± 0.9^a^	101.9 ± 0.5^a^
Kim-Irenge-Ricchi	100.6 ± 1.2^a^	99.2 ± 1.2^a^	100.4 ± 0.8^a^
Kim-Sevilla-Herthnek	98.2 ± 1.0^a^	98.9 ± 2.1^a^	94.6 ± 2.9^a^
Kim-Sevilla-Irenge	98.2 ± 0.9^a^	97.1 ± 1.0^a^	99.7 ± 2.4^a^
Kim-Sevilla-Ricchi	99.9 ± 2.3^a^	100.4 ± 2.5^a^	97.8 ± 0.6^a^
Slana-Irenge-Herthnek	98.1 ± 1.1^a^	99.4 ± 2.6^a^	98.3 ± 1.6^a^
Slana-Irenge-Irenge	97.9 ± 0.8^a^	97.8 ± 1.0^a^	97.5 ± 0.9^a^
Slana-Irenge-Ricchi	103.2 ± 2.6^a^	102.8 ± 1.7^a^	105.3 ± 3.2^a^
Slana-Sevilla-Herthnek	98.5 ± 1.7^a^	98.9 ± 1.6^a^	94.2 ± 3.1^a^
Slana-Sevilla-Irenge	97.8 ± 1.4^a^	97.6 ± 0.6^a^	95.7 ± 0.7^a^
Slana-Sevilla-Ricchi	102.5 ± 1.8^a^	100.9 ± 1.8^a^	105.2 ± 2.6^a^

^*a*^ Percentage of qPCR efficiency was calculated from the slope of a standard curve produced three times (distinct plate assays) for each multiplex design using serial dilutions of MAP K10 DNA. Results are presented as adjusted means ± SE, and mean differences between designs were evaluated using multiple comparisons with a Tukey adjustment; ^a–k^values in the same column with different superscripts differ (*P* < 0.05).

Analytical sensitivity was evaluated by determining the LOD at 50%, 75%, and 95% probability (LOD_50_, LOD_75_, and LOD_95_, respectively) for each assay. The SensiFAST master mix performed well with all multiplex TaqMan assays for detecting all genes (Table 3), and none of the tested combinations failed. The respective analytical sensitivity for the IS*900*, ISMAP*02*, and F*57* genes corresponds to the number of copies of each gene in MAP. Based on the robust gDNA extraction protocol used, it is not surprising that 0.1 Ge of MAP was detected with IS*900* and 0.2 Ge with ISMAP*02*, given that 17 copies of IS*900* and 6 copies of ISMAP*02* are present in the MAP genome. The next step was to evaluate their performance using field samples.

**TABLE 3:** LOD of the 18 multiplex qPCR designs at 50 (LOD_50_), 75 (LOD_75_), and 95% (LOD_95_)^*a*^

	LOD_50_^*b*^			LOD_75_^*c*^			LOD_95_^*d*^		
Design (IS*900*-ISMAP*02*-F*57*)	IS*900* target	ISMAP*02* target	F*57* target	IS*900* target	ISMAP*02* target	F*57* target	IS*900* target	ISMAP*02* target	F*57* target
Herthnek-Irenge-Herthnek	0.5	0.2	2	0.5	0.5	2	1	0.5	10
Herthnek-Irenge-Irenge	0.2	0.2	0.5	0.2	0.5	10	0.5	2	10
Herthnek-Irenge-Ricchi	0.2	0.5	2	1	0.5	10	1	2	10
Herthnek-Sevilla-Herthnek	0.1	0.2	2	0.2	1	2	0.5	2	10
Herthnek-Sevilla-Irenge	0.2	0.5	2	0.5	0.5	2	1	2	10
Herthnek-Sevilla-Ricchi	0.2	0.2	1	0.2	0.5	1	0.5	2	10
Kim-Irenge-Herthnek	0.2	0.2	2	0.2	0.5	10	0.5	1	10
Kim-Irenge-Irenge	0.5	0.5	1	0.5	0.5	2	0.5	1	10
Kim-Irenge-Ricchi	0.2	0.2	0.5	0.2	1	10	0.5	1	10
Kim-Sevilla-Herthnek	0.2	0.2	1	0.2	1	10	0.2	1	10
Kim-Sevilla-Irenge	0.1	0.5	2	0.5	0.5	10	0.5	1	10
Kim-Sevilla-Ricchi	0.1	0.5	2	0.2	0.5	10	0.5	0.5	10
Slana-Irenge-Herthnek	0.2	0.2	2	0.5	0.5	10	0.5	2	10
Slana-Irenge-Irenge	0.2	0.5	10	0.2	0.5	10	0.5	1	10
Slana-Irenge-Ricchi	0.1	0.5	1	0.5	0.5	10	1	1	10
Slana-Sevilla-Herthnek	0.1	0.2	2	0.2	1	1	0.2	2	10
Slana-Sevilla-Irenge	0.1	0.5	2	0.2	0.5	1	1	1	10
Slana-Sevilla-Ricchi	0.1	0.2	1	0.5	0.5	10	0.5	1	10

^*a*^ The LOD was established using serial dilutions of MAP K-10 DNA. Seven replicates of 0.1, 0.2, 0.5, 1, and 2 Ge of MAP were used to evaluate the LOD. Three replicates of 10 Ge of MAP were also analyzed.

^*b*^ The LOD_50_ is equivalent to positive detection of MAP in four replicates out of the seven. The lowest amount of MAP (Ge) yielding LOD_50_ is reported for each gene target (IS*900*, ISMAP*02*, and F*57*) for the respective multiplex qPCR design.

^*c*^ The LOD_75_ is equivalent to positive detection of MAP in six replicates out of the seven. The lowest amount of MAP (Ge) yielding LOD_75_ is reported for each gene target (IS*900*, ISMAP*02*, and F*57*) for the respective multiplex qPCR design.

^*d*^ The LOD_95_ is equivalent to positive detection of MAP in all replicates. The lowest amount of MAP (Ge) yielding LOD_95_ is reported for each gene target (IS*900*, ISMAP*02*, and F*57*) for the respective multiplex qPCR design.

### Evaluation of diagnostic sensitivity using field samples

To evaluate the analytical sensitivity of the 18-qPCR multiplex in complex samples, we included fecal samples with low (F05–F10), moderate (F11–F16), and high (F17–F25) levels of MAP (Fig. S1A through C, respectively), as well as two environmental samples from each of four herds (E01–E08, Fig. S1D). Cows classified as high MAP shedders (F17–F25) exhibited a mean Cq value of 22.21 ± 1.58 using the VetMAX assay (Fig. S1C). Significant differences in amplification efficiency were observed across designs targeting IS*900*, ISMAP*02*, or F*57* (*P* ˂ 0.0001, Table 4). Multiple comparisons for the IS*900* target revealed that the IS*900-*Kim demonstrated greater sensitivity compared to the IS*900-*Herthnek and IS*900-*Slana designs. No significant interaction effects were observed between IS*900* and ISMAP*02* (*P* = 0.1829) or F57 (*P* = 0.0620) designs. Representative qPCR results of fecal samples with high MAP levels (F17–F25) are provided in Fig. S2. For ISMAP*02*, ISMAP*02*-Irenge outperformed ISMAP*02*-Sevilla (*P* < 0.05), though VetMAX Cq values fell within the confidence intervals (*CIs*) of both designs (Table 4).

**TABLE 4:** Results of the 18 qPCR multiplex assays for the detection of MAP in fecal samples from cows excreting high levels of MAP (F17–F25)^*a*^

Design (IS*900*-ISMAP*02*-F*57*)	IS*900* target		ISMAP*02* target^*b*^		F*57* target	
Herthnek-Irenge-Herthnek	20.66 ± 0.52^cdefghi^	(19.64, 21.68)	21.82 ± 0.59^ef^	(20.64, 22.99)	23.78 ± 0.52^ef^	(22.76, 24.80)
Herthnek-Irenge-Irenge	20.95 ± 0.58^abc^	(19.81, 22.09)	21.92 ± 0.56^ef^	(20.82, 23.02)	23.71 ± 0.51^fgh^	(22.69, 24.72)
Herthnek-Irenge-Ricchi	20.57 ± 0.56^efghij^	(19.46, 21.67)	21.79 ± 0.53^f^	(20.75, 22.84)	23.57 ± 0.53^hi^	(22.54, 24.61)
Herthnek-Sevilla-Herthnek	20.84 ± 0.59^bcdeg^	(19.67, 22.00)	22.13 ± 0.55^abcd^	(21.05, 23.21)	23.95 ± 0.54^abcd^	(22.88, 25.02)
Herthnek-Sevilla-Irenge	21.13 ± 0.55^ab^	(20.04, 22.22)	22.20 ± 0.55^abc^	(21.11, 23.29)	23.93 ± 0.53^bc^	(22.89, 24.97)
Herthnek-Sevilla-Ricchi	21.17 ± 0.57^a^	(20.05, 22.29)	22.14 ± 0.55^abcd^	(21.06, 23.21)	23.85 ± 0.54^bcdef^	(22.79, 24.90)
Kim-Irenge-Herthnek	20.32 ± 0.53^j^	(19.27, 21.36)	21.86 ± 0.52^ef^	(20.84, 22.88)	23.61 ± 0.50^hi^	(22.63, 24.60)
Kim-Irenge-Irenge	20.41 ± 0.53^ij^	(19.36, 21.46)	22.01 ± 0.52^cde^	(20.98, 23.04)	23.54 ± 0.51^i^	(22.53, 24.55)
Kim-Irenge-Ricchi	20.31 ± 0.52^j^	(19.27, 21.34)	21.87 ± 0.52^ef^	(20.84, 22.90)	23.35 ± 0.50^j^	(22.36, 24.35)
Kim-Sevilla-Herthnek	20.43 ± 0.52^hij^	(19.41, 21.46)	22.06 ± 0.51^abcde^	(21.05, 23.06)	23.80 ± 0.49^cdefg^	(22.82, 24.77)
Kim-Sevilla-Irenge	20.48 ± 0.53^ghijk^	(19.44, 21.53)	22.06 ± 0.52^bcde^	(21.04, 23.08)	23.72 ± 0.50^efgh^	(22.74, 24.69)
Kim-Sevilla-Ricchi	20.32 ± 0.51^j^	(19.32, 21.32)	22.06 ± 0.52^bcde^	(21.02, 23.10)	23.50 ± 0.51^i^	(22.49, 24.51)
Slana-Irenge-Herthnek	20.74 ± 0.56^cdefgh^	(19.63, 21.85)	21.85 ± 0.61^def^	(20.66, 23.05)	23.87 ± 0.53^bcde^	(22.83, 24.91)
Slana-Irenge-Irenge	20.50 ± 0.61^fhijk^	(19.30, 21.70)	21.83 ± 0.59^ef^	(20.66, 23.00)	23.75 ± 0.50^defg^	(22.77, 24.73)
Slana-Irenge-Ricchi	20.58 ± 0.59^defghij^	(19.41, 21.75)	22.04 ± 0.52^bcde^	(21.01, 23.07)	23.58 ± 0.54^ghi^	(22.51, 24.65)
Slana-Sevilla-Herthnek	20.74 ± 0.53^cdefg^	(19.69, 21.78)	22.19 ± 0.54^abc^	(21.13, 23.26)	24.07 ± 0.53^a^	(23.03, 25.12)
Slana-Sevilla-Irenge	20.86 ± 0.53^abcdef^	(19.81, 21.91)	22.27 ± 0.54^a^	(21.22, 23.33)	23.97 ± 0.52^ab^	(22.95, 25.00)
Slana-Sevilla-Ricchi	20.90 ± 0.54^abcd^	(19.83, 21.97)	22.24 ± 0.55^ab^	(21.16, 23.32)	23.95 ± 0.51^ab^	(22.94, 24.96)

^*a*^ qPCR cycle of quantification values obtained for each target and multiplex assay is presented as adjusted means ± SE, and 95% CI are indicated in parentheses; mean differences between designs were evaluated using multiple comparisons with a Tukey adjustment; ^a–k^values in the same column with different superscripts differ (*P* < 0.05).

^*b*^ The mean Cq value (± SD) using the VetMAX-Gold MAP Detection Kit was 22.22 (± 1.58).

For samples from animals excreting moderate levels of MAP, all multiplex PCR combinations successfully detected the three MAP targets (samples F11–F16, Table S5). However, in samples with low MAP levels (Cq VetMAX > 33; F05–F10, Fig. S1A), significant limitations were observed with certain IS*900* TaqMan designs (Table 5). Specifically, the IS*900-*Kim and IS*900-*Slana designs failed to detect IS*900* in low-level samples (see examples in Fig. S2, F06–F10), whereas the IS*900-*Herthnek design successfully detected IS*900* in all low MAP content cases. Therefore, only IS*900*-Herthnek-based assays were retained for statistical analysis of these low-MAP samples (Table 5), which revealed that diagnostic sensitivity was not compromised by multiplexing with ISMAP*02* and F*57* (*P* < 0.05). The detection rates for IS*900* in low MAP content samples (F06–F10) were 0% for IS*900*-Kim, 3% for IS*900*-Slana, and 81% for IS*900*-Herthnek (Chi-square test, *P* < 0.0001), indicating that the IS*900-*Kim and IS*900-*Slana designs are not suitable for multiplexing for detecting low levels of MAP shedding.

**TABLE 5:** Results of the 18 qPCR multiplex assays for the detection of MAP in fecal samples from cows excreting low levels of MAP (F05–F10)^*a*^

Design (IS*900*-ISMAP*02*-F*57*)	IS*900* target		ISMAP*02* target^*b*^		F*57* target	
Herthnek-Irenge-Herthnek	36.10 ± 2.22^a^	(31.52, 40.67)	27.36 ± 2.30^bc^	(22.75, 31.98)	37.03 ± 0.74^cde^	(35.55, 38.52)
Herthnek-Irenge-Irenge	37.04 ± 2.66^a^	(31.57, 42.52)	29.76 ± 5.16^abc^	(19.41, 40.11)	36.84 ± 0.49^cde^	(35.85, 37.83)
Herthnek-Irenge-Ricchi	36.01 ± 2.05^a^	(31.78, 40.23)	27.10 ± 2.18^c^	(22.73, 31.47)	39.62 ± 1.07^abc^	(37.47, 41.76)
Herthnek-Sevilla-Herthnek	36.12 ± 2.10^a^	(31.80, 40.44)	37.18 ± 1.54^a^	(34.10, 40.27)	36.34 ± 1.27^cde^	(33.80, 38.89)
Herthnek-Sevilla-Irenge	35.47 ± 2.09^a^	(31.17, 39.77)	36.84 ± 1.37^a^	(34.09, 39.60)	36.81 ± 1.40^bcde^	(34.00, 39.61)
Herthnek-Sevilla-Ricchi	35.42 ± 2.09^a^	(31.12, 39.73)	37.41 ± 2.40^ab^	(32.59, 42.23)	42.30 ± 0.86^ab^	(40.56, 44.03)
Kim-Irenge-Herthnek	n.d.		29.57 ± 4.77^abc^	(20.00, 39.15)	38.56 ± 0.65^bc^	(37.24, 39.87)
Kim-Irenge-Irenge	n.d.		29.23 ± 3.95^abc^	(21.31, 37.15)	36.56 ± 0.26^cde^	(36.05, 37.08)
Kim-Irenge-Ricchi	n.d.		28.46 ± 5.80^abc^	(16.81, 40.10)	42.64 ± 0.36^a^	(41.91, 43.36)
Kim-Sevilla-Herthnek	n.d.		38.21 ± 1.21^a^	(35.78, 40.64)	35.32 ± 0.26^e^	(34.79, 35.85)
Kim-Sevilla-Irenge	n.d.		38.66 ± 1.51^a^	(35.63, 41.68)	35.96 ± 0.39^cde^	(35.19, 36.74)
Kim-Sevilla-Ricchi	n.d.		37.04 ± 1.16^a^	(34.72, 39.36)	42.53 ± 1.13^ab^	(40.27, 44.80)
Slana-Irenge-Herthnek	n.d.		27.41 ± 2.27^bc^	(22.87, 31.96)	37.14 ± 0.51^cde^	(36.11, 38.16)
Slana-Irenge-Irenge	n.d.		27.16 ± 2.12^c^	(22.90, 31.42)	37.40 ± 0.45^cd^	(36.50, 38.31)
Slana-Irenge-Ricchi	n.d.		27.16 ± 2.18^c^	(22.79, 31.54)	41.51 ± 1.60^abc^	(38.29, 44.74)
Slana-Sevilla-Herthnek	n.d.		37.02 ± 1.48^a^	(34.05, 39.99)	35.43 ± 0.47^de^	(34.48, 36.38)
Slana-Sevilla-Irenge	n.d.		36.88 ± 1.57^a^	(33.73, 40.02)	35.52 ± 0.86^cde^	(33.79, 37.24)
Slana-Sevilla-Ricchi	n.d.		37.16 ± 1.62^a^	(33.90, 40.41)	39.02 ± 1.60^abcde^	(35.80, 42.24)

^*a*^ qPCR cycle of quantification values obtained for each target and multiplex assay are presented as adjusted means ± SE, and 95% CI are indicated in parentheses; mean differences between designs were evaluated using multiple comparisons with a Tukey adjustment; ^a–e^values in the same column with different superscripts differ (*P* < 0.05); n.d., not detected.

^*b*^ The mean Cq value (± SD) using the VetMAX-Gold MAP Detection Kit was 36.22 (± 1.77).

Another inconsistency was observed when quantifying low-abundance MAP samples (F05–F10, Fig. S2). Although VetMAX Cq values (Cq = 36.2 ± 1.69) fell within *CIs* for ISMAP*02*-Sevilla assays, they were outside the CI range for ISMAP*02*-Irenge (Table 5). ISMAP*02*-Irenge yielded significantly lower Cq values (ΔCq = 6–7 cycles relative to IS*900*), independent of the F*57* or IS*900* multiplex combination used along with it. In multiplexed qPCR combinations, ISMAP*02*-Irenge showed lower concordance with IS*900* detection in low-MAP samples, with only 50% of samples positive compared to 89% with ISMAP*02*-Sevilla (Chi-square, *P* < 0.0001). Detection of F*57*, a single-copy gene, proved challenging in samples with low MAP content, with no significant differences between TaqMan designs (Chi-square test, *P* = 0.32).

While conducting environmental analysis using the different multiplex qPCR combinations, compatibility issues, especially in the case of low MAP content samples, were also observed for the IS*900*-Kim, IS*900*-Slana, and ISMAP*02*-Irenge designs (see Fig. S2 V-AC: samples E01–E08). In low-MAP content samples (e.g., Fig. S2 V, E01; VetMAX Cq = 35), IS*900*-Kim and IS*900*-Slana failed to detect MAP. In environmental MAP samples (E02–E08), no significant differences were attributed to the F*57* design (Table 6). However, ISMAP*02*-Irenge produced Cq values approximately two cycles lower than ISMAP02-Sevilla (a fourfold difference), with VetMAX Cq values (28.4 ± 1.9) falling outside the CI range for ISMAP*02*-Irenge (Table 6). These observations were corroborated using 14 additional environmental samples (E09–E22, data not shown), where IS*900*-Kim and IS*900*-Slana failed to detect IS*900* in seven and five low-MAP samples, respectively. ISMAP*02*-Irenge also overestimated ISMAP*02* abundance relative to IS*900* in 6 of 14 samples. In contrast, the ISMAP*02-*Sevilla multiplex assays consistently produced results that aligned closely with those obtained using the USDA-licensed commercial VetMAX qPCR assay.

**TABLE 6:** Results of the 18 qPCR multiplex assays for the detection of MAP in environmental samples (E02–E08)^*a*^

Design (IS*900*-ISMAP*02*-F*57*)	IS*900* target^*b*^		ISMAP*02* target^*c*^		F*57* target	
Herthnek-Irenge-Herthnek	26.71 ± 0.66^bcf^	(25.39, 28.02)	26.28 ± 0.46^ab^	(25.38, 27.19)	29.88 ± 0.84^a^	(28.22, 31.54)
Herthnek-Irenge-Irenge	26.82 ± 0.68^abcde^	(25.47, 28.17)	26.52 ± 0.51^ab^	(25.50, 27.54)	29.84 ± 0.83^a^	(28.21, 31.48)
Herthnek-Irenge-Ricchi	26.69 ± 0.62^cf^	(25.46, 27.92)	26.37 ± 0.45^ab^	(25.47, 27.28)	30.56 ± 1.45^a^	(27.69, 33.44)
Herthnek-Sevilla-Herthnek	27.05 ± 0.64^abde^	(25.79, 28.31)	28.07 ± 0.64^ab^	(26.79, 29.34)	29.84 ± 0.69^a^	(28.46, 31.21)
Herthnek-Sevilla-Irenge	27.08 ± 0.66^ade^	(25.77, 28.40)	28.07 ± 0.67^ab^	(26.74, 29.40)	29.84 ± 0.80^a^	(28.26, 31.42)
Herthnek-Sevilla-Ricchi	27.13 ± 0.65^ade^	(25.85, 28.42)	28.05 ± 0.66^ab^	(26.74, 29.37)	30.25 ± 1.07^a^	(28.14, 32.37)
Kim-Irenge-Herthnek	27.09 ± 1.24^abcde^	(24.64, 29.55)	26.43 ± 0.59^ab^	(25.25, 27.60)	30.50 ± 1.11^a^	(28.31, 32.70)
Kim-Irenge-Irenge	27.36 ± 1.30^abcde^	(24.78, 29.94)	26.60 ± 0.55^ab^	(25.50, 27.70)	30.04 ± 0.87^a^	(28.31, 31.76)
Kim-Irenge-Ricchi	26.87 ± 1.06^ef^	(24.77, 28.96)	26.45 ± 0.55^ab^	(25.37, 27.54)	31.08 ± 1.29^a^	(28.52, 33.64)
Kim-Sevilla-Herthnek	26.68 ± 0.90^abcde^	(24.89, 28.47)	28.01 ± 0.74^ab^	(26.55, 29.48)	29.77 ± 0.83^a^	(28.12, 31.42)
Kim-Sevilla-Irenge	26.88 ± 1.01^abcde^	(24.87, 28.89)	28.05 ± 0.75^ab^	(26.57, 29.53)	29.88 ± 0.88^a^	(28.14, 31.62)
Kim-Sevilla-Ricchi	26.39 ± 0.83^df^	(24.75, 28.03)	28.05 ± 0.75^ab^	(26.57, 29.53)	30.25 ± 1.20^a^	(27.86, 32.64)
Slana-Irenge-Herthnek	27.40 ± 1.09^abcde^	(25.23, 29.57)	26.57 ± 0.49^a^	(25.60, 27.54)	29.93 ± 0.75^a^	(28.45, 31.42)
Slana-Irenge-Irenge	26.97 ± 0.97^abcde^	(25.05, 28.89)	26.40 ± 0.51^b^	(25.39, 27.42)	29.79 ± 0.86^a^	(28.08, 31.50)
Slana-Irenge-Ricchi	27.40 ± 1.15^abcde^	(25.13, 29.68)	26.53 ± 0.45^ab^	(25.64, 27.43)	29.93 ± 0.95^a^	(28.04, 31.82)
Slana-Sevilla-Herthnek	26.88 ± 0.78^abce^	(25.34, 28.42)	28.10 ± 0.67^ab^	(26.77, 29.44)	29.80 ± 0.66^a^	(28.49, 31.10)
Slana-Sevilla-Irenge	27.11 ± 0.90^abce^	(25.32, 28.89)	28.22 ± 0.68^ab^	(26.87, 29.56)	29.82 ± 0.72^a^	(28.39, 31.24)
Slana-Sevilla-Ricchi	27.52 ± 1.12^abcd^	(25.30, 29.75)	28.24 ± 0.70^ab^	(26.86, 29.62)	30.21 ± 0.91^a^	(28.40, 32.01)

^*a*^ qPCR cycle of quantification values obtained for each target and multiplex assay are presented as adjusted means ± SE, and 95% CI are indicated in parentheses; mean differences between designs were evaluated using multiple comparisons with a Tukey adjustment; ^a–f^values in the same column with different superscripts differ (*P* < 0.05).

^*b*^ The sample E02 was excluded from the multiple comparisons for IS*900,* as most of the “Kim”-multiplexed assays failed.

^*c*^ The mean Cq value (± SD) using the VetMAX-Gold MAP Detection Kit was 28.45 (± 1.89).

### Validation of the qPCR multiplex

**Fig 3:**
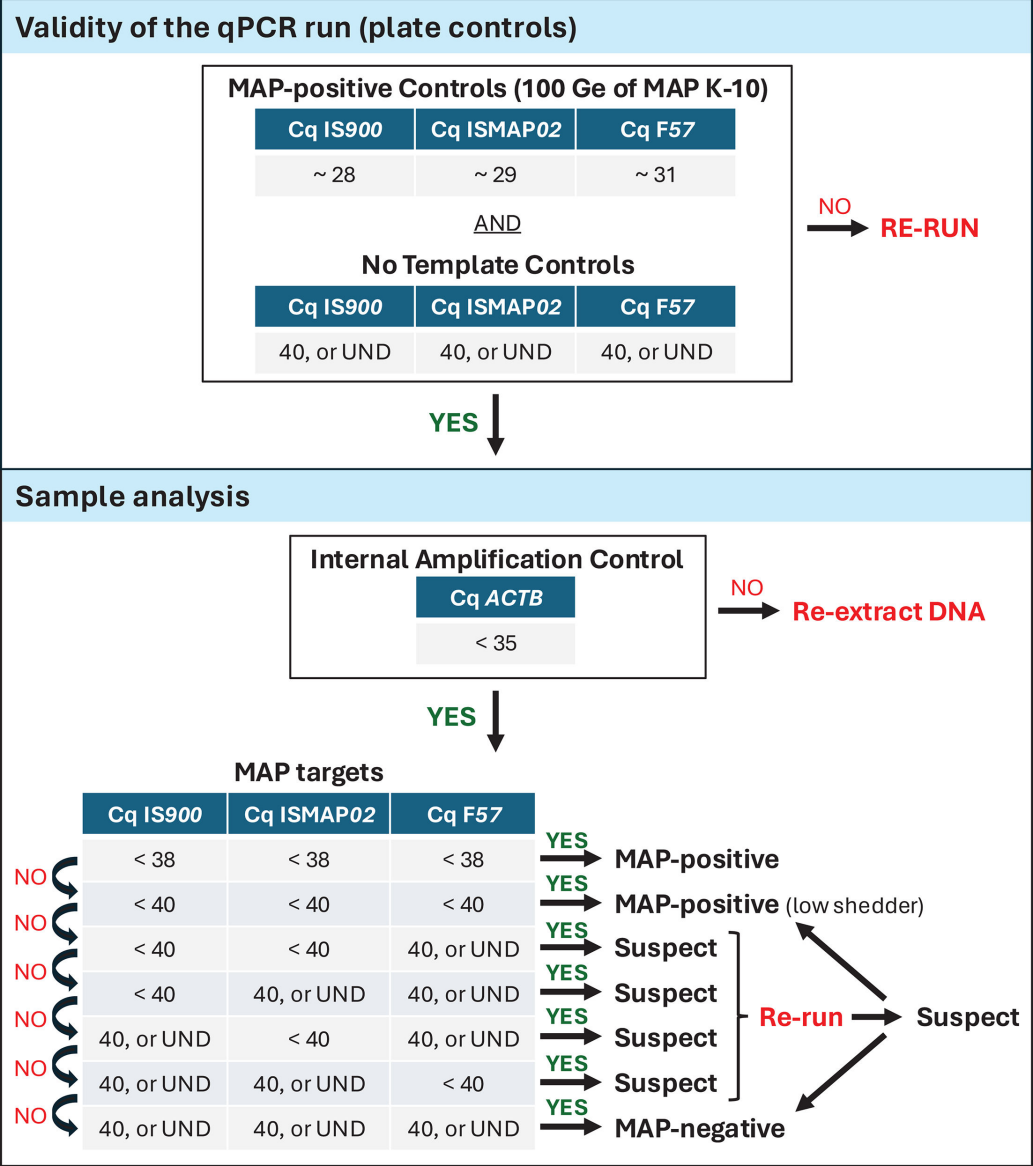
Decisional algorithm for analyzing fecal or environmental samples by multiplex qPCR (IS*900*-Herthnek, ISMAP*02*-Sevilla, and F*57*-Herthnek). The protocol follows a two-step approach. First, the integrity of the 96-well plate setup is verified through the inclusion of both negative and positive controls. Second, the validity of the qPCR results from each sample is confirmed using an IAC (*ACTB*), which serves to monitor the efficiency of both DNA extraction and qPCR amplification. This algorithm does not allow for the detection of non-MAP pathogens. However, it could potentially indicate the presence of MAH pathogens if a clear detection of ISMAP*02*—outside the low-abundance MAP range of the shedding spectrum—is observed in the absence of F*57* and IS*900*, which would warrant further investigation of the animal. This decisional algorithm ensures robust and reliable interpretation of multiplex-qPCR data across diverse sample types. The algorithm offers a streamlined and practical framework for interpreting multiplex-qPCR results, balancing analytical rigor with operational efficiency. UND, undetermined result (no quantitative signal detected).

For validating the diagnostic sensitivity of our final version of the multiplex qPCR, results obtained with the IS*900*-Herthnek, ISMAP*02*-Sevilla, and F*57*-Herthnek qPCR assays were compared to those obtained from individual simplex qPCRs. The Cq values of each target in the multiplex qPCR were strongly correlated with those of the corresponding simplex qPCR, for IS*900* (r_c_ *=* 0.9956), ISMAP*02* (r_c_ = 0.9960), and F*57* (r_c_ = 0.9858). We developed a decision-making algorithm (Fig. 3), which was validated using an independent data set comprising 2,110 dairy cows and 463 environmental samples (see Table S6A and C). Our finalized multiplex qPCR assay demonstrated high analytical specificity and sensitivity across MAP genotypes, detecting all C-type (Type II) strains collected from herds (GenBank BioProject PRJNA925907). *In silico* analyses confirmed that IS*900*-Herthnek detected all 17 IS*900* copies in bison-type strains (MAPK J) and all 22 in Telford S-type (subtype I) sequences with 100% identity. ISMAP*02*-Sevilla also detected all six MAPK J and seven Telford S-type ISMAP*02* copies with perfect match, while F*57*-Herthnek detected the single F57 locus with 100% identity. Therefore, our multiplex TaqMan assay is suitable for detecting cases of Bison-type MAP infection, a subcategory of C strain/Type II lineage strains, and for the detection of infection in sheep or goats (Telford S-type, subtype I). The detection limit of the multiplex qPCR assay was 0.5 genome equivalent of MAP bacteria (Table 3), which is equivalent to one IS*900* DNA copy/μL in 95% of reactions. With the detection of ISMAP*02* (LOD of 2 Ge of MAP; 1.5 copies/μL in 95% of the reactions) and F*57* (LOD of 10 MAP; one copy/μL in 95% of the reactions), the multiplex qPCR assay provides a highly sensitive and specific tool for detecting MAP in diverse sample types and infection contexts.

### *ACTB* as a DNA extraction control

The SensiFAST qPCR mix offers the advantage of accommodating a fourth detection channel (ROX), which can be utilized to monitor failures in either gDNA extraction or qPCR amplification. Given the pervasive presence of bovine tissue residues on dairy farms, we sought to evaluate whether the bovine *ACTB* gene could serve as an IAC for gDNA extraction and amplification. The 18 multiplex qPCR combinations included *ACTB* as both a DNA extraction and an internal qPCR amplification control. *ACTB* amplification was consistent and reliable across all sample types, with a mean Cq of 27.43 ± 1.19 for fecal samples and 25.14 ± 1.33 for environmental samples. The inclusion of *ACTB* confirmed the functionality of all PCRs, regardless of the multiplex combination used. No significant differences were observed between the multiplex qPCR combinations (data not shown), supporting the comparability of results across all combinations. Additionally, *ACTB* levels in samples from JD-negative cows, defined as MAP negative by fecal qPCR and consistently negative by serum ELISA throughout the longitudinal study, were comparable to those in MAP-positive samples. The inclusion of *ACTB* as IAC confirmed the validity of PCR assays even in samples where MAP was not detected.

## DISCUSSION

**Fig 4:**
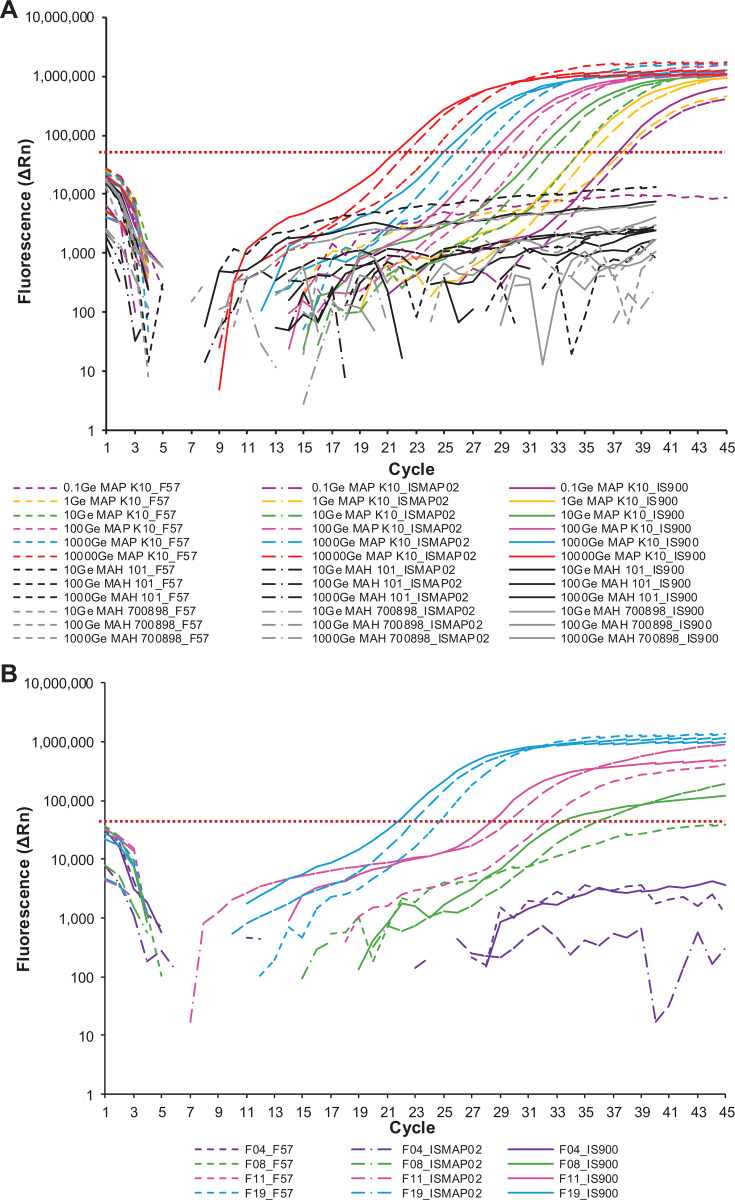
Amplification curves of IS*900*-Herthnek, ISMAP*02*-Sevilla, and F*57*-Herthnek. (**A**) Multiplex-qPCR results were obtained using a standard curve generated with MAP K10 Ge at concentrations of 0.1 (purple), 1 (yellow), 10 (green), 100 (pink), 1,000 (blue), and 10,000 (red). MAH 101 (black) and MAH 700898 (gray) were also analyzed at 10, 100, and 1,000 Ge. All samples were processed under identical qPCR conditions, except for the MAH samples, which were run with 40 cycles. (**B**) Field samples representing different levels of MAP shedding—MAP negative (F04, purple), low (F08, green), moderate (F11, pink), and high (F19, blue)—were analyzed using multiplex-qPCR. Targets included IS*900* (solid line), ISMAP*02* (dot-dash line), and F*57* (dashed line), with the threshold indicated by a dotted horizontal line. The x-axis represents the number of cycles, and the y-axis shows ΔRn (normalized fluorescence value, log scale).

Using pure MAP DNA, all 18 multiplex qPCR assays demonstrated high and comparable analytical sensitivity. The 95% LODs (LOD₉₅) were determined to be 0.5 Ge for IS*900* and 2.0 for ISMAP*02*, indicating robust detection in 95% of replicates. For F*57*, all designs had a LOD₉₅ of 10 Ge. The relative sensitivity (Cq values) of the targets generally followed the expected trend: F*57* > ISMAP*02* > IS*900*. Using the optimal multiplex-qPCR assay (IS*900*-Herthnek/ISMAP*02*-Sevilla/F*57*-Herthnek), this pattern was confirmed for the standard curve and most MAP positive samples with moderate and high levels of bacterial loads (Fig. 4). This is clearly illustrated by the amplification curves, which show the relative increase in fluorescence for IS*900* (solid line), ISMAP*02* (dot-dash line), and F*57* (dashed line).

We developed a decision-making algorithm to ensure robust and reliable interpretation of multiplex qPCR data across diverse sample types. This algorithm was validated on 2,110 cows confirmed positive or negative for MAP infection during the longitudinal study and also validated on environmental samples collected from dairy herds with paratuberculosis (Table S6). Given that F*57* exists as a single copy, a higher LOD threshold is expected based on Poisson distribution principles. In low-MAP content samples such as those detected by ISMAP*02* with VetMAX Cq values >35, the absence of F*57* detection is not surprising. A ΔCq of 2.58 between ISMAP*02* and F*57* corresponds to a substantial reduction in F*57* template availability, leading to signal loss due to stochastic effects in low-copy number scenarios. Examples of such results are shown in Table S6B, where ΔCq values in red do not quite correspond to the expected ratio of gene copy number in the genome. This discrepancy is seen to occur only with samples containing few MAP. An example of such loss of the F*57* signal can be visualized by the amplification plots of qPCR results from a low-MAP sample shown in Fig. 4B (sample F08), where IS*900* and ISMAP*02* were detected but F*57* never crossed the detection threshold. At low MAP abundance, where concentrations of MAP DNA templates are low, PCR amplification becomes a stochastic process. In this detection range, the probability of amplifying each target molecule follows a Poisson distribution, with each target representing an independent probabilistic event. This randomness can lead to variability in detection, where the presence or absence of amplification for each target depends on whether at least one copy was included in the reaction mixture (in the 8 μL picked from the fecal DNA extract). Therefore, for low MAP samples, it is not unusual for the Cq values to deviate from the expected F*57* > ISMAP*02* > IS*900* rule.

In probe-based assays, the elevated concentrations of primers and probes can promote the formation of primer dimers (homodimers or heterodimers), which may significantly compromise PCR efficiency (16). To assess this potential interference, we evaluated the propensity for secondary structure formation, including hairpins, homodimers, and heterodimers, using Gibbs free energy (ΔG) as a predictive metric. A ΔG value below a defined threshold indicates that such structures are thermodynamically favorable and therefore likely to impair qPCR performance (42). However, our analysis revealed no correlation between ΔG values (either minimum or average) or the number of predicted secondary structures and the empirical performance of the qPCR designs. Notably, all TaqMan designs performed well in simplex (data not shown) and across all 18 multiplex qPCR combinations tested, exhibiting near-perfect PCR efficiencies, approaching 100%. Despite this, certain qPCR designs using field samples negatively impacted the diagnostic sensitivity of the multiplex assays, indicating that factors beyond PCR efficiency, such as cross-reactivity or non-specific amplification, may influence assay performance. The causes of the false-negative results with IS*900*-Kim and IS*900*-Slana have not been elucidated, but it is possible that the lack of sensitivity is due to nonspecific interactions, which we have not demonstrated.

When evaluating diagnostic sensitivity, it is essential to validate that PCR inhibitors do not bias MAP detection. It is also important to include animals exhibiting varying levels of MAP shedding to ensure that the assay performs reliably across the full spectrum of sample types it is intended to assess (44). The use of fecal or environmental samples for diagnostics presents two main challenges. First, efficient DNA extraction from mycobacteria can be difficult, and the presence of PCR inhibitors in these complex matrices may compromise amplification. Second, the inherent complexity of these samples, rich in diverse biological materials and microorganisms, can lead to non-specific interactions. These factors may ultimately reduce diagnostic sensitivity (45). For fecal PCR, we previously demonstrated that the Quick-DNA Fecal/Soil Microbe Miniprep Kit protocol offers superior sensitivity. We previously used two approaches to validate that DNA extracts were free from PCR inhibitors. The first and most straightforward strategy involved performing 10-fold serial dilutions of the DNA extract to estimate the presence of PCR inhibitors. We compared the Quick-DNA Fecal/Soil Microbe Miniprep Kit to two other commercially available DNA extraction methods. A series of 1:10 dilutions of fecal samples reported that using this approach, the Quick-DNA Fecal/Soil Microbe Miniprep Kit showed the expected 3.3-Ct difference between the 1:10 dilutions (9). In contrast, two other commercially available DNA extraction methods did not yield the expected ΔCt values, as the ΔCt was reduced to ~1 (i.e., improved detection), suggesting that dilution attenuated the inhibitory effects on qPCR. A second validation was performed by evaluating PCR efficiency (%Eff) using standard curves prepared from pure MAP genomic DNA (serial dilutions equivalent to 0.1, 0.5, 1, 10, 20, 100, 200, 1,000, 2,000, and 10,000 Ge). The same standard curve was spiked into the fecal DNA extract (8 μL in a final 25 μL PCR). The presence of fecal DNA extract did not affect %Eff (Fig. 4, in reference [21]), confirming that the extraction protocol effectively removed PCR inhibitors. The enhanced performance of the Quick-DNA Fecal/Soil Microbe Miniprep Kit is largely due to the mechanical bead-beating step, which effectively disrupts the thick, waxy mycobacterial cell wall. Combined with spin column-based DNA purification, this method enables the elution of MAP and fecal DNA, free of PCR inhibitors. In the present study, we applied the same rigorous DNA extraction and purification protocol to evaluate 18 multiplex qPCR assay combinations.

We previously validated the TaqMan Environmental Master Mix (EnviMM) for its robust performance using feces collected directly from animals or from the dairy farm environment (21). EnviMM includes a ROX fluorophore (emission at 617 nm), a passive internal reference dye commonly used to correct for inter-well variation and pipetting inconsistencies. The SensiFAST master mix demonstrated performance comparable to EnviMM across all targets (F*57*, ISMAP*02*, and IS*900*; data not shown), while also allowing the inclusion of an IAC. By selecting a master mix without an internal reference dye (e.g., ROX), the fourth detection channel could be repurposed to monitor extraction and amplification efficiency. The choice of *ACTB* as the IAC was particularly appropriate, given its ubiquitous presence in both fecal and environmental matrices. The *ACTB* gDNA was consistently detected in all 1,737 environmental samples (data not shown) and in all 2,110 dairy cows (independent population, data not shown) tested by us to date, without exception. *ACTB* was found to be relatively stable. In addition, *ACTB* as the IAC is especially valuable in the context of MAP detection, where target abundance is often low. In the absence of the MAP signal, successful amplification of *ACTB* confirms the integrity of both the DNA extraction and qPCR detection protocols.

In the past, the diagnostic specificity of IS*900*-based assays for MAP detection was questioned due to the presence of IS*900*-like sequences in non-MAP mycobacteria (46–49). However, with the availability of the complete MAP genome and those from many other mycobacteria, it is now possible to confirm that IS*900* detection assays are specific for MAP. *In silico* BLAST analyses confirmed the analytical specificity of these IS*900* assays, namely IS9*00*-Herthnek, IS*900-*Kim, and IS*900-*Slana. They do not share sequence similarity with IS*900*-like elements found in MAC, notably IS*1613* (GenBank acc. No. AJ011837.1), IS*1626* (GenBank acc. No. AF071067.1), IS*901*/*902* (GenBank acc. No. X58030.1), IS*1110* (GenBank acc. No. Z23003.1), and IS*110* (GenBank acc. No. Z23003.1) (data not shown). However, some of these MAP-specific IS*900* assays lacked diagnostic sensitivity, especially when applied to samples with low MAP content. For instance, the IS*900*-Kim and IS*900*-Slana assays failed to detect MAP in low-MAP samples. In a previous study using singleplex qPCR, we observed that IS*900*-Kim and IS*900*-Slana failed to detect MAP in some low-shedding cows (21). In the present study, false negatives with IS*900*-Kim and IS*900*-Slana were also observed when these assays were multiplexed. That failure is not attributable to interactions with other TaqMan probe designs, as no such interactions were observed with pure MAP DNA. Rather, the reduced diagnostic sensitivity of the IS*900*-Kim and IS*900*-Slana assays appears to result from non-specific interactions with fecal or environmental sequences. This limitation was not observed with the IS*900-*Herthnek assay or the USDA-licensed VetMAX commercial assay, both of which consistently demonstrated high diagnostic sensitivity, regardless of the MAP load present in the sample. The MAP-specific target of the commercial USDA-licensed VetMAX assay is not publicly disclosed. Interestingly, testing of MAP-positive field samples (*n* = 181) in parallel with both multiplex-qPCR and VetMAX assays suggests that the VetMAX target gene is ISMAP*02*, based on Cq values. The correlation between Cq values from two assays is 0.96, which increased to 0.99 when considering Cq values <34 (*n* = 102). Our in-house multiplex-qPCR assays are an alternative to the USDA-licensed VetMAX commercial assay that requires an import permit for use in Canada.

Multiplex qPCR provides a major advantage by enabling the simultaneous detection of multiple MAP-specific genetic targets, without compromising sensitivity compared to single-target qPCR (Table S6D). By measuring the cross-sensitivity of the three targets, the multiplex qPCR approach enables a more robust evaluation of the design. For example, the ISMAP*02*-Irenge assay initially appeared more sensitive than the ISMAP*02*-Sevilla assay, as indicated by consistently lower Cq values in both high-MAP (Table 5) and low-MAP (Table 6) content samples. Given the well-characterized MAP genome, the relative abundance of target genes is expected to reflect their genomic copy numbers. Theoretically, the ΔCq between IS*900* (17 copies) and ISMAP*02* (6 copies) should be approximately +1.5 cycles, based on the log₂ ratio of their copy numbers. However, in the ISMAP*02*-Irenge multiplexed assays, low-MAP samples showed ISMAP*02* Cq values that were 6–7 cycles lower than those of IS*900*. This would imply that ISMAP02 is 2^6^–2^7^ times more abundant than IS900, an outcome that contradicts the known genomic structure of MAP. For moderate to high-abundant MAP samples, such an incongruity strongly suggests the presence of assay-related technical problems, possibly related to unspecific primer/probe design, and underscores the need for cautious interpretation of diagnosis results. The discrepancy was also confirmed by comparing results to the F*57* target. While the expected ΔCq between F*57* and ISMAP*02* is approximately 2.6, the ISMAP*02*-Irenge multiplex yielded a ΔCq that often exceeded 7 Cq. In contrast, the ISMAP*02*-Sevilla design produced results consistent with expectations based on IS*900*, F*57*, and the USDA-licensed VetMAX assay. These findings underscore the value of multiplexing targets for detecting inconsistencies that may indicate a potential false-negative result in certain assay designs, thereby enhancing the interpretability and reliability of results.

Another valuable feature of the multiplex qPCR assay is its ability to differentiate MAP from other members of MAC, due to their distinct genomic architectures. For instance, an analysis of 190 MAH genomes from the NCBI database revealed 14 records with 100% query coverage and 100% identity to ISMAP*02* (E-value = 0). These 14 MAH strains contained only one or two copies of ISMAP*02* (data not shown). For the other MAH strains, restricted regions showed similarity with ISMAP*02* (Fig. 1, black boxes), and the absence of the F*57* gene was confirmed for strains 101 and 104. These findings highlight the importance of using a multiplex qPCR assay, especially in environments where MAH strains carrying ISMAP*02* may be present. In animals such as cattle and pigs, MAH is considered an opportunistic pathogen. Therefore, clear detection of ISMAP*02* (outside the low-MAP abundance range of the excretion spectrum) in the absence of F*57* (GenBank acc. no. PP971135) and IS*900* (GenBank acc. no. PP417235) may indicate the presence of MAH strains, warranting further investigation. The NCBI database is continuously expanding, and improvements in sequencing technologies, genome assembly quality, and curation by NCBI staff enhance the reliability of *in silico* BLAST analyses for assessing the analytical specificity of primers and probes. Nonetheless, it remains essential to validate specificity using field samples, which may contain a wide diversity of microorganisms not represented in reference databases.

The ISMAP*02*-Sevilla qPCR design consistently outperformed the TaqMan ISMAP*02*-Irenge design, particularly in fecal and environmental samples with low MAP content. The ISMAP*02*-Irenge design either reported false-negative results or inconsistently overestimated ISMAP*02* abundance in both cow fecal and environmental low-MAP samples. Inconsistent results with IS*900*-Kim and IS*900*-Slana were also observed with low-MAP samples (Fig. S2, F06–F10). Notably, these discrepancies cannot be attributed to herd-specific signatures, as the challenging samples originated from multiple herds (Fig. S1A through C), ruling out geographical or source-related biases. Such inaccuracies were not observed in low-MAP samples when purified MAP DNA was used in single-qPCR (21) or multiplex-qPCR (present study) format. However, when fecal or environmental DNA was present, a lack of sensitivity was observed with low-MAP samples. Fecal or environmental DNA, which contains various biological matrices and microorganisms, may induce non-specific competitive reactions masking MAP amplification because primers are diverted toward non-specific amplification. Therefore, it is important to evaluate assay performance prior to optimization, and not solely with pure MAP DNA. Further optimization—such as adjusting the concentration of primers or probes—may help attenuate non-specific competitive reactions. In the present study, validation was carried out using the recommended probe concentration (400 nM), which is relatively high. Non-specific probes may undergo hydrolysis while annealing to non-MAP sequences, potentially leading to an overestimation of MAP abundance. Nevertheless, the multiplex qPCR demonstrated high stability across a broad range of annealing temperatures (56°C–66°C), and the sensitivity of all three assays remained unaffected (Table S6E). These results suggest that the selected multiplex qPCR designs are highly robust with complex fecal samples. The selected designs, namely IS*900*-Herthnek, ISMAP*02*-Sevilla, and F*57*-Herthnek, consistently produced reliable and specific amplification in both pure and field-derived DNA samples, and results were congruent with expected MAP genomic structure, including the relative abundance of IS*900* and F*57*. Interestingly, the overall results suggest that the target gene of the commercial USDA-licensed VetMAX-Gold MAP Detection kit is ISMAP*02*, based on relative gene intensity detected for all samples (Fig. S2).

### Conclusions

We developed a multiplex qPCR assay incorporating TaqMan designs that include the F*57* (F*57-*Herthnek), ISMAP*02* (ISMAP*02-*Sevilla), and IS*900* (IS*900-*Herthnek) genes for the specific detection of MAP. This assay was evaluated for both analytical sensitivity using pure DNA and diagnostic sensitivity using fecal and environmental samples. While all 18 multiplex combinations demonstrated excellent sensitivity and PCR efficiency with pure DNA, the IS*900*-Herthnek and ISMAP*02*-Sevilla designs showed superior diagnostic sensitivity in detecting low MAP abundance in complex field samples. The inclusion of both markers of repetitive sequence (IS9*00* and ISMAP*02*) and the single-copy F*57* gene enhances the sensitivity and specificity of the assay. In addition, the strength of our approach lies in the use of ΔCq values between target genes as an indicator of diagnostic specificity issues.

Our multiplex qPCR assay demonstrated high specificity for MAP, reliably detecting all C-strain (Type II) strains collected from Canadian dairy herds. It also effectively identified Bison-type MAP, a subgroup within the C-strain/Type II lineage, as well as the S-type (subtype I), typically associated with infections in sheep and goats. Overall, our multiplex qPCR assay provides a robust, highly sensitive, and specific tool for the detection of MAP, supporting accurate diagnosis of JD for both animal and environmental samples.

## Data Availability

All data supporting the findings of this study are available as supplementary files.

## SUPPLEMENTAL MATERIAL

Figure S1 jcm.00872-25-s0001.pdf Quantification of MAP excreted in feces (Cq value) and anti-MAP antibody levels (serum ELISA, %S/P) according to the age of animals of low (A), moderate (B) and high (C) shedders and MAP level (Cq) from the environnemental samples (D).

Figure S2 jcm.00872-25-s0002.pdf Graphs of the qPCR results. All samples (cows and feces) were analyzed using the 18 multiplex-qPDR assays.

Table S1 jcm.00872-25-s0003.docx Profiles (Fecal qPCR [Cq] and serum ELISA) of cows F05-F25 and Environmental qPCR results (Cq values) for E01-E08 samples.

Table S2 jcm.00872-25-s0004.docx Results of literature search: TaqMan designs for ISMA02 and F57.

Table S3 jcm.00872-25-s0005.xlsx Blast results of the primer and probe sequences of the TaqMan assays for ISMAP02 (A), F57 (B), IS900 (C) and unique F57 TaqMan designs (D).

Table S4 jcm.00872-25-s0006.xlsx Secondary structures calculated in silico within each of the 18 multiplex-qPCR assays.

Table S5 jcm.00872-25-s0007.docx Statistical analysis of the qPCR (Cq) results obtained with the moderate MAP shedders using the 18 multiplex-qPCR assays.

Table S6 jcm.00872-25-s0008.xlsx Validation of the final multiplex-qPCR assay. A. individual cows; B. ΔCq analysis; C. environmental samples; D. Simplex vs Multiplex; E. different annealing temperature of the PCR program.

ASM does not own the copyrights to Supplemental Material that may be linked to, or accessed through, an article. The authors have granted ASM a non-exclusive, world-wide license to publish the Supplemental Material files. Please contact the corresponding author directly for reuse.

## References

[1] Agrawal G, Borody TJ, Aitken JM. 2024. Mapping Crohn’s disease pathogenesis with Mycobacterium paratuberculosis: a hijacking by a stealth pathogen. Dig Dis Sci 69:2289–2303. doi:10.1007/s10620-024-08508-4 38896362

[2] Kuenstner JT, Potula R, Bull TJ, Grant IR, Foddai A, Naser SA, Bach H, Zhang P, Yu D, Lu X, Shafran I. 2020. Presence of infection by Mycobacterium avium subsp. paratuberculosis in the blood of patients with Crohn's disease and control subjects shown by multiple laboratory culture and antibody methods. Microorganisms 8:2054. doi:10.3390/microorganisms8122054 33371478 PMC7767509

[3] Greenstein RJ. 2024. Human genetic defects and misinterpreted pharmacological data indicate that Crohn disease is consequent to a mycobacterial infection. MRAJ 12. doi:10.18103/mra.v12i7.5541

[4] Waddell LA, Rajić A, Stärk KDC, McEWEN SA. 2015. The zoonotic potential of Mycobacterium avium ssp. paratuberculosis: a systematic review and meta-analyses of the evidence. Epidemiol Infect 143:3135–3157. doi:10.1017/S095026881500076X 25989710 PMC9150950

[5] More S, Bøtner A, Butterworth A, Calistri P, Depner K, Edwards S, Garin‐Bastuji B, Good M, Gortázar Schmidt C, Michel V, et al.. 2017. Assessment of listing and categorisation of animal diseases within the framework of the Animal Health Law (Regulation (EU) No 2016/429): paratuberculosis. EFS2 15. doi:10.2903/j.efsa.2017.4960 PMC7009931 32625600

[6] Waddell LA, Rajić A, Stärk KDC, McEwen SA. 2016. The potential public health impact of Mycobacterium avium ssp. paratuberculosis: global opinion survey of topic specialists. Zoonoses Public Health 63:212–222. doi:10.1111/zph.12221 26272619

[7] Whittington R. 2020. Cultivation of *Mycobacterium avium* subsp. *paratuberculosis* (Chapter 18). In Behr MA, Stevenson K, Kapur V (ed), Paratuberculosis: organism, disease, control. CABI, Boston, MA; Wallingford, Oxfordshire.

[8] Merkal RS, Lyle P, Whipple DL. 1982. Decontamination, media and culture methods for Mycobacterium paratuberculosis. 86th Annual Meeting of the United States Animal Health Association. p 519–523, Nashville, Tennessee, USA

[9] Fock-Chow-Tho D, Topp E, Ibeagha-Awemu EA, Bissonnette N. 2017. Comparison of commercial DNA extraction kits and quantitative PCR systems for better sensitivity in detecting the causative agent of paratuberculosis in dairy cow fecal samples. J Dairy Sci 100:572–581. doi:10.3168/jds.2016-11384 27889120

[10] Nielsen SS, Toft N. 2008. Ante mortem diagnosis of paratuberculosis: a review of accuracies of ELISA, interferon-gamma assay and faecal culture techniques. Vet Microbiol 129:217–235. doi:10.1016/j.vetmic.2007.12.011 18255239

[11] Whitlock RH, Wells SJ, Sweeney RW, Van Tiem J. 2000. ELISA and fecal culture for paratuberculosis (Johne’s disease): sensitivity and specificity of each method. Vet Microbiol 77:387–398. doi:10.1016/s0378-1135(00)00324-2 11118724

[12] Pinedo PJ, Rae DO, Williams JE, Donovan GA, Melendez P, Buergelt CD. 2008. Association among results of serum ELISA, faecal culture and nested PCR on milk, blood and faeces for the detection of paratuberculosis in dairy cows. Transbound Emerg Dis 55:125–133. doi:10.1111/j.1865-1682.2007.01009.x 18397500

[13] Logar K, Kopinč R, Bandelj P, Starič J, Lapanje A, Ocepek M. 2012. Evaluation of combined high-efficiency DNA extraction and real-time PCR for detection of Mycobacterium avium subsp. paratuberculosis in subclinically infected dairy cattle: comparison with faecal culture, milk real-time PCR and milk ELISA. BMC Vet Res 8:49. doi:10.1186/1746-6148-8-49 22551054 PMC3423054

[14] Kawaji S, Taylor DL, Mori Y, Whittington RJ. 2007. Detection of Mycobacterium avium subsp. paratuberculosis in ovine faeces by direct quantitative PCR has similar or greater sensitivity compared to radiometric culture. Vet Microbiol 125:36–48. doi:10.1016/j.vetmic.2007.05.002 17582709

[15] Plain KM, Marsh IB, Waldron AM, Galea F, Whittington A-M, Saunders VF, Begg DJ, de Silva K, Purdie AC, Whittington RJ. 2014. High-throughput direct fecal PCR assay for detection of Mycobacterium avium subsp. paratuberculosis in sheep and cattle. J Clin Microbiol 52:745–757. doi:10.1128/JCM.03233-13 24352996 PMC3957769

[16] Bustin SA, Benes V, Garson JA, Hellemans J, Huggett J, Kubista M, Mueller R, Nolan T, Pfaffl MW, Shipley GL, Vandesompele J, Wittwer CT. 2009. The MIQE guidelines: minimum information for publication of quantitative real-time PCR experiments. Clin Chem 55:611–622. doi:10.1373/clinchem.2008.112797 19246619

[17] Poupart P, Coene M, Van Heuverswyn H, Cocito C. 1993. Preparation of a specific RNA probe for detection of Mycobacterium paratuberculosis and diagnosis of Johne’s disease. J Clin Microbiol 31:1601–1605. doi:10.1128/jcm.31.6.1601-1605.1993 8315002 PMC265585

[18] Sidoti F, Banche G, Astegiano S, Allizond V, Cuffini AM, Bergallo M. 2011. Validation and standardization of IS900 and F57 real-time quantitative PCR assays for the specific detection and quantification of Mycobacterium avium subsp. paratuberculosis. Can J Microbiol 57:347–354. doi:10.1139/w11-022 21510779

[19] Conde C, Price-Carter M, Cochard T, Branger M, Stevenson K, Whittington R, Bannantine JP, Biet F. 2021. Whole-genome analysis of Mycobacterium avium subsp. paratuberculosis IS900 insertions reveals strain type-specific modalities. Front Microbiol 12. doi:10.3389/fmicb.2021.660002 PMC8141618 34040595

[20] Stabel JR, Bannantine JP. 2005. Development of a nested PCR method targeting a unique multicopy element, ISMap02, for detection of Mycobacterium avium subsp. paratuberculosis in fecal samples. J Clin Microbiol 43:4744–4750. doi:10.1128/JCM.43.9.4744-4750.2005 16145136 PMC1234153

[21] Bissonnette N, Brousseau JP, Ollier S, Byrne AS, Ibeagha-Awemu EM, Tahlan K. 2024. Systematic assessment of the reliability of quantitative PCR assays targeting IS900 for the detection of Mycobacterium avium ssp. paratuberculosis presence in animal and environmental samples. J Dairy Sci 107:7165–7184. doi:10.3168/jds.2023-24566 38754821

[22] Davis MW, Jorgensen EM. 2022. ApE, a plasmid editor: a freely available DNA manipulation and visualization program. Front Bioinform 2:818619. doi:10.3389/fbinf.2022.818619 36304290 PMC9580900

[23] Herthnek D, Englund S, Willemsen PTJ, Bölske G. 2006. Sensitive detection of Mycobacterium avium subsp. paratuberculosis in bovine semen by real-time PCR. J Appl Microbiol 100:1095–1102. doi:10.1111/j.1365-2672.2006.02924.x 16630010

[24] Slana I, Kralik P, Kralova A, Pavlik I. 2008. On-farm spread of Mycobacterium avium subsp. paratuberculosis in raw milk studied by IS900 and F57 competitive real time quantitative PCR and culture examination. Int J Food Microbiol 128:250–257. doi:10.1016/j.ijfoodmicro.2008.08.013 18824269

[25] Kim SG, Shin SJ, Jacobson RH, Miller LJ, Harpending PR, Stehman SM, Rossiter CA, Lein DA. 2002. Development and application of quantitative polymerase chain reaction assay based on the ABI 7700 system (TaqMan) for detection and quantification of Mycobacterium avium subsp. paratuberculosis. J Vet Diagn Invest 14:126–131. doi:10.1177/104063870201400206 11939333

[26] Irenge LM, Walravens K, Govaerts M, Godfroid J, Rosseels V, Huygen K, Gala JL. 2009. Development and validation of a triplex real-time PCR for rapid detection and specific identification of M. avium sub sp. paratuberculosis in faecal samples. Vet Microbiol 136:166–172. doi:10.1016/j.vetmic.2008.09.087 19095382

[27] Sevilla IA, Garrido JM, Molina E, Geijo MV, Elguezabal N, Vázquez P, Juste RA. 2014. Development and evaluation of a novel multicopy-element-targeting triplex PCR for detection of Mycobacterium avium subsp. paratuberculosis in feces. Appl Environ Microbiol 80:3757–3768. doi:10.1128/AEM.01026-14 24727272 PMC4054133

[28] Herthnek D, Bölske G. 2006. New PCR systems to confirm real-time PCR detection of Mycobacterium avium subsp. paratuberculosis. BMC Microbiol 6:87. doi:10.1186/1471-2180-6-87 17020599 PMC1609169

[29] Ricchi M, De Cicco C, Kralik P, Babak V, Boniotti MB, Savi R, Cerutti G, Cammi G, Garbarino C, Arrigoni N. 2014. Evaluation of viable Mycobacterium avium subsp. paratuberculosis in milk using peptide-mediated separation and Propidium Monoazide qPCR. FEMS Microbiol Lett 356:127–133. doi:10.1111/1574-6968.12480 24860938

[30] Byrne A, Ollier S, Tahlan K, Biet F, Bissonnette N. 2023. Genomic epidemiology of Mycobacterium avium subsp. paratuberculosis isolates from Canadian dairy herds provides evidence for multiple infection events. Front Genet 14:1043598. doi:10.3389/fgene.2023.1043598 36816022 PMC9934062

[31] Byrne A, Bissonnette N, Ollier S, Tahlan K. 2023. Investigating in vivo Mycobacterium avium subsp. paratuberculosis microevolution and mixed strain infections. Microbiol Spectr. doi:10.1128/spectrum.01716-23 PMC10581078 37584606

[32] Li L, Bannantine JP, Zhang Q, Amonsin A, May BJ, Alt D, Banerji N, Kanjilal S, Kapur V. 2005. The complete genome sequence of Mycobacterium avium subspecies paratuberculosis. Proc Natl Acad Sci USA 102:12344–12349. doi:10.1073/pnas.0505662102 16116077 PMC1194940

[33] Yun JJ, Heisler LE, Hwang IIL, Wilkins O, Lau SK, Hyrcza M, Jayabalasingham B, Jin J, McLaurin J, Tsao MS, Der SD. 2006. Genomic DNA functions as a universal external standard in quantitative real-time PCR. Nucleic Acids Res 34:e85. doi:10.1093/nar/gkl400 16840529 PMC1524913

[34] Donaghy JA, Johnston J, Rowe MT. 2011. Detection of Mycobacterium avium ssp. paratuberculosis in cheese, milk powder and milk using IS900 and f57-based qPCR assays. J Appl Microbiol 110:479–489. doi:10.1111/j.1365-2672.2010.04905.x 21155954

[35] Taylor S, Wakem M, Dijkman G, Alsarraj M, Nguyen M. 2010. A practical approach to RT-qPCR-Publishing data that conform to the MIQE guidelines. Methods 50:S1–S5. doi:10.1016/j.ymeth.2010.01.005 20215014

[36] Marete A, Ariel O, Ibeagha-Awemu E, Bissonnette N. 2021. Identification of long non-coding RNA isolated from naturally infected macrophages and associated with bovine Johne's disease in canadian holstein using a combination of neural networks and logistic regression. Front Vet Sci 8:639053. doi:10.3389/fvets.2021.639053 33969037 PMC8100051

[37] Whittington RJ, Begg DJ, de Silva K, Purdie AC, Dhand NK, Plain KM. 2017. Case definition terminology for paratuberculosis (Johne’s disease). BMC Vet Res 13:328. doi:10.1186/s12917-017-1254-6 29121939 PMC5680782

[38] Arango-Sabogal JC, Côté G, Paré J, Labrecque O, Roy J-P, Buczinski S, Doré E, Fairbrother JH, Bissonnette N, Wellemans V, Fecteau G. 2016. Detection of Mycobacterium avium subspecies paratuberculosis in tie-stall dairy herds using a standardized environmental sampling technique and targeted pooled samples. Can J Vet Res 80:175–182. 27408329 PMC4924550

[39] Mizzi R, Plain KM, Whittington R, Timms VJ. 2022. Global phylogeny of Mycobacterium avium and identification of mutation hotspots during niche adaptation. Front Microbiol 13:892333. doi:10.3389/fmicb.2022.892333 35602010 PMC9121174

[40] Schönenbrücher H, Abdulmawjood A, Failing K, Bülte M. 2008. New triplex real-time PCR assay for detection of Mycobacterium avium subsp. paratuberculosis in bovine feces. Appl Environ Microbiol 74:2751–2758. doi:10.1128/AEM.02534-07 18326682 PMC2394907

[41] Singh U, Arutyunov D, Basu U, Santos Seckler HD, Szymanski CM, Evoy S. 2014. Mycobacteriophage lysin-mediated capture of cells for the PCR detection of Mycobacterium avium subspecies paratuberculosis. Anal. Methods 6:5682–5689. doi:10.1039/C4AY01072H

[42] Bustin SA, Mueller R, Nolan T. 2020. Parameters for successful PCR primer design. Methods Mol Biol 2065:5–22. doi:10.1007/978-1-4939-9833-3_2 31578684

[43] Slana I, Liapi M, Moravkova M, Kralova A, Pavlik I. 2009. Mycobacterium avium subsp. paratuberculosis in cow bulk tank milk in Cyprus detected by culture and quantitative IS900 and F57 real-time PCR. Prev Vet Med 89:223–226. doi:10.1016/j.prevetmed.2009.02.020 19349086

[44] Nielsen SS, Toft N, Gardner IA. 2011. Structured approach to design of diagnostic test evaluation studies for chronic progressive infections in animals. Vet Microbiol 150:115–125. doi:10.1016/j.vetmic.2011.01.019 21333467

[45] Acharya KR, Dhand NK, Whittington RJ, Plain KM. 2017. PCR inhibition of a quantitative PCR for detection of Mycobacterium avium subspecies paratuberculosis DNA in feces: diagnostic implications and potential solutions. Front Microbiol 8:115. doi:10.3389/fmicb.2017.00115 28210245 PMC5288348

[46] Park HT, Shin MK, Park HE, Cho YI, Yoo HS. 2016. PCR-based detection of Mycobacterium avium subsp. paratuberculosis infection in cattle in South Korea using fecal samples. J Vet Med Sci 78:1537–1540. doi:10.1292/jvms.15-0271 27301582 PMC5059387

[47] Tasara T, Hoelzle LE, Stephan R. 2005. Development and evaluation of a Mycobacterium avium subspecies paratuberculosis (MAP) specific multiplex PCR assay. Int J Food Microbiol 104:279–287. doi:10.1016/j.ijfoodmicro.2005.03.009 15982769

[48] Cousins DV, Whittington R, Marsh I, Masters A, Evans RJ, Kluver P. 1999. Mycobacteria distenct from Mycobacterium avium subsp. paratuberculosis isolated from the faeces of ruminants possess IS900-like sequences detectable IS900 polymerase chain reaction: implications for diagnosis. Mol Cell Probes 13:431–442. doi:10.1006/mcpr.1999.0275 10657148

[49] Bull TJ, McMinn EJ, Sidi-Boumedine K, Skull A, Durkin D, Neild P, Rhodes G, Pickup R, Hermon-Taylor J. 2003. Detection and verification of Mycobacterium avium subsp. paratuberculosis in fresh ileocolonic mucosal biopsy specimens from individuals with and without Crohn’s disease. J Clin Microbiol 41:2915–2923. doi:10.1128/JCM.41.7.2915-2923.2003 12843021 PMC165291

