# Supplementary material for: Systematic evaluation and optimization of TaqMan qPCR assays targeting F57, ISMAP02, and IS900 for multiplex detection of Mycobacterium avium subsp. paratuberculosis
Source: J Clin Microbiol. 2025 Dec 29;64(2):e00872-25. doi: 10.1128/jcm.00872-25 (PMC12892987; doi:10.1128/jcm.00872-25)
Supplement: Figure S2 — Graphs of the qPCR results. All samples (cows and feces) were analyzed using the 18 multiplex-qPDR assays. [file jcm.00872-25-s0002.pdf]

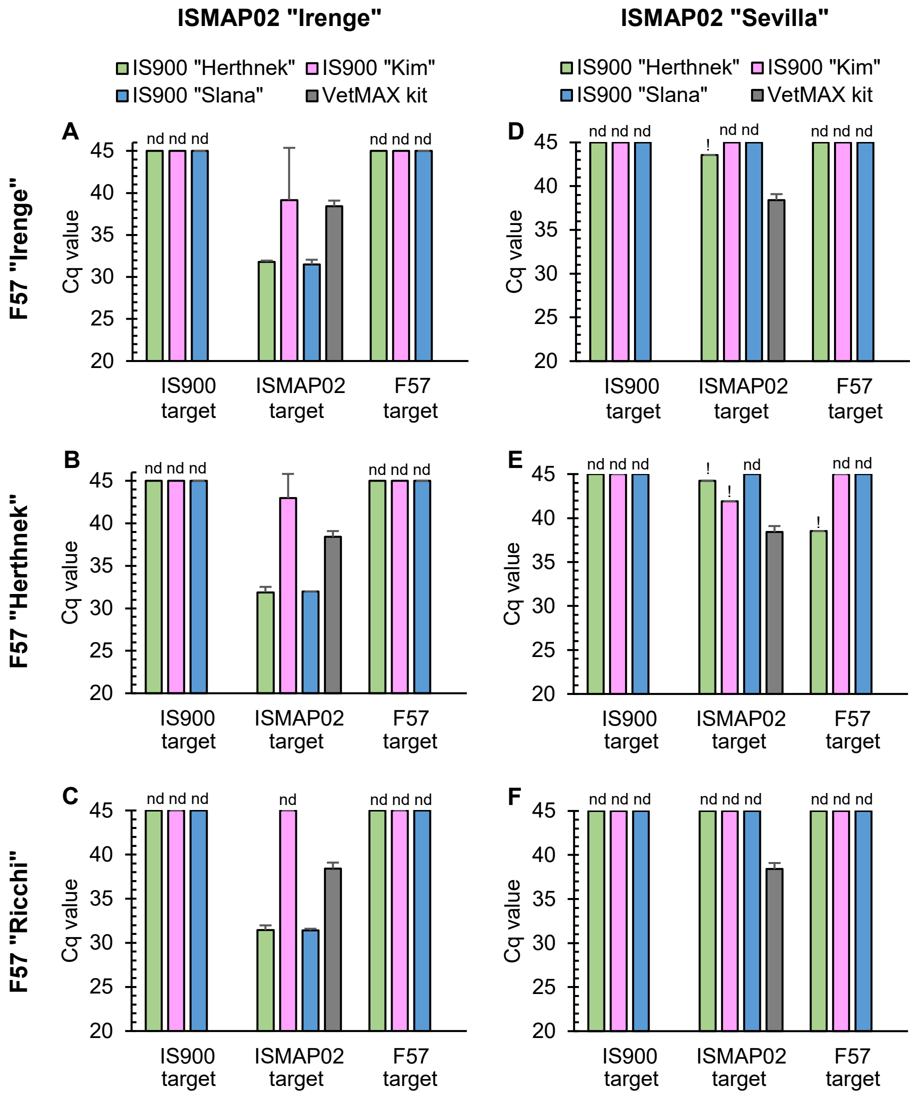


**Sample F05** (low)

**Supplemental Fig S2a.** Results of the 18 multiplex qPCR assays for MAP detection in fecal sample F05.

The results are organized into six panels based on the composition of each multiplex assay:
Left panels: Nine assays incorporating the ISMAP*02*-Irenge design.
Right panels: Nine assays incorporating the ISMAP*02*-Sevilla design.

Each row of panels corresponds to a different F*57* target design:
Top row: F*57*-Irenge
Middle row: F*57*-Herthnek
Bottom row: F*57*-Ricchi

The IS900 target design used in each multiplex is indicated by the color of the bars:
Green: IS*900*-Herthnek
Pink: IS*900*-Kim
Blue: IS*900*-Slana

n.d., both replicates were not detected; !, only one replicate was detected.

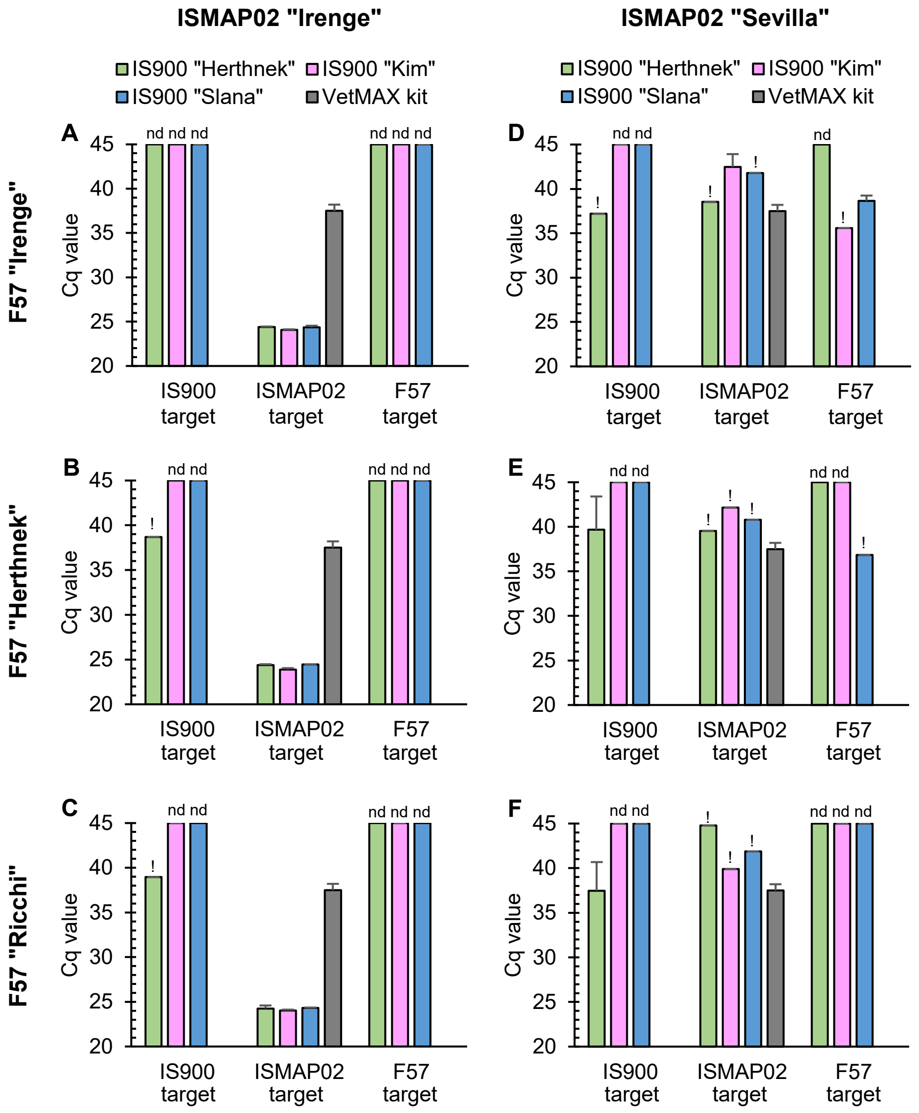


**Sample F06** (low)

**Supplemental Fig S2b.** Results of the 18 multiplex qPCR assays for MAP detection in fecal sample F06.

The results are organized into six panels based on the composition of each multiplex assay:
Left panels: Nine assays incorporating the ISMAP*02*-Irenge design.
Right panels: Nine assays incorporating the ISMAP*02*-Sevilla design.

Each row of panels corresponds to a different F*57* target design:
Top row: F*57*-Irenge
Middle row: F*57*-Herthnek
Bottom row: F*57*-Ricchi

The IS900 target design used in each multiplex is indicated by the color of the bars:
Green: IS*900*-Herthnek
Pink: IS*900*-Kim
Blue: IS*900*-Slana

n.d., both replicates were not detected; !, only one replicate was detected.

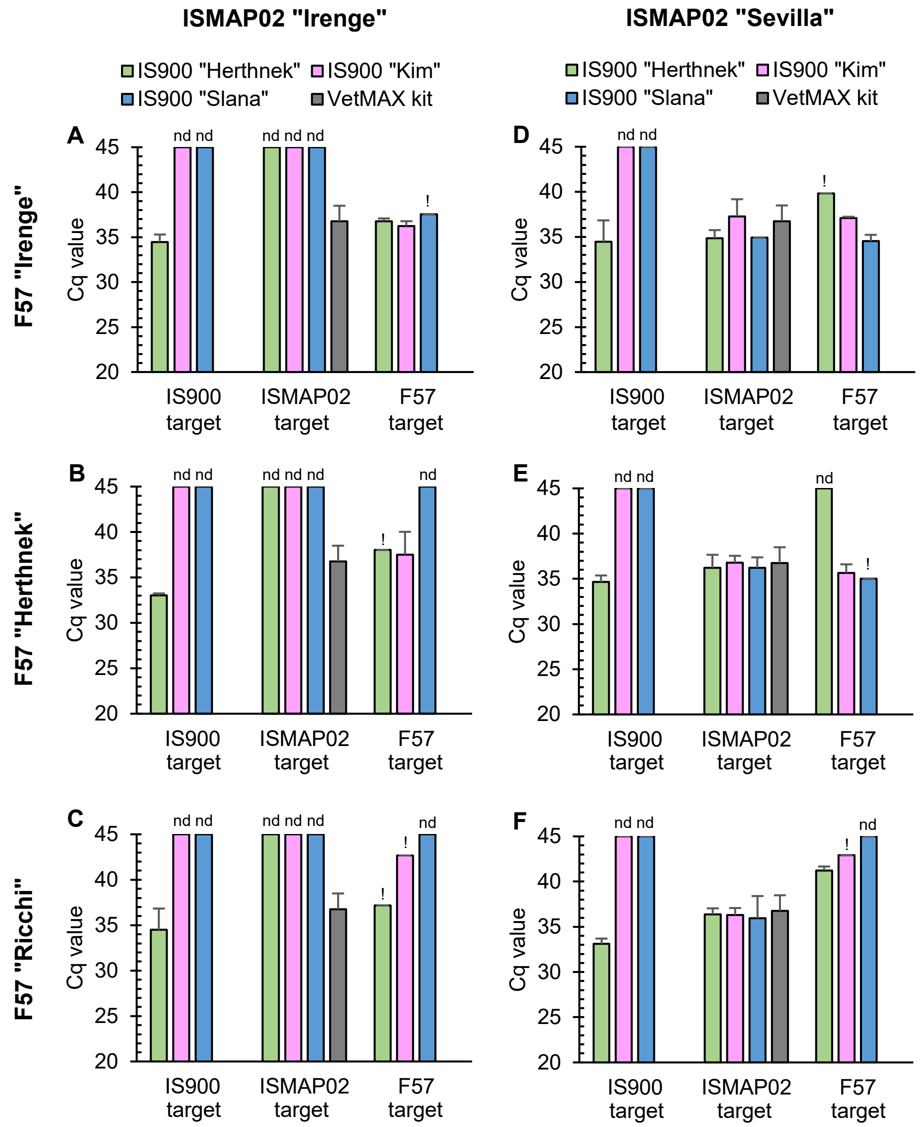


**Sample F07** (low)

**Supplemental Fig S2c.** Results of the 18 multiplex qPCR assays for MAP detection in fecal sample F07.

The results are organized into six panels based on the composition of each multiplex assay:
Left panels: Nine assays incorporating the ISMAP*02*-Irenge design.
Right panels: Nine assays incorporating the ISMAP*02*-Sevilla design.

Each row of panels corresponds to a different F*57* target design:
Top row: F*57*-Irenge
Middle row: F*57*-Herthnek
Bottom row: F*57*-Ricchi

The IS900 target design used in each multiplex is indicated by the color of the bars:
Green: IS*900*-Herthnek
Pink: IS*900*-Kim
Blue: IS*900*-Slana

n.d., both replicates were not detected; !, only one replicate was detected.

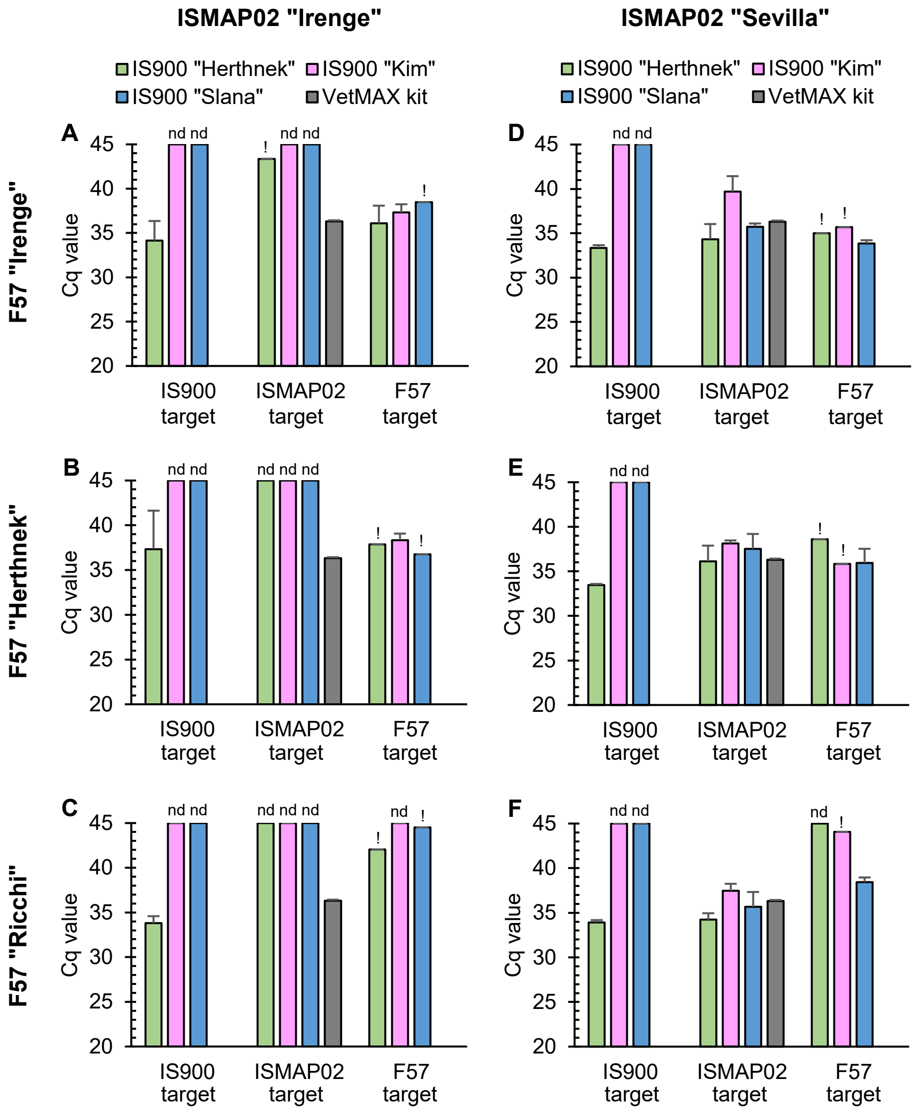


**Sample F08** (low)

**Supplemental Fig S2d.** Results of the 18 multiplex qPCR assays for MAP detection in fecal sample F08.

The results are organized into six panels based on the composition of each multiplex assay:
Left panels: Nine assays incorporating the ISMAP*02*-Irenge design.
Right panels: Nine assays incorporating the ISMAP*02*-Sevilla design.

Each row of panels corresponds to a different F*57* target design:
Top row: F*57*-Irenge
Middle row: F*57*-Herthnek
Bottom row: F*57*-Ricchi

The IS900 target design used in each multiplex is indicated by the color of the bars:
Green: IS*900*-Herthnek
Pink: IS*900*-Kim
Blue: IS*900*-Slana

n.d., both replicates were not detected; !, only one replicate was detected.

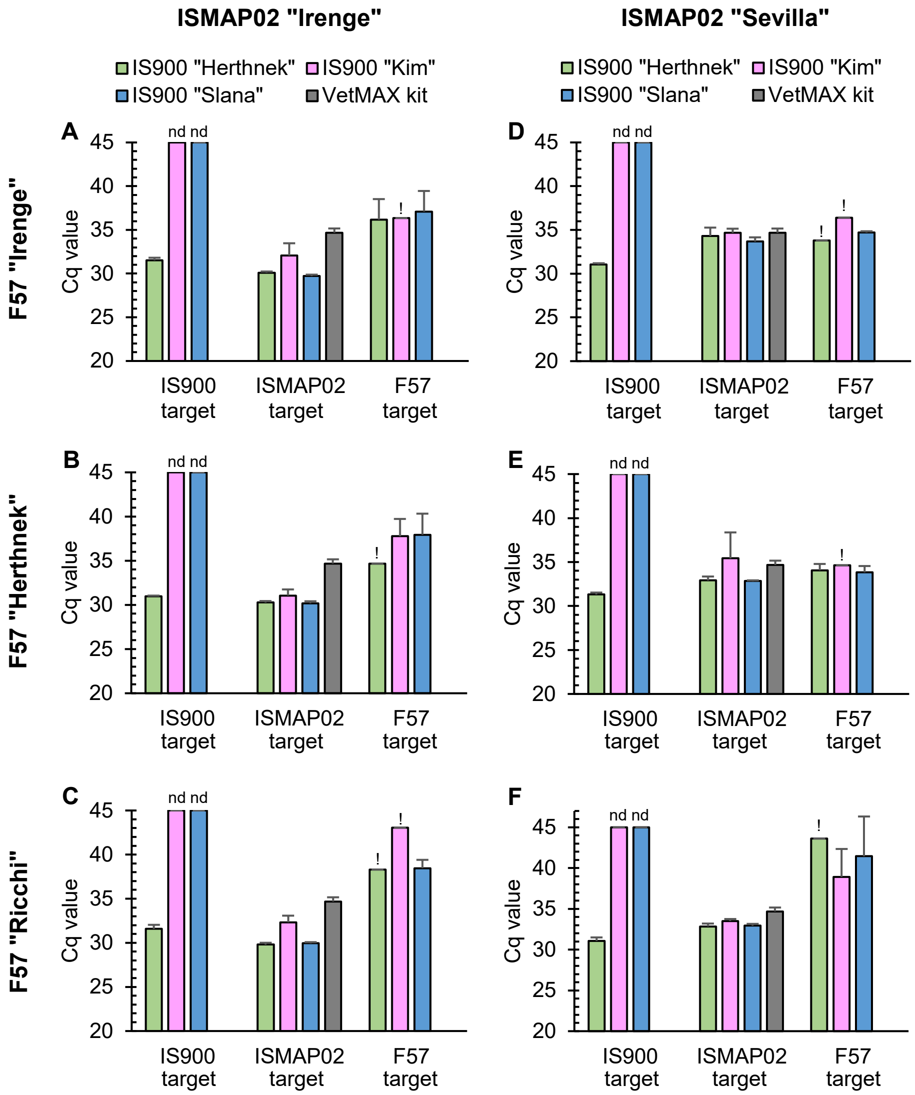


**Sample F09** (low)

**Supplemental Fig S2e.** Results of the 18 multiplex qPCR assays for MAP detection in fecal sample F09.

The results are organized into six panels based on the composition of each multiplex assay:
Left panels: Nine assays incorporating the ISMAP*02*-Irenge design.
Right panels: Nine assays incorporating the ISMAP*02*-Sevilla design.

Each row of panels corresponds to a different F*57* target design:
Top row: F*57*-Irenge
Middle row: F*57*-Herthnek
Bottom row: F*57*-Ricchi

The IS900 target design used in each multiplex is indicated by the color of the bars:
Green: IS*900*-Herthnek
Pink: IS*900*-Kim
Blue: IS*900*-Slana

n.d., both replicates were not detected; !, only one replicate was detected.

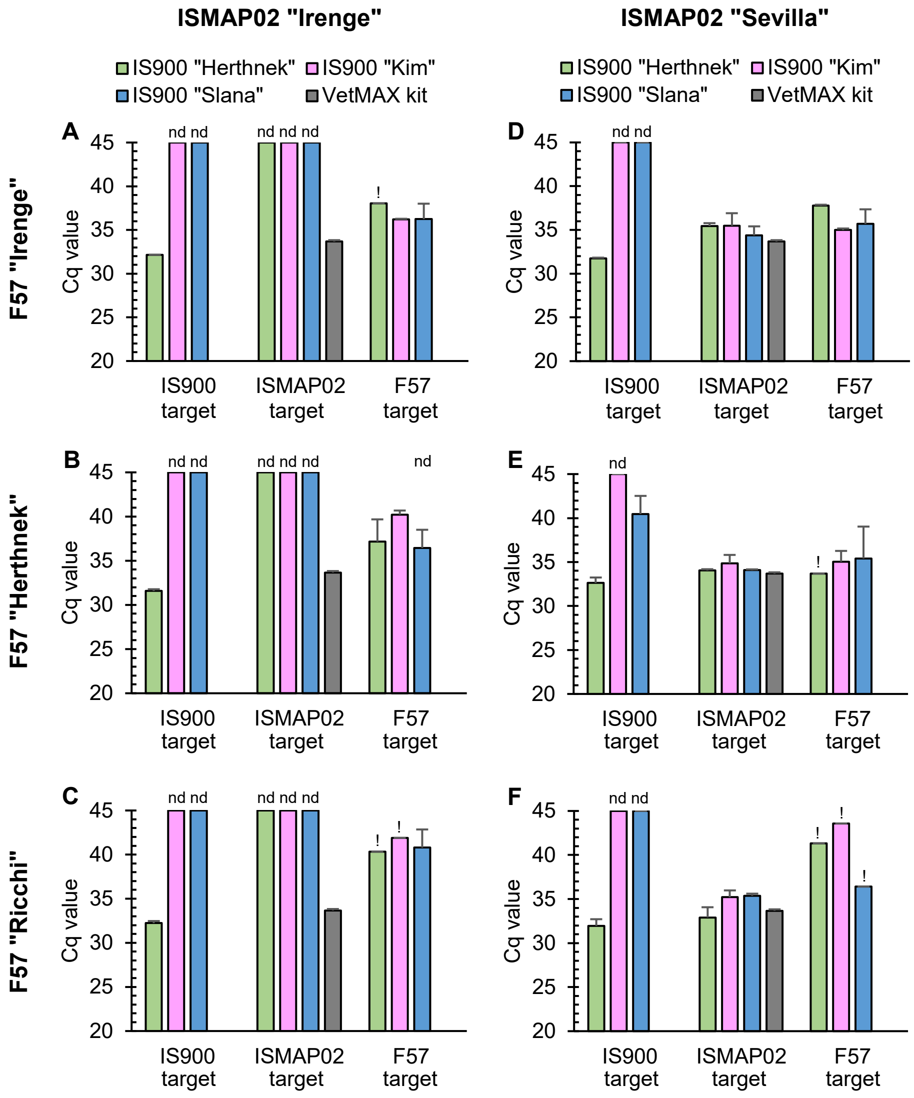


**Sample F10** (low)

**Supplemental Fig S2f.** Results of the 18 multiplex qPCR assays for MAP detection in fecal sample F10.

The results are organized into six panels based on the composition of each multiplex assay:
Left panels: Nine assays incorporating the ISMAP*02*-Irenge design.
Right panels: Nine assays incorporating the ISMAP*02*-Sevilla design.

Each row of panels corresponds to a different F*57* target design:
Top row: F*57*-Irenge
Middle row: F*57*-Herthnek
Bottom row: F*57*-Ricchi

The IS900 target design used in each multiplex is indicated by the color of the bars:
Green: IS*900*-Herthnek
Pink: IS*900*-Kim
Blue: IS*900*-Slana

n.d., both replicates were not detected; !, only one replicate was detected.

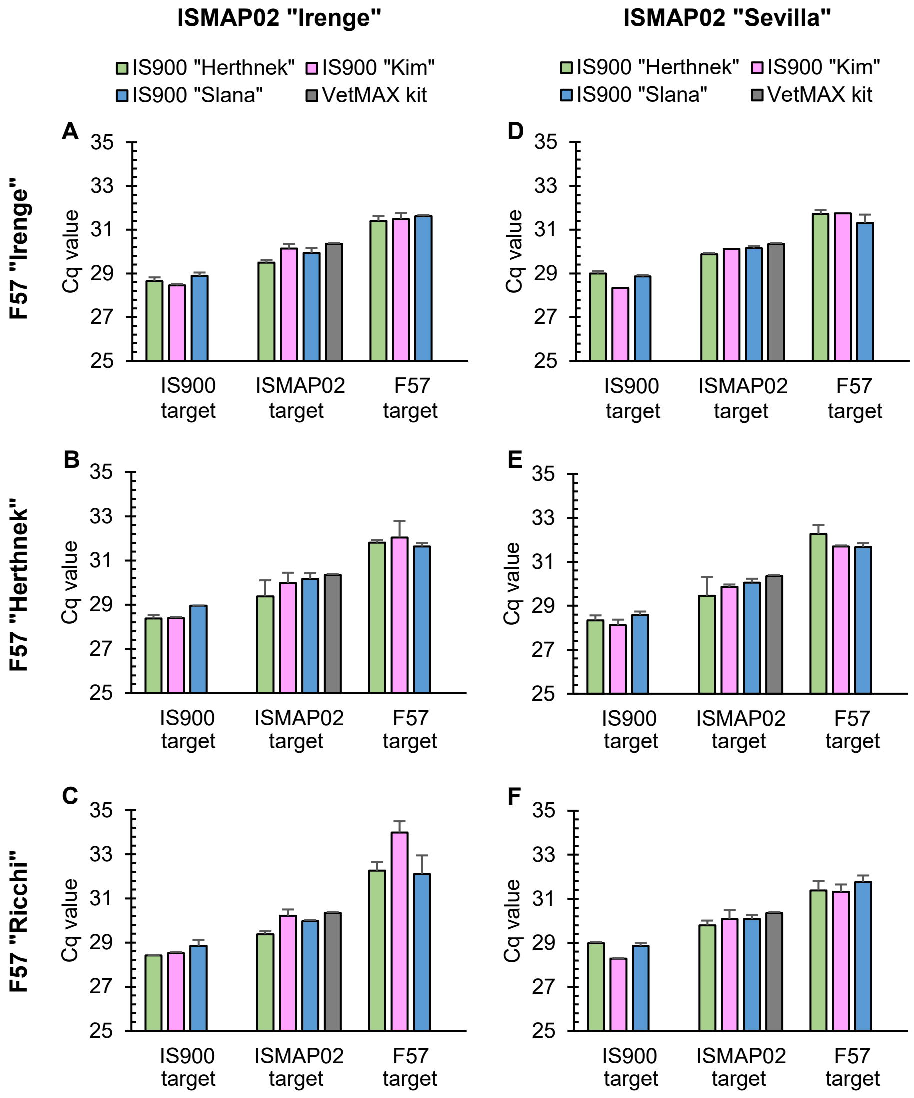


**Sample F11** (moderate)

**Supplemental Fig S2g**. Results of the 18 multiplex qPCR assays for MAP detection in fecal sample F11.

The results are organized into six panels based on the composition of each multiplex assay:
Left panels: Nine assays incorporating the ISMAP*02*-Irenge design.
Right panels: Nine assays incorporating the ISMAP*02*-Sevilla design.

Each row of panels corresponds to a different F*57* target design:
Top row: F*57*-Irenge
Middle row: F*57*-Herthnek
Bottom row: F*57*-Ricchi

The IS900 target design used in each multiplex is indicated by the color of the bars:
Green: IS*900*-Herthnek
Pink: IS*900*-Kim
Blue: IS*900*-Slana

n.d., both replicates were not detected; !, only one replicate was detected.

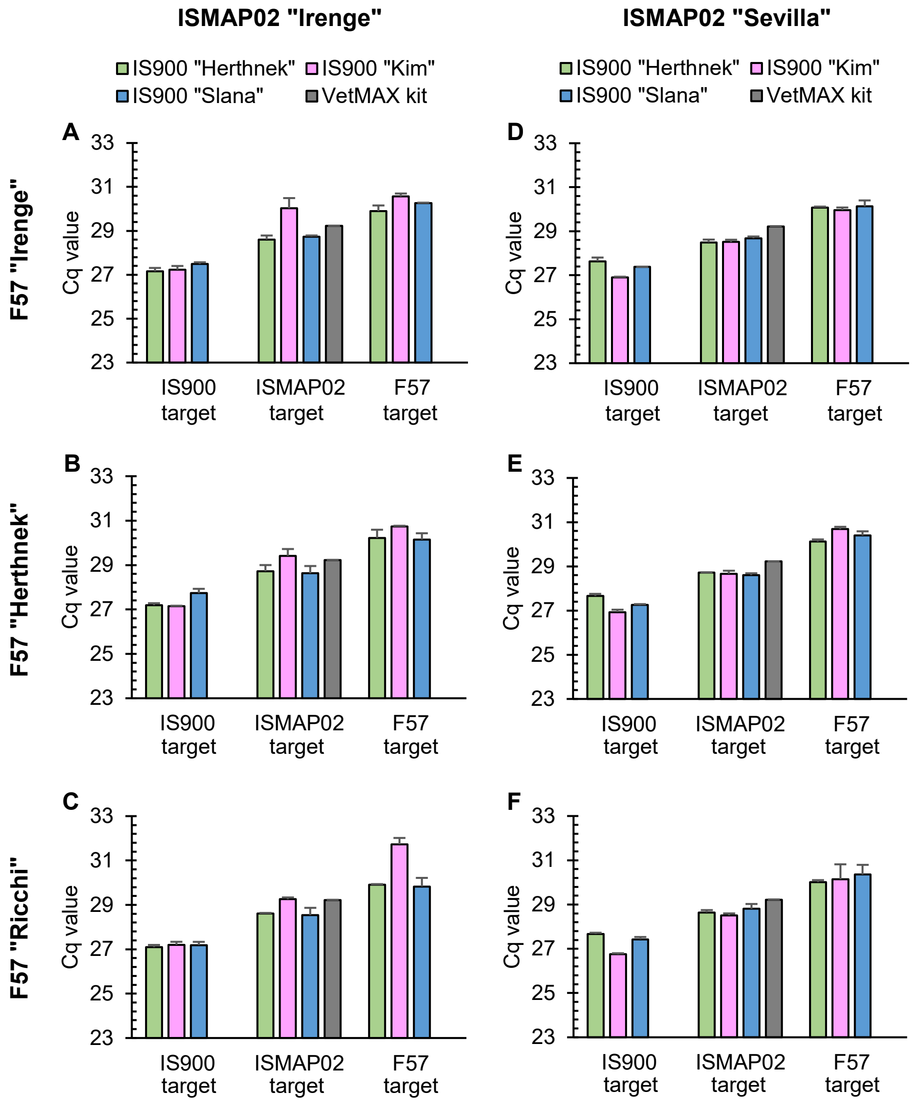


**Sample F12** (moderate)

**Supplemental Fig S2h**. Results of the 18 multiplex qPCR assays for MAP detection in fecal sample F12.

The results are organized into six panels based on the composition of each multiplex assay:
- Left panels: Nine assays incorporating the ISMAP*02*-Irenge design.
- Right panels: Nine assays incorporating the ISMAP*02*-Sevilla design.

Each row of panels corresponds to a different F*57* target design:
- Top row: F*57*-Irenge
- Middle row: F*57*-Herthnek
- Bottom row: F*57*-Ricchi

The IS900 target design used in each multiplex is indicated by the color of the bars:
- Green: IS*900*-Herthnek
- Pink: IS*900*-Kim
- Blue: IS*900*-Slana

n.d., both replicates were not detected; !, only one replicate was detected.

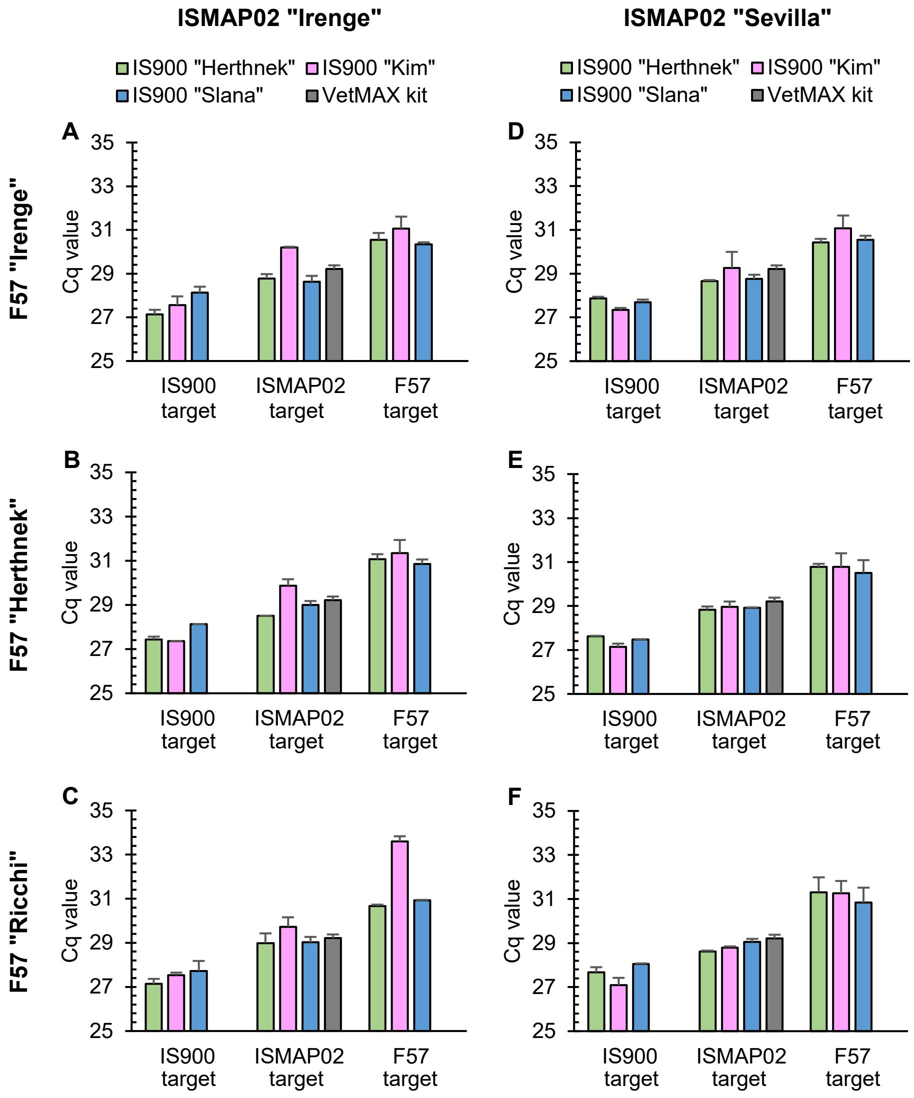


**Sample F13** (moderate)

**Supplemental Fig S2i**. Results of the 18 multiplex qPCR assays for MAP detection in fecal sample F13.

The results are organized into six panels based on the composition of each multiplex assay:
Left panels: Nine assays incorporating the ISMAP*02*-Irenge design.
Right panels: Nine assays incorporating the ISMAP*02*-Sevilla design.

Each row of panels corresponds to a different F*57* target design:
Top row: F*57*-Irenge
Middle row: F*57*-Herthnek
Bottom row: F*57*-Ricchi

The IS900 target design used in each multiplex is indicated by the color of the bars:
Green: IS*900*-Herthnek
Pink: IS*900*-Kim
Blue: IS*900*-Slana

n.d., both replicates were not detected; !, only one replicate was detected.

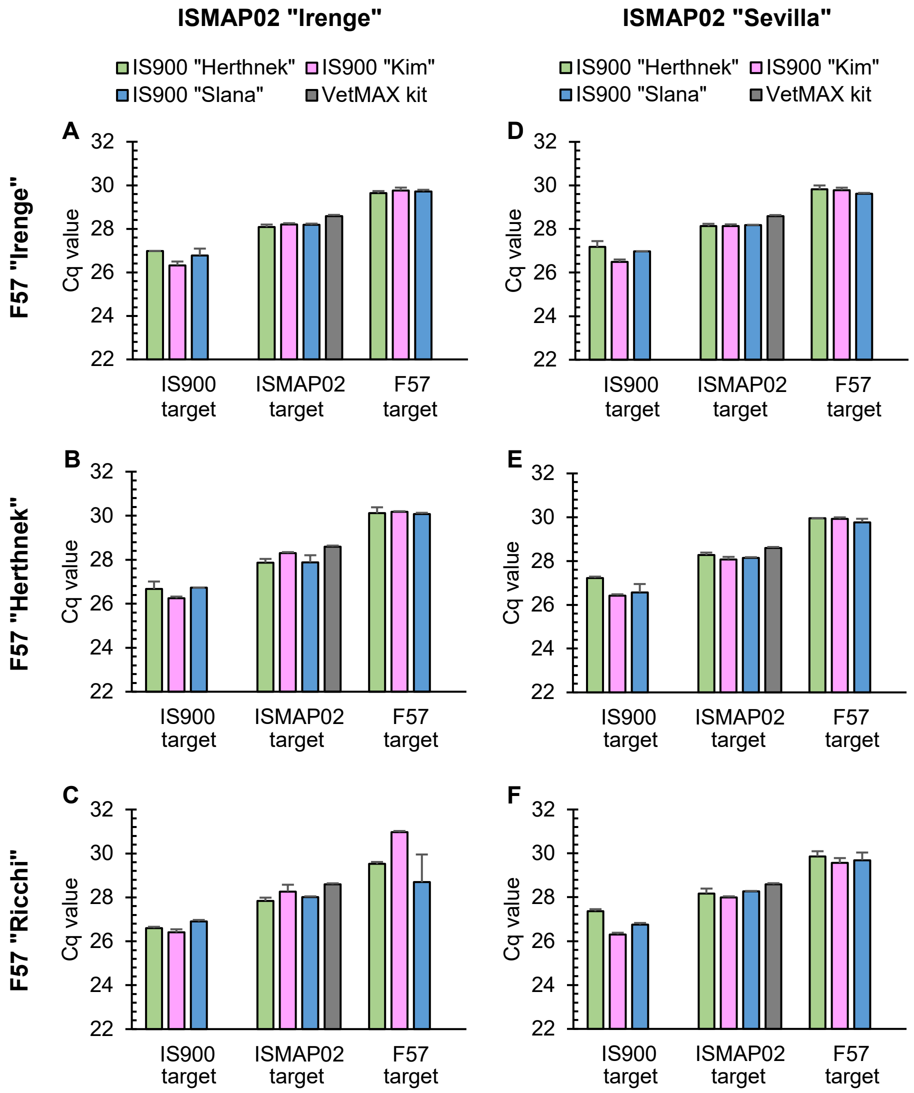


**Sample F14** (moderate)

**Supplemental Fig S2j**. Results of the 18 multiplex qPCR assays for MAP detection in fecal sample F14.

The results are organized into six panels based on the composition of each multiplex assay:
Left panels: Nine assays incorporating the ISMAP*02*-Irenge design.
Right panels: Nine assays incorporating the ISMAP*02*-Sevilla design.

Each row of panels corresponds to a different F*57* target design:
Top row: F*57*-Irenge
Middle row: F*57*-Herthnek
Bottom row: F*57*-Ricchi

The IS900 target design used in each multiplex is indicated by the color of the bars:
Green: IS*900*-Herthnek
Pink: IS*900*-Kim
Blue: IS*900*-Slana

n.d., both replicates were not detected; !, only one replicate was detected.

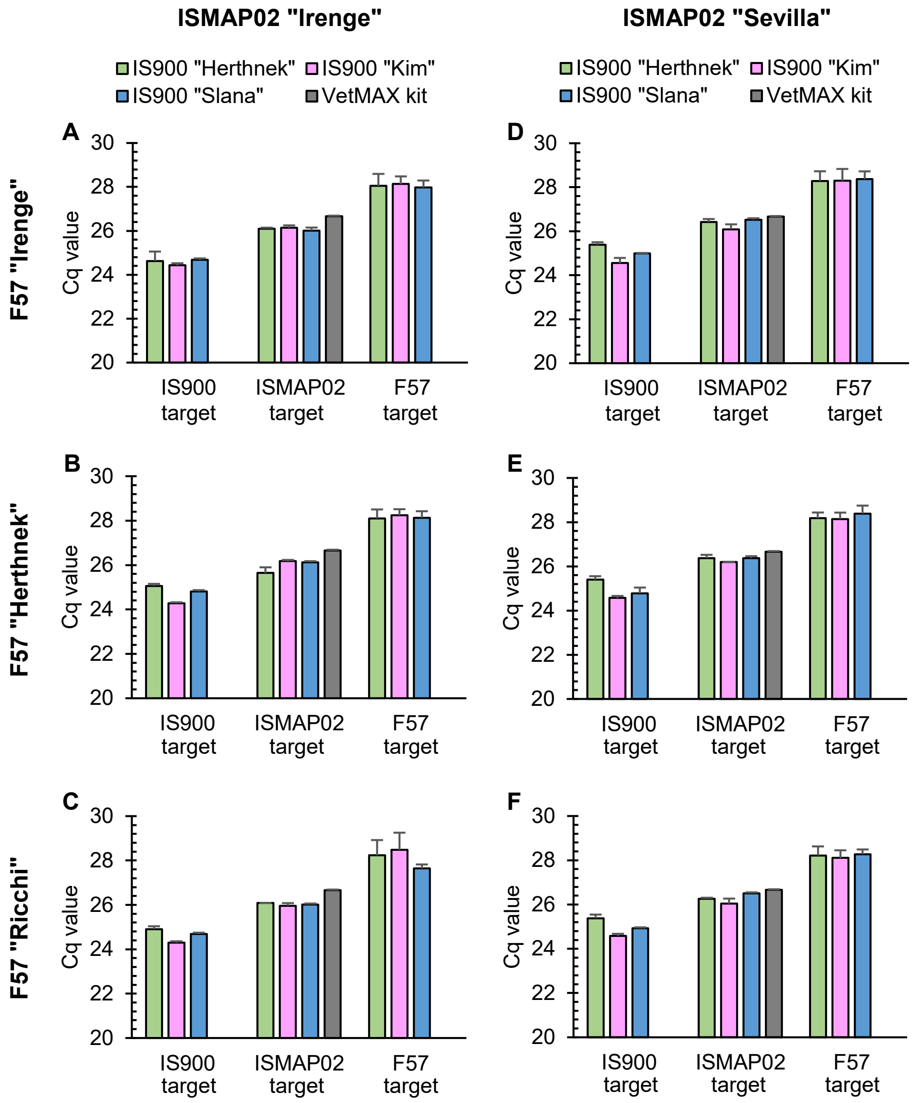


**Sample F15** (moderate)

**Supplemental Fig S2k**. Results of the 18 multiplex qPCR assays for MAP detection in fecal sample F15.

The results are organized into six panels based on the composition of each multiplex assay:
Left panels: Nine assays incorporating the ISMAP*02*-Irenge design.
Right panels: Nine assays incorporating the ISMAP*02*-Sevilla design.

Each row of panels corresponds to a different F*57* target design:
Top row: F*57*-Irenge
Middle row: F*57*-Herthnek
Bottom row: F*57*-Ricchi

The IS900 target design used in each multiplex is indicated by the color of the bars:
Green: IS*900*-Herthnek
Pink: IS*900*-Kim
Blue: IS*900*-Slana

n.d., both replicates were not detected; !, only one replicate was detected.

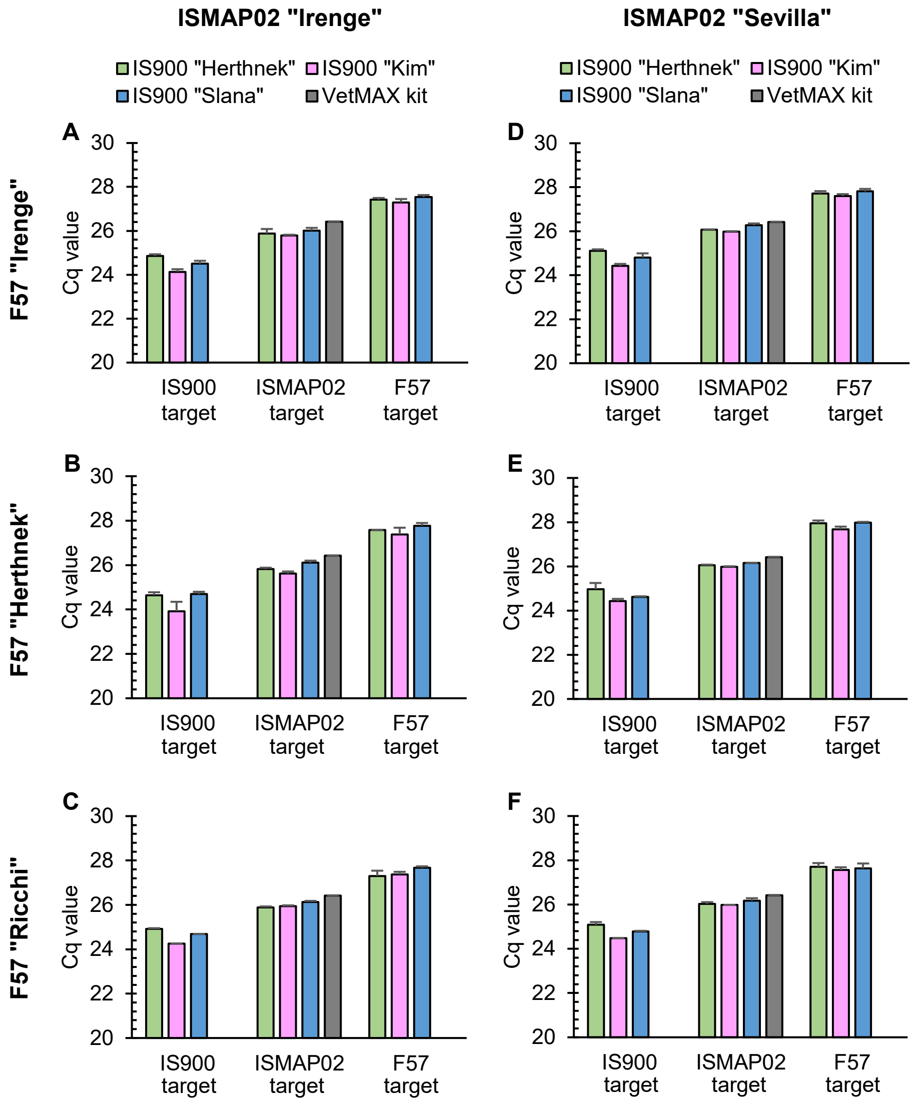


**Sample F16** (moderate)

**Supplemental Fig S2l**. Results of the 18 multiplex qPCR assays for MAP detection in fecal sample F16.

The results are organized into six panels based on the composition of each multiplex assay:
Left panels: Nine assays incorporating the ISMAP*02*-Irenge design.
Right panels: Nine assays incorporating the ISMAP*02*-Sevilla design.

Each row of panels corresponds to a different F*57* target design:
Top row: F*57*-Irenge
Middle row: F*57*-Herthnek
Bottom row: F*57*-Ricchi

The IS900 target design used in each multiplex is indicated by the color of the bars:
Green: IS*900*-Herthnek
Pink: IS*900*-Kim
Blue: IS*900*-Slana

n.d., both replicates were not detected; !, only one replicate was detected.

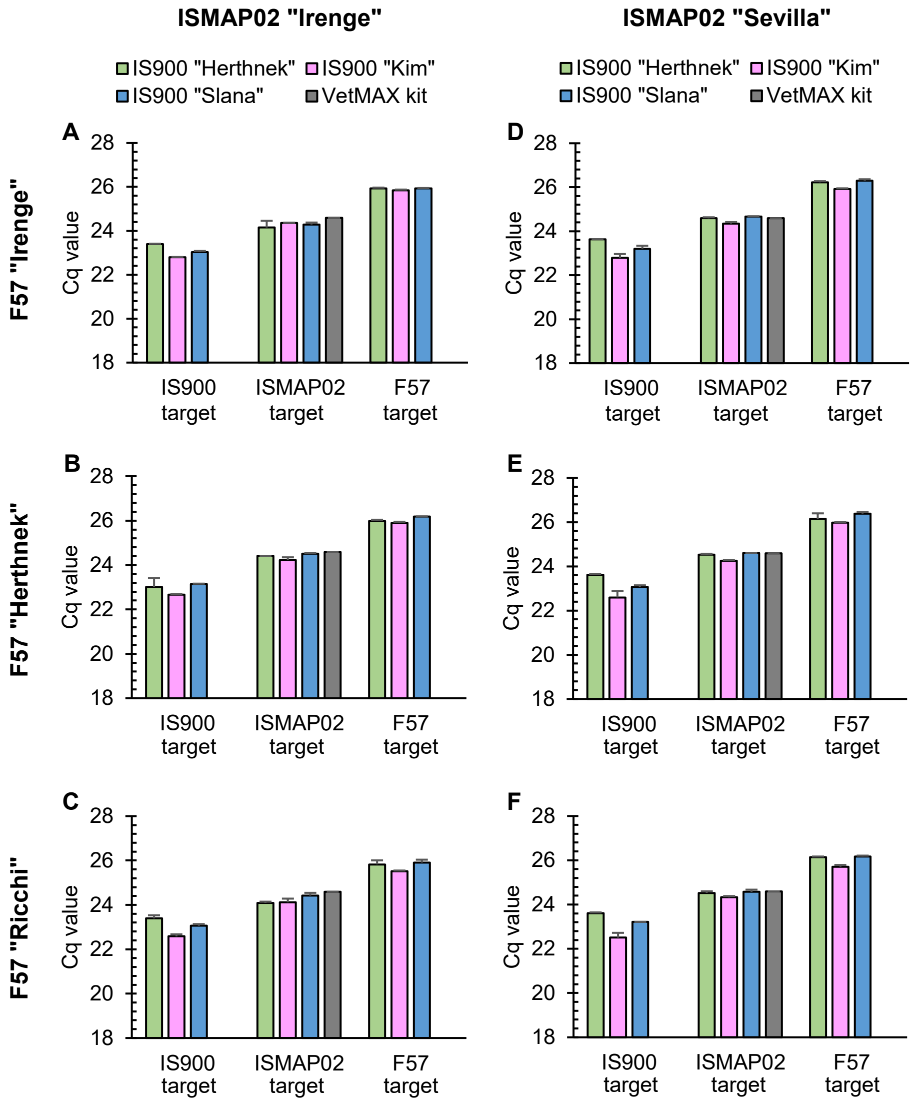


**Sample F17** (high)

**Supplemental Fig S2m**. Results of the 18 multiplex qPCR assays for MAP detection in fecal sample F17.

The results are organized into six panels based on the composition of each multiplex assay:
Left panels: Nine assays incorporating the ISMAP*02*-Irenge design.
Right panels: Nine assays incorporating the ISMAP*02*-Sevilla design.

Each row of panels corresponds to a different F*57* target design:
Top row: F*57*-Irenge
Middle row: F*57*-Herthnek
Bottom row: F*57*-Ricchi

The IS900 target design used in each multiplex is indicated by the color of the bars:
Green: IS*900*-Herthnek
Pink: IS*900*-Kim
Blue: IS*900*-Slana

n.d., both replicates were not detected; !, only one replicate was detected.

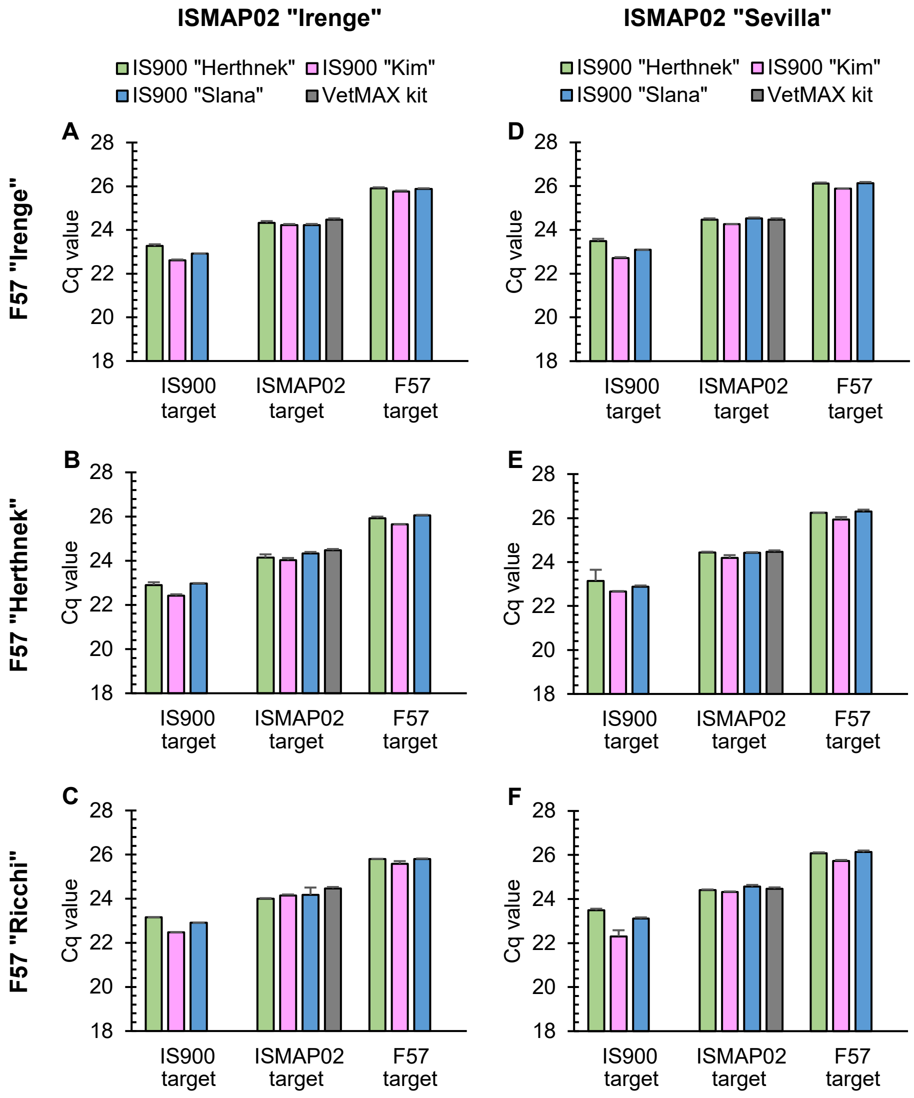


**Sample F18** (high)

**Supplemental Fig S2n**. Results of the 18 multiplex qPCR assays for MAP detection in fecal sample F18.

The results are organized into six panels based on the composition of each multiplex assay:
Left panels: Nine assays incorporating the ISMAP*02*-Irenge design.
Right panels: Nine assays incorporating the ISMAP*02*-Sevilla design.

Each row of panels corresponds to a different F*57* target design:
Top row: F*57*-Irenge
Middle row: F*57*-Herthnek
Bottom row: F*57*-Ricchi

The IS900 target design used in each multiplex is indicated by the color of the bars:
Green: IS*900*-Herthnek
Pink: IS*900*-Kim
Blue: IS*900*-Slana

n.d., both replicates were not detected; !, only one replicate was detected.

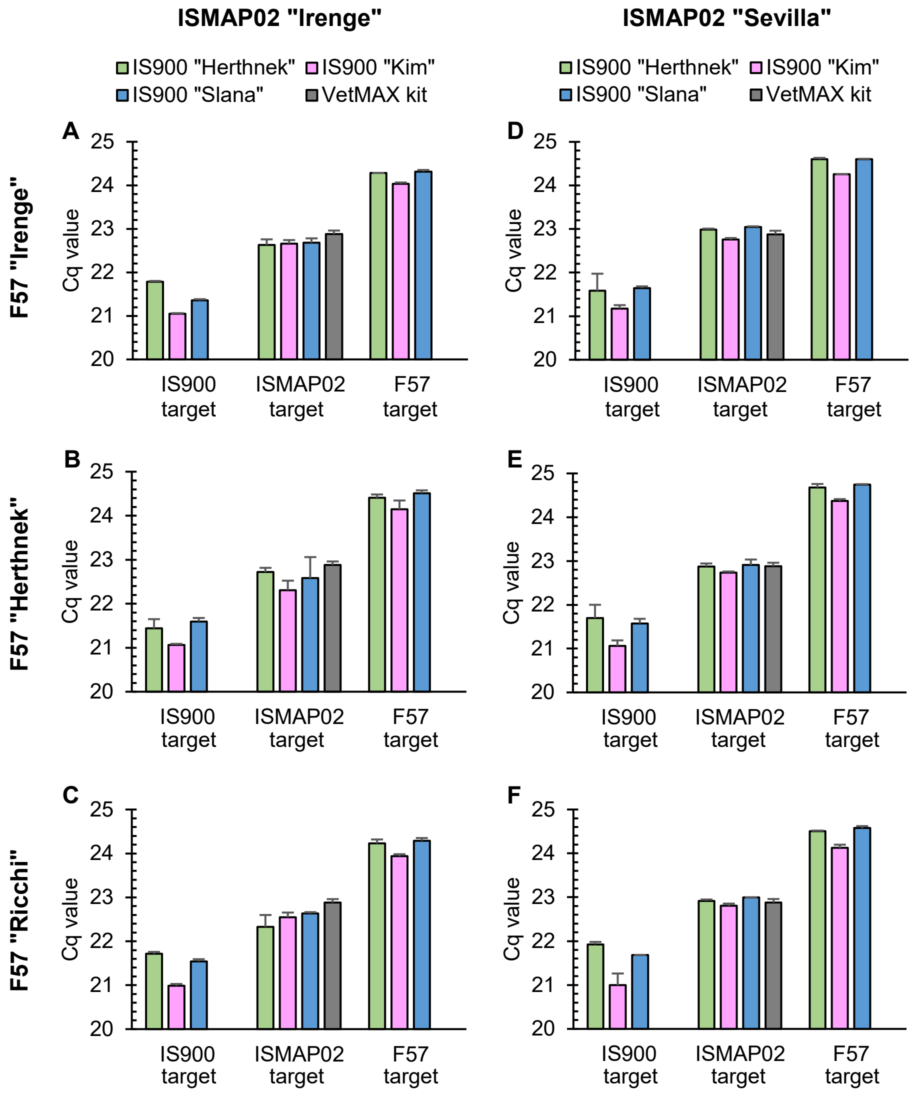


**Sample F19** (high)

**Supplemental Fig S2o**. Results of the 18 multiplex qPCR assays for MAP detection in fecal sample F19.

The results are organized into six panels based on the composition of each multiplex assay:
Left panels: Nine assays incorporating the ISMAP*02*-Irenge design.
Right panels: Nine assays incorporating the ISMAP*02*-Sevilla design.

Each row of panels corresponds to a different F*57* target design:
Top row: F*57*-Irenge
Middle row: F*57*-Herthnek
Bottom row: F*57*-Ricchi

The IS900 target design used in each multiplex is indicated by the color of the bars:
Green: IS*900*-Herthnek
Pink: IS*900*-Kim
Blue: IS*900*-Slana

n.d., both replicates were not detected; !, only one replicate was detected.

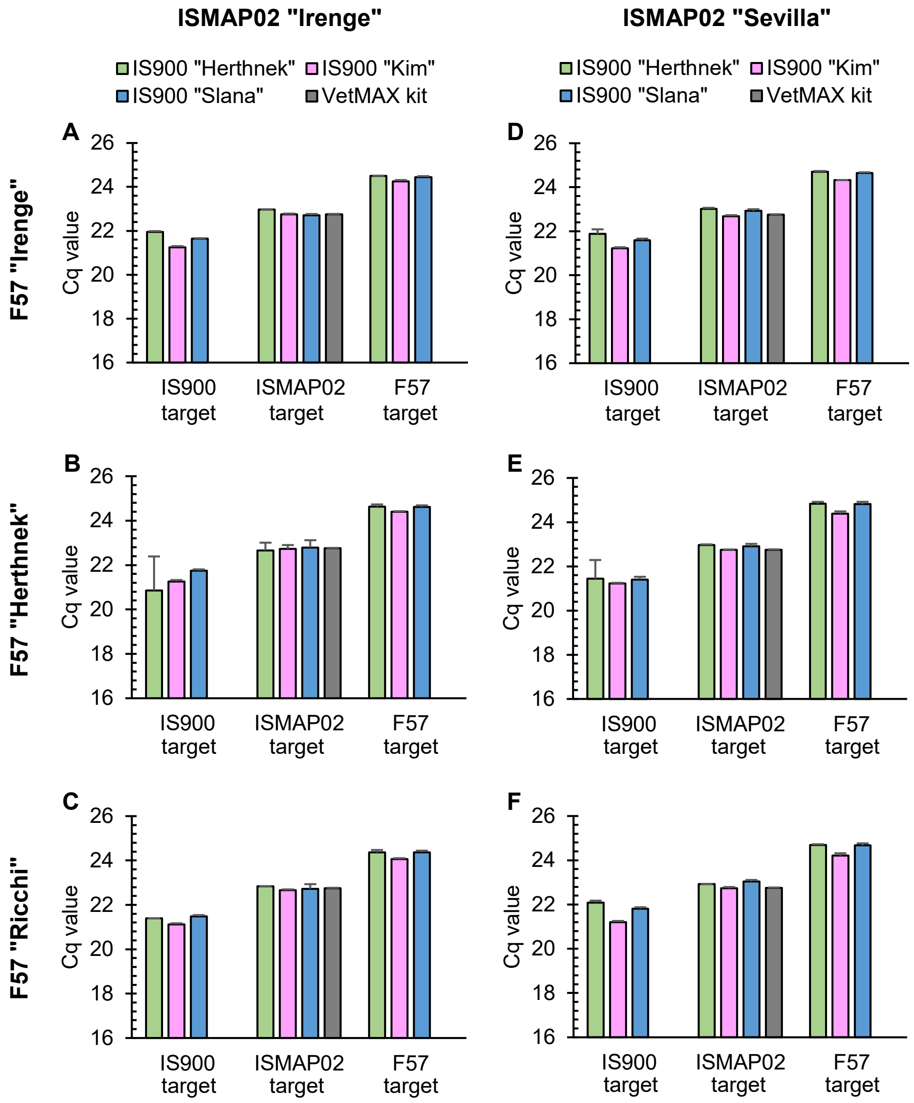


**Sample F20** (high)

**Supplemental Fig S2p**. Results of the 18 multiplex qPCR assays for MAP detection in fecal sample F20.

The results are organized into six panels based on the composition of each multiplex assay:
Left panels: Nine assays incorporating the ISMAP*02*-Irenge design.
Right panels: Nine assays incorporating the ISMAP*02*-Sevilla design.

Each row of panels corresponds to a different F*57* target design:
Top row: F*57*-Irenge
Middle row: F*57*-Herthnek
Bottom row: F*57*-Ricchi

The IS900 target design used in each multiplex is indicated by the color of the bars:
Green: IS*900*-Herthnek
Pink: IS*900*-Kim
Blue: IS*900*-Slana

n.d., both replicates were not detected; !, only one replicate was detected.

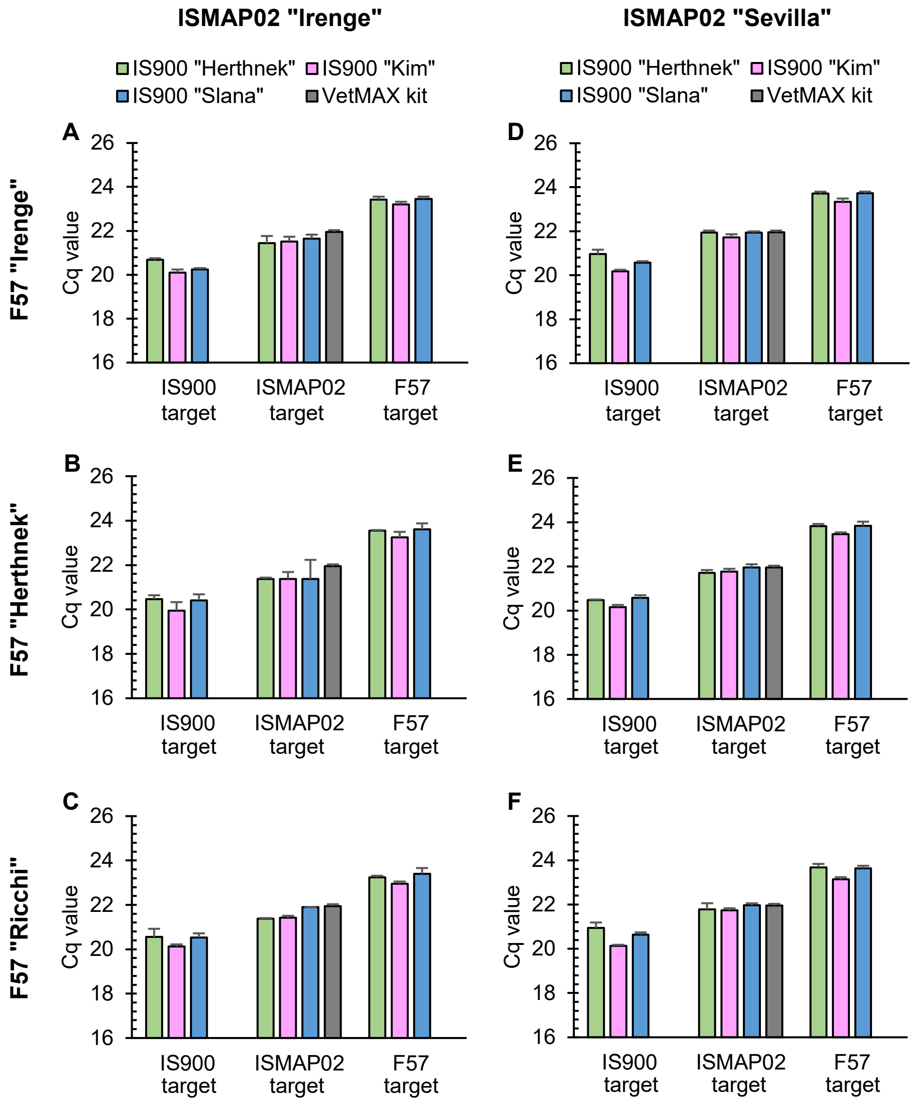


**Sample F21** (high)

**Supplemental Fig S2q**. Results of the 18 multiplex qPCR assays for MAP detection in fecal sample F21.

The results are organized into six panels based on the composition of each multiplex assay:
Left panels: Nine assays incorporating the ISMAP*02*-Irenge design.
Right panels: Nine assays incorporating the ISMAP*02*-Sevilla design.

Each row of panels corresponds to a different F*57* target design:
Top row: F*57*-Irenge
Middle row: F*57*-Herthnek
Bottom row: F*57*-Ricchi

The IS900 target design used in each multiplex is indicated by the color of the bars:
Green: IS*900*-Herthnek
Pink: IS*900*-Kim
Blue: IS*900*-Slana

n.d., both replicates were not detected; !, only one replicate was detected.

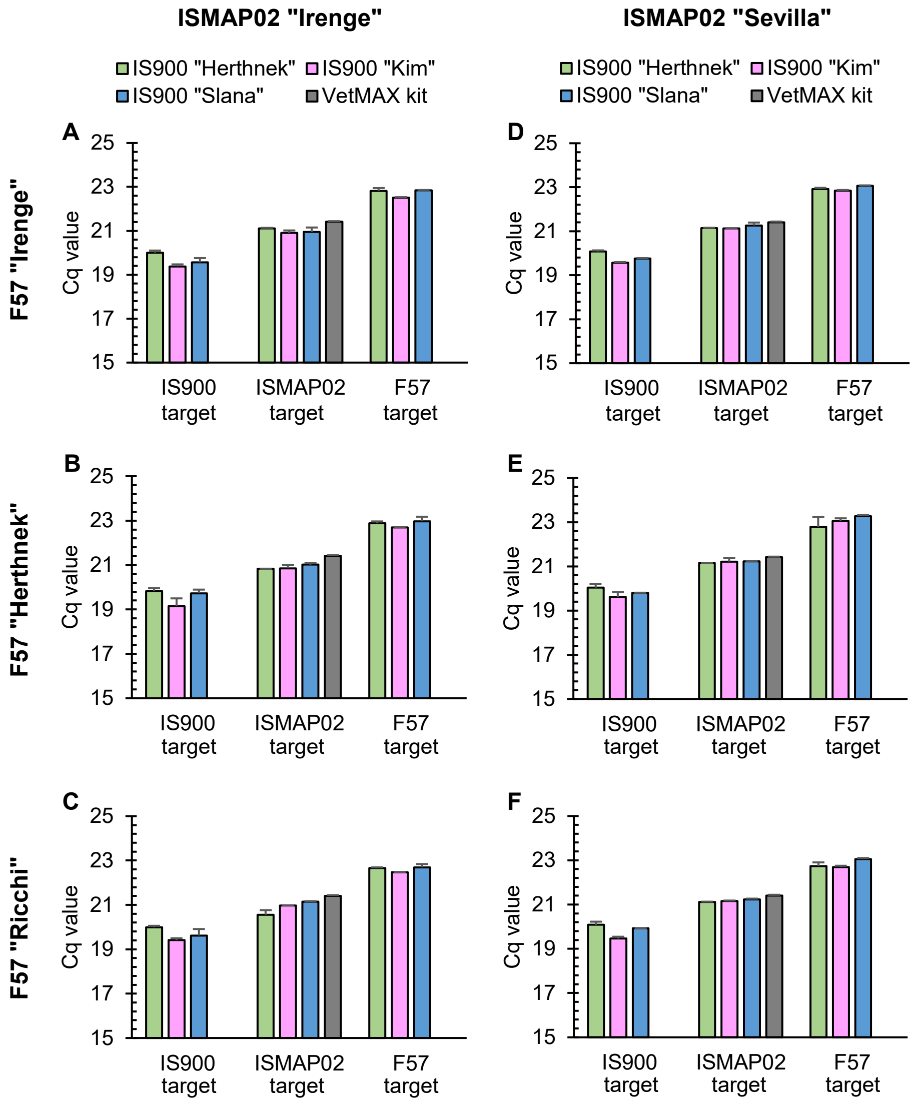


**Sample F22** (high)

**Supplemental Fig S2r**. Results of the 18 multiplex qPCR assays for MAP detection in fecal sample F22.

The results are organized into six panels based on the composition of each multiplex assay:
Left panels: Nine assays incorporating the ISMAP*02*-Irenge design.
Right panels: Nine assays incorporating the ISMAP*02*-Sevilla design.

Each row of panels corresponds to a different F*57* target design:
Top row: F*57*-Irenge
Middle row: F*57*-Herthnek
Bottom row: F*57*-Ricchi

The IS900 target design used in each multiplex is indicated by the color of the bars:
Green: IS*900*-Herthnek
Pink: IS*900*-Kim
Blue: IS*900*-Slana

n.d., both replicates were not detected; !, only one replicate was detected.

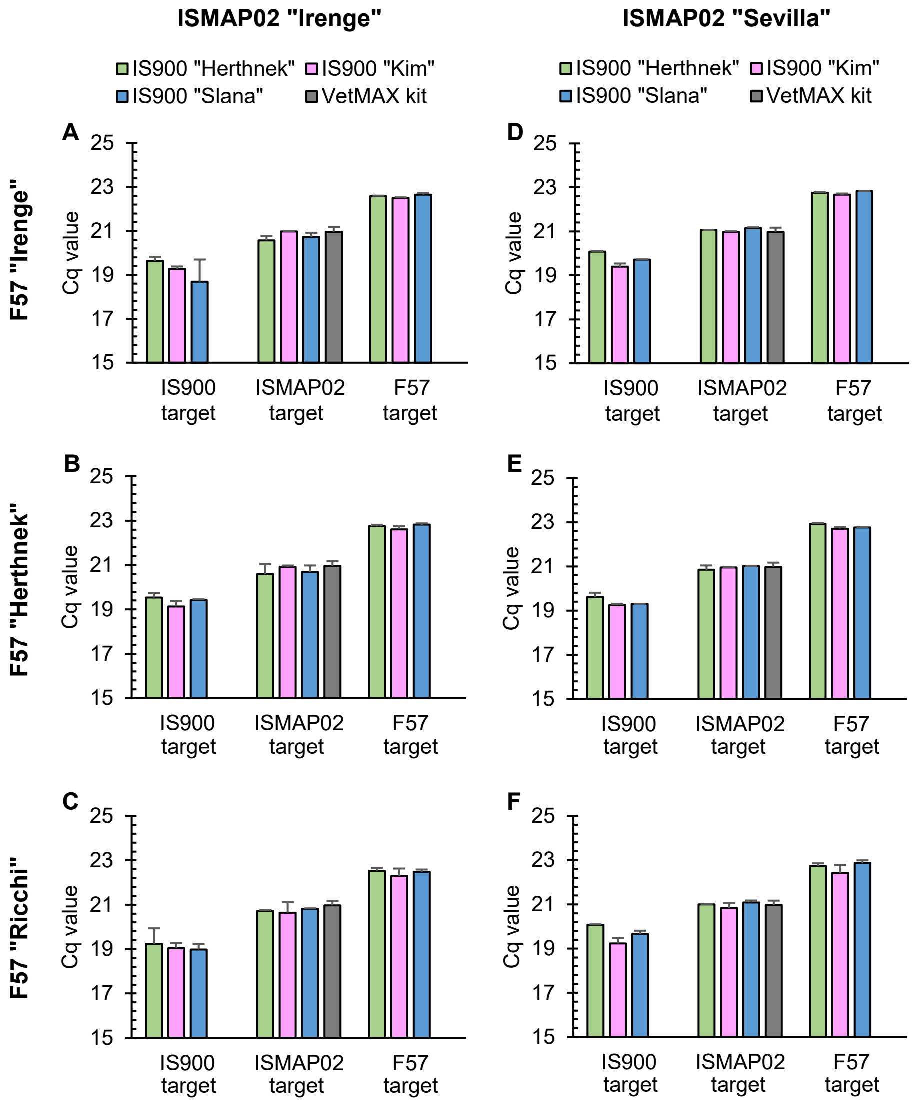


**Sample F23** (high)

**Supplemental Fig S2s**. Results of the 18 multiplex qPCR assays for MAP detection in fecal sample F23.

The results are organized into six panels based on the composition of each multiplex assay:
Left panels: Nine assays incorporating the ISMAP*02*-Irenge design.
Right panels: Nine assays incorporating the ISMAP*02*-Sevilla design.

Each row of panels corresponds to a different F*57* target design:
Top row: F*57*-Irenge
Middle row: F*57*-Herthnek
Bottom row: F*57*-Ricchi

The IS900 target design used in each multiplex is indicated by the color of the bars:
Green: IS*900*-Herthnek
Pink: IS*900*-Kim
Blue: IS*900*-Slana

n.d., both replicates were not detected; !, only one replicate was detected.

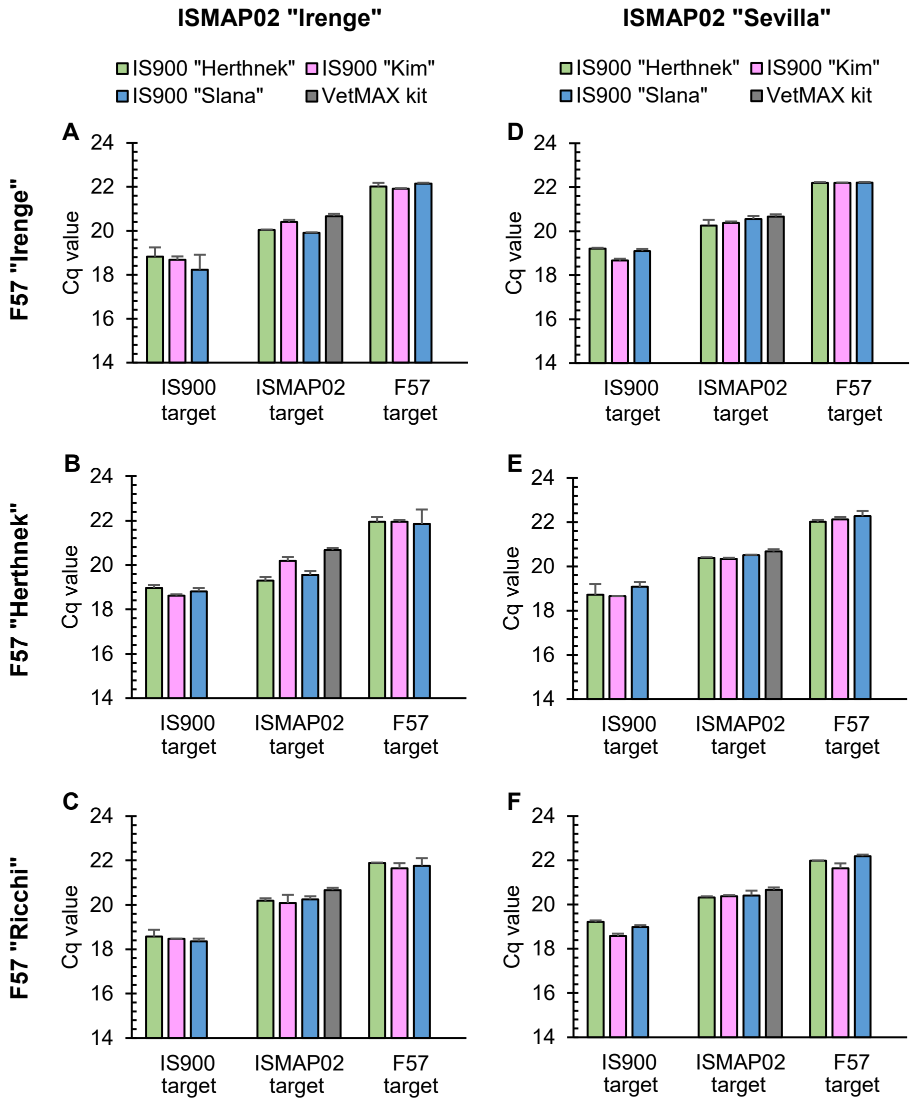


**Sample F24** (high)

**Supplemental Fig S2t**. Results of the 18 multiplex qPCR assays for MAP detection in fecal sample F24.

The results are organized into six panels based on the composition of each multiplex assay:
Left panels: Nine assays incorporating the ISMAP*02*-Irenge design.
Right panels: Nine assays incorporating the ISMAP*02*-Sevilla design.

Each row of panels corresponds to a different F*57* target design:
Top row: F*57*-Irenge
Middle row: F*57*-Herthnek
Bottom row: F*57*-Ricchi

The IS900 target design used in each multiplex is indicated by the color of the bars:
Green: IS*900*-Herthnek
Pink: IS*900*-Kim
Blue: IS*900*-Slana

n.d., both replicates were not detected; !, only one replicate was detected.

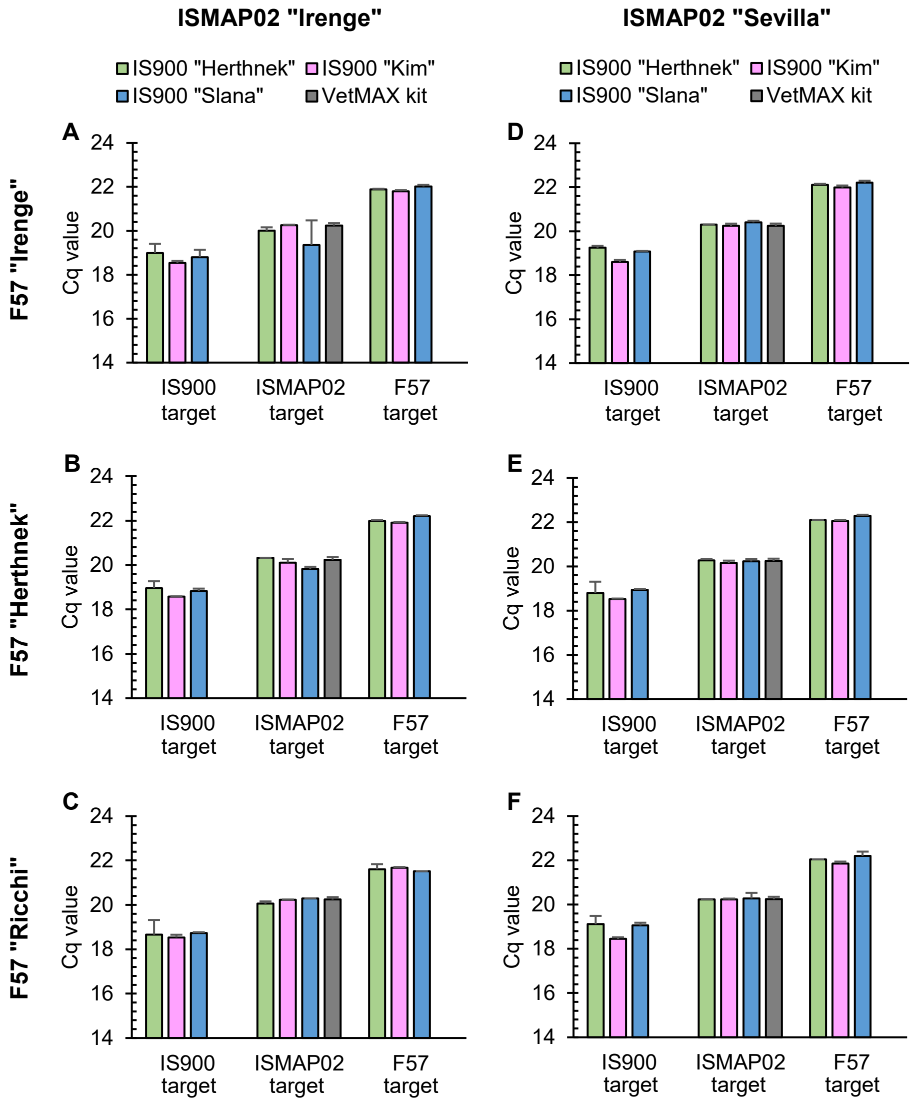


**Sample F25** (high)

**Supplemental Fig S2u**. Results of the 18 multiplex qPCR assays for MAP detection in fecal sample F25.

The results are organized into six panels based on the composition of each multiplex assay:
Left panels: Nine assays incorporating the ISMAP*02*-Irenge design.
Right panels: Nine assays incorporating the ISMAP*02*-Sevilla design.

Each row of panels corresponds to a different F*57* target design:
Top row: F*57*-Irenge
Middle row: F*57*-Herthnek
Bottom row: F*57*-Ricchi

The IS900 target design used in each multiplex is indicated by the color of the bars:
Green: IS*900*-Herthnek
Pink: IS*900*-Kim
Blue: IS*900*-Slana

n.d., both replicates were not detected; !, only one replicate was detected.

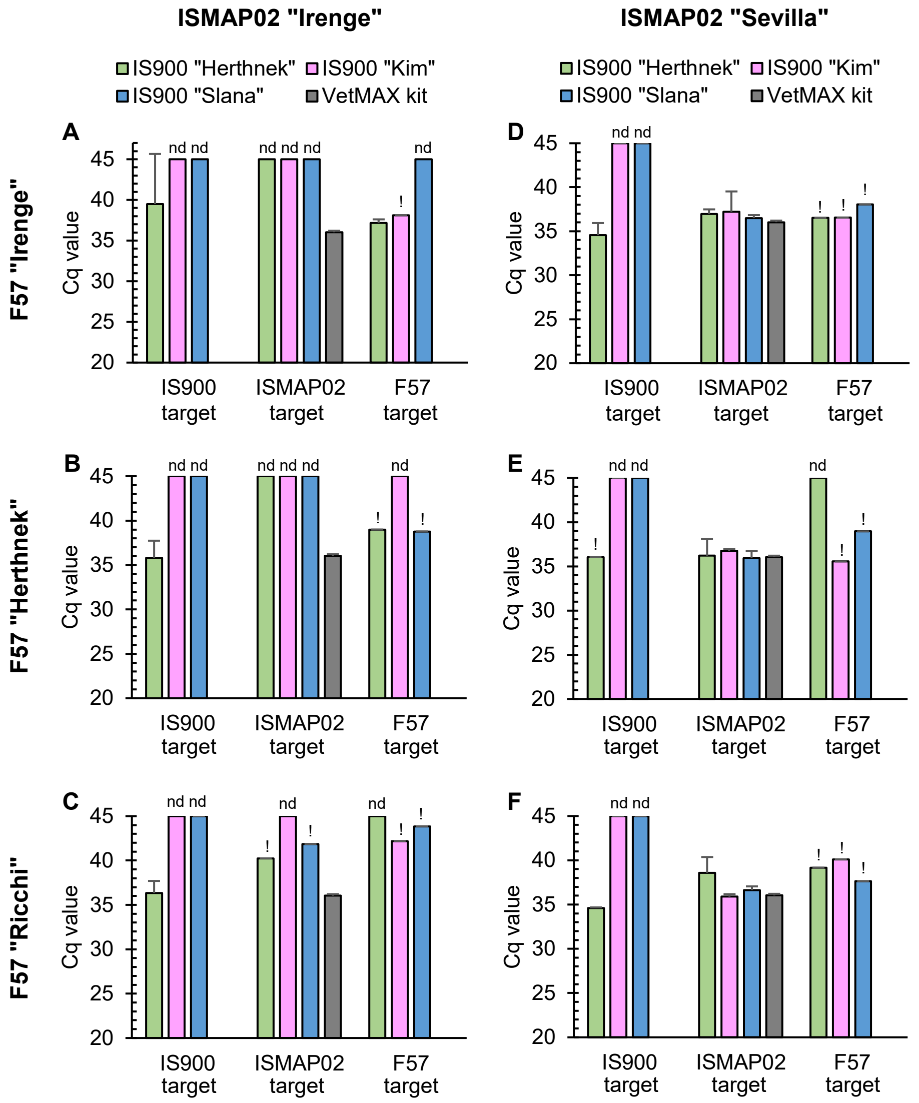


**Sample E01**

**Supplemental Fig S2v**. Results of the 18 multiplex qPCR assays for MAP detection in environmental sample E01.

The results are organized into six panels based on the composition of each multiplex assay:
Left panels: Nine assays incorporating the ISMAP*02*-Irenge design.
Right panels: Nine assays incorporating the ISMAP*02*-Sevilla design.

Each row of panels corresponds to a different F*57* target design:
Top row: F*57*-Irenge
Middle row: F*57*-Herthnek
Bottom row: F*57*-Ricchi

The IS900 target design used in each multiplex is indicated by the color of the bars:
Green: IS*900*-Herthnek
Pink: IS*900*-Kim
Blue: IS*900*-Slana

n.d., both replicates were not detected; !, only one replicate was detected.

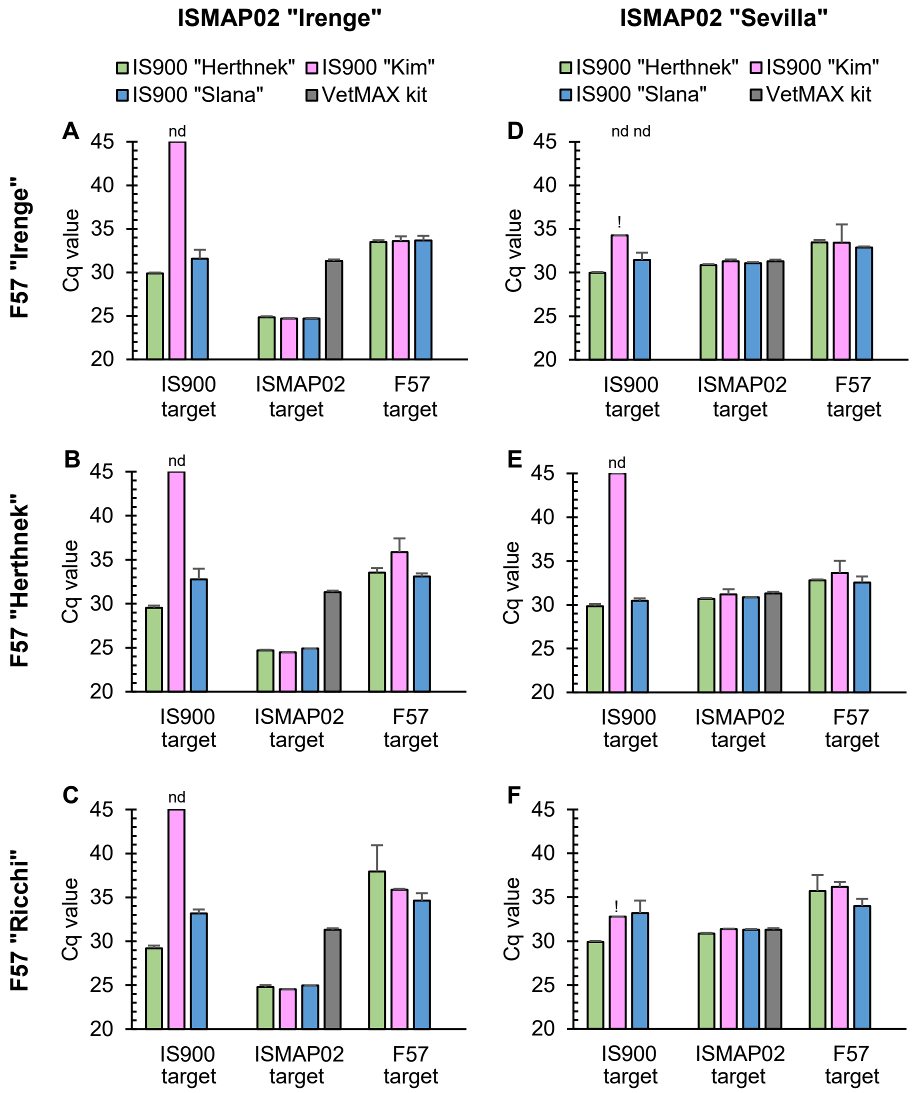


**Sample E02**

**Supplemental Fig S2w**. Results of the 18 multiplex qPCR assays for MAP detection in environmental sample E02.

The results are organized into six panels based on the composition of each multiplex assay:

Left panels: Nine assays incorporating the ISMAP*02*-Irenge design.

Right panels: Nine assays incorporating the ISMAP*02*-Sevilla design.

Each row of panels corresponds to a different F*57* target design:

Top row: F*57*-Irenge

Middle row: F*57*-Herthnek

Bottom row: F*57*-Ricchi

The IS900 target design used in each multiplex is indicated by the color of the bars:

Green: IS*900*-Herthnek

Pink: IS*900*-Kim

Blue: IS*900*-Slana

n.d., both replicates were not detected; !, only one replicate was detected.

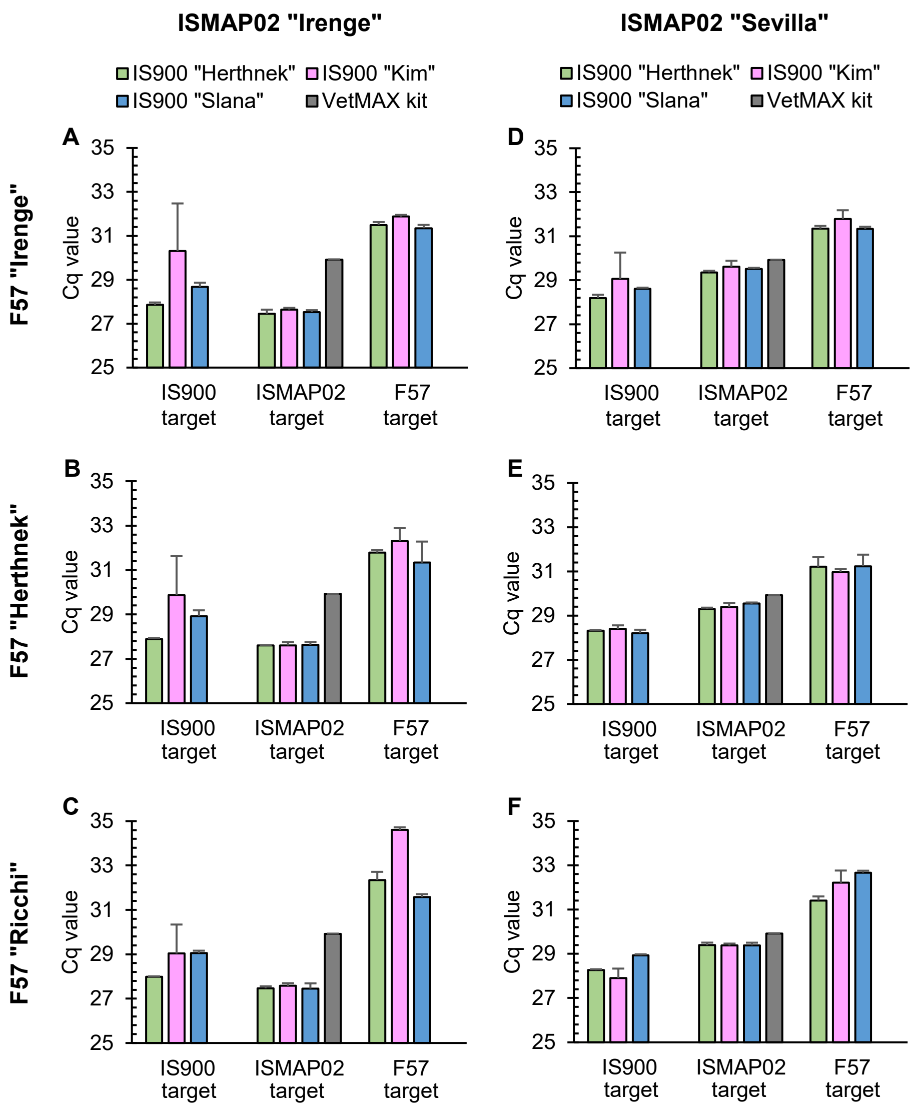


**Sample E03**

**Supplemental Fig S2x**. Results of the 18 multiplex qPCR assays for MAP detection in environmental sample E03.

The results are organized into six panels based on the composition of each multiplex assay:

Left panels: Nine assays incorporating the ISMAP*02*-Irenge design.

Right panels: Nine assays incorporating the ISMAP*02*-Sevilla design.

Each row of panels corresponds to a different F*57* target design:

Top row: F*57*-Irenge

Middle row: F*57*-Herthnek

Bottom row: F*57*-Ricchi

The IS900 target design used in each multiplex is indicated by the color of the bars:

Green: IS*900*-Herthnek

Pink: IS*900*-Kim

Blue: IS*900*-Slana

n.d., both replicates were not detected; !, only one replicate was detected.

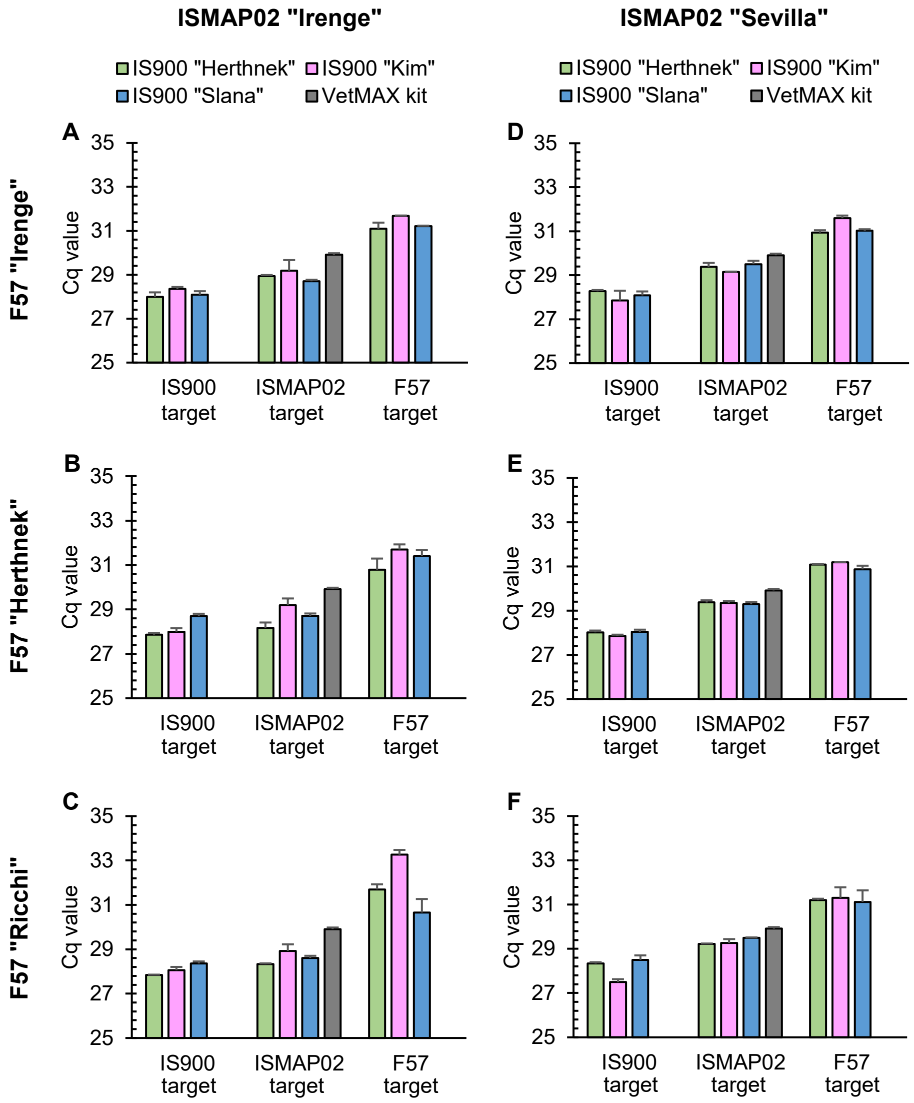


## Sample E04

**Supplemental Fig S2y**. Results of the 18 multiplex qPCR assays for MAP detection in environmental sample E04.

The results are organized into six panels based on the composition of each multiplex assay:

Left panels: Nine assays incorporating the ISMAP*02*-Irenge design.

Right panels: Nine assays incorporating the ISMAP*02*-Sevilla design.

Each row of panels corresponds to a different F*57* target design:

Top row: F*57*-Irenge

Middle row: F*57*-Herthnek

Bottom row: F*57*-Ricchi

The IS900 target design used in each multiplex is indicated by the color of the bars:

Green: IS*900*-Herthnek

Pink: IS*900*-Kim

Blue: IS*900*-Slana

n.d., both replicates were not detected; !, only one replicate was detected.

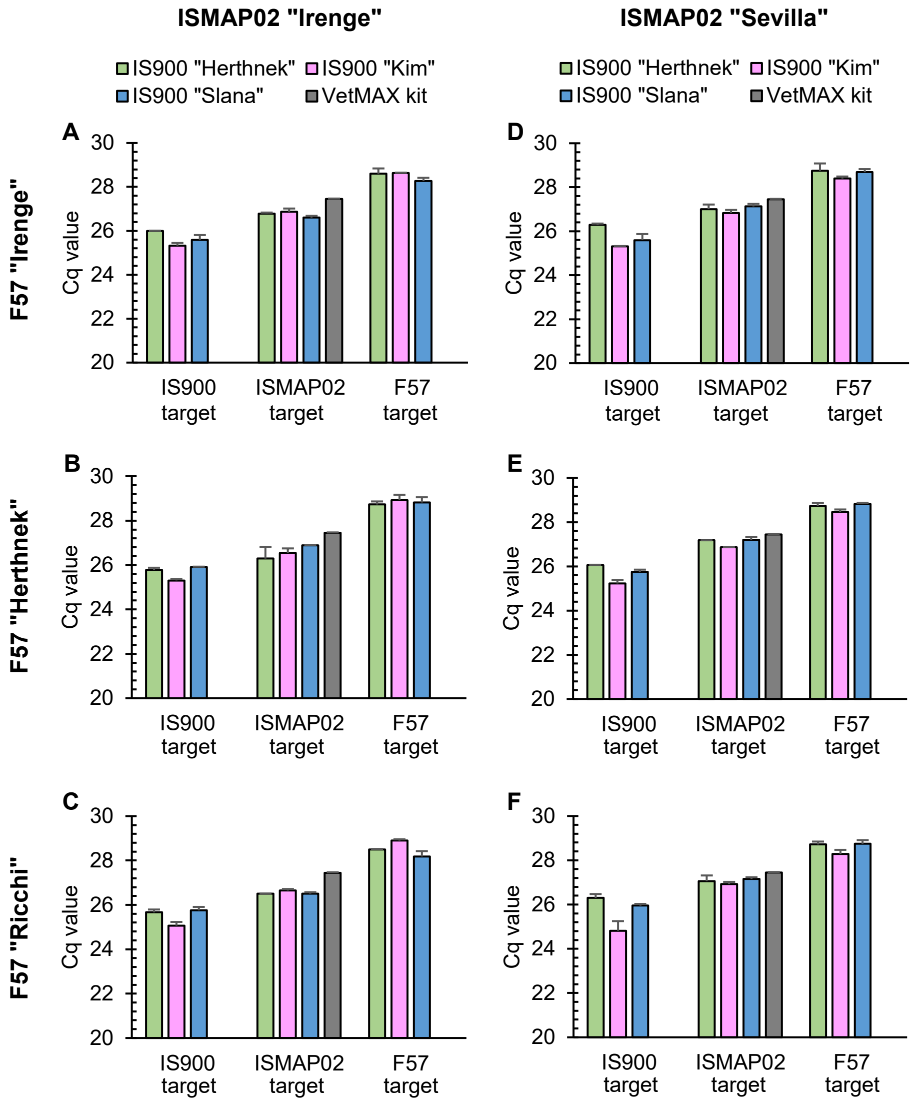


**Sample E05**

**Supplemental Fig S2z**. Results of the 18 multiplex qPCR assays for MAP detection in environmental sample E05.

The results are organized into six panels based on the composition of each multiplex assay:

Left panels: Nine assays incorporating the ISMAP*02*-Irenge design.

Right panels: Nine assays incorporating the ISMAP*02*-Sevilla design.

Each row of panels corresponds to a different F*57* target design:

Top row: F*57*-Irenge

Middle row: F*57*-Herthnek

Bottom row: F*57*-Ricchi

The IS900 target design used in each multiplex is indicated by the color of the bars:

Green: IS*900*-Herthnek

Pink: IS*900*-Kim

Blue: IS*900*-Slana

n.d., both replicates were not detected; !, only one replicate was detected.

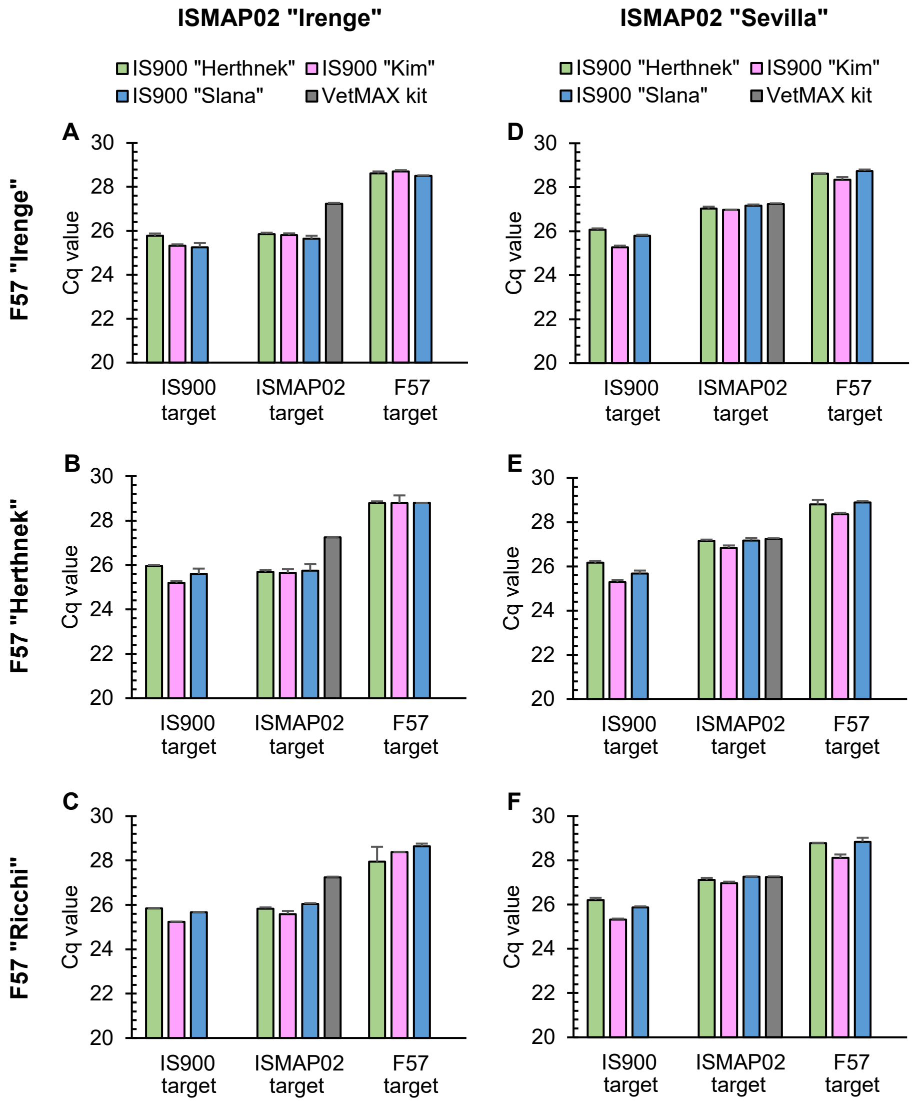


**Sample E06**

**Supplemental Fig S2aa**. Results of the 18 multiplex qPCR assays for MAP detection in environmental sample E06.

The results are organized into six panels based on the composition of each multiplex assay:

Left panels: Nine assays incorporating the ISMAP*02*-Irenge design.
Right panels: Nine assays incorporating the ISMAP*02*-Sevilla design.

Each row of panels corresponds to a different F*57* target design:

Top row: F*57*-Irenge
Middle row: F*57*-Herthnek
Bottom row: F*57*-Ricchi

The IS900 target design used in each multiplex is indicated by the color of the bars:

Green: IS*900*-Herthnek
Pink: IS*900*-Kim
Blue: IS*900*-Slana

n.d., both replicates were not detected; !, only one replicate was detected.

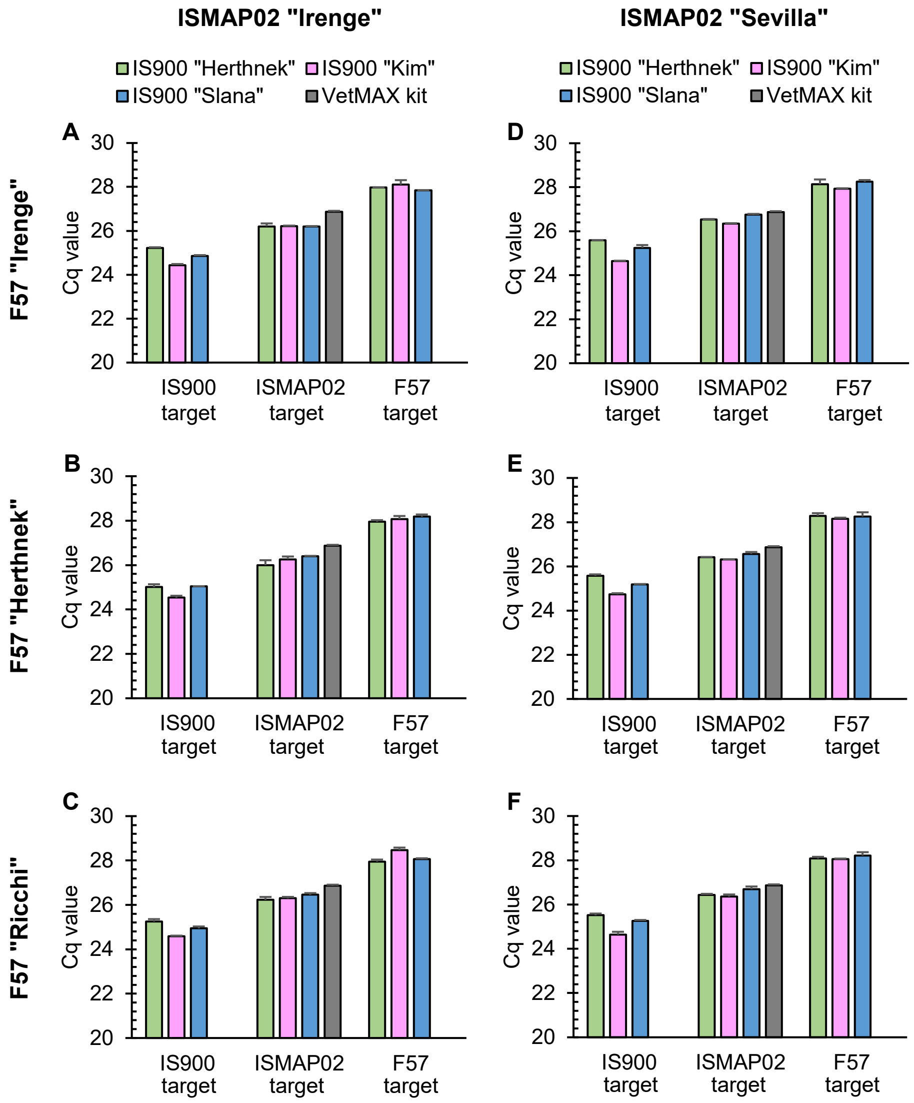


**Sample E07**

**Supplemental Fig S2ab**. Results of the 18 multiplex qPCR assays for MAP detection in environmental sample E07.

The results are organized into six panels based on the composition of each multiplex assay:
Left panels: Nine assays incorporating the ISMAP*02*-Irenge design.
Right panels: Nine assays incorporating the ISMAP*02*-Sevilla design.

Each row of panels corresponds to a different F*57* target design:
Top row: F*57*-Irenge
Middle row: F*57*-Herthnek
Bottom row: F*57*-Ricchi

The IS900 target design used in each multiplex is indicated by the color of the bars:
Green: IS*900*-Herthnek
Pink: IS*900*-Kim
Blue: IS*900*-Slana

n.d., both replicates were not detected; !, only one replicate was detected.

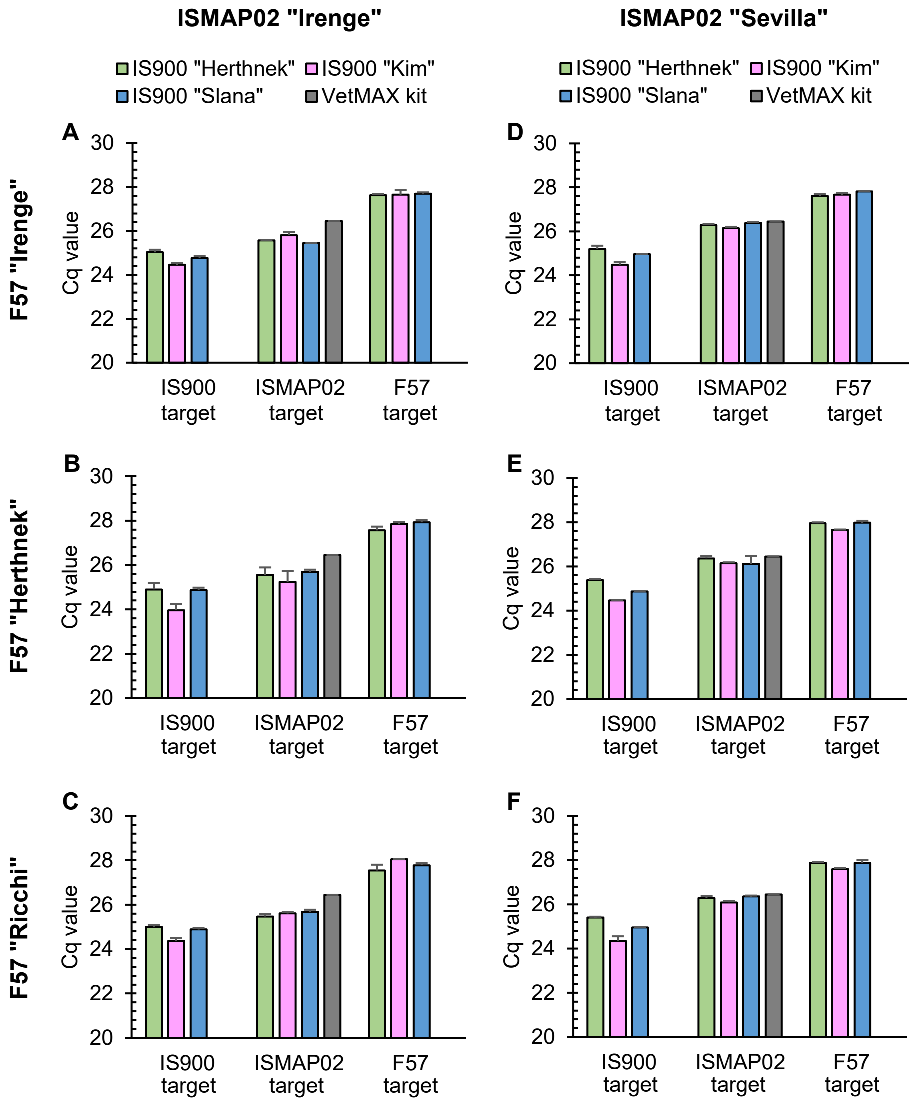


**Sample E08**

**Supplemental Fig S2ac**. Results of the 18 multiplex qPCR assays for MAP detection in environmental sample E08.

The results are organized into six panels based on the composition of each multiplex assay:

Left panels: Nine assays incorporating the ISMAP*02*-Irenge design.

Right panels: Nine assays incorporating the ISMAP*02*-Sevilla design.

Each row of panels corresponds to a different F*57* target design:

Top row: F*57*-Irenge

Middle row: F*57*-Herthnek

Bottom row: F*57*-Ricchi

The IS900 target design used in each multiplex is indicated by the color of the bars:

Green: IS*900*-Herthnek

Pink: IS*900*-Kim

Blue: IS*900*-Slana

n.d., both replicates were not detected; !, only one replicate was detected.
