# Supplementary material for: Systematic evaluation and optimization of TaqMan qPCR assays targeting F57, ISMAP02, and IS900 for multiplex detection of Mycobacterium avium subsp. paratuberculosis
Source: J Clin Microbiol. 2025 Dec 29;64(2):e00872-25. doi: 10.1128/jcm.00872-25 (PMC12892987; doi:10.1128/jcm.00872-25)
Supplement: Table S1 — Profiles (Fecal qPCR [Cq] and serum ELISA) of cows F05-F25 and Environmental qPCR results (Cq values) for E01-E08 samples. [file jcm.00872-25-s0003.docx]

**Supplemental Table S1**. Historic of the search strategy (interrogation of the databases) and the identification of articles reporting F*57* (A) and ISMAP*02* (B) PCR assays for MAP detection.

Articles were excluded if they were: (i) opinion articles; (ii) conference abstracts; (iii) thesis, or (iv) not written in English or French.

**(A) F*57* PCR assays for MAP detection**

**Aa) Interrogation of Scopus Database:**

| **#** | **Search queries** | **Results**  **(2024-05-08)** |
| --- | --- | --- |
| 1 | TITLE-ABS-KEY("mycobacterium avium subsp paratuberculosis") AND PUBYEAR > 1989 | 3383 |
| 2 | TITLE-ABS-KEY("mycobacterium avium ssp. paratuberculosis") AND PUBYEAR > 1989 | 360 |
| 3 | TITLE-ABS-KEY("mycobacterium avium subspecies paratuberculosis") AND PUBYEAR > 1989 | 1153 |
| 4 | TITLE-ABS-KEY ("Mycobacterium avium paratuberculosis") AND PUBYEAR > 1989 | 215 |
| 5 | 1 or 2 or 3 or 4 | 3886 |
| 6 | TITLE-ABS-KEY("polymerase chain reaction" or "PCR" > 1989 | 1530235 |
| 7 | TITLE-ABS-KEY("F57") AND PUBYEAR > 1989 | 162 |
| 8 | 5 and 6 and 7 | 81 |
| 9 | 8 and (LIMIT-TO(LANGUAGE, "English") OR (LIMIT-TO(LANGUAGE, "French"))) | 80 |

**Ab) Interrogation of Agricola (Ovid) Database:**

| **#** | **Search queries** | **Results**  **(2024-05-08)** |
| --- | --- | --- |
| 1 | "mycobacterium avium subsp paratuberculosis".mp. | 2050 |
| 2 | limit 1 to yr="1990 -Current" | 1862 |
| 3 | "mycobacterium avium ssp. paratuberculosis".mp. | 233 |
| 4 | limit 3 to yr="1990 -Current" | 233 |
| 5 | "mycobacterium avium subspecies paratuberculosis".mp. | 484 |
| 6 | limit 5 to yr="1990 -Current" | 484 |
| 7 | "Mycobacterium avium paratuberculosis".mp. | 54 |
| 8 | limit 7 to yr="1990 -Current" | 53 |
| 9 | 2 or 4 or 6 or 8 | 1980 |
| 10 | "polymerase chain reaction" or "PCR".mp. | 173407 |
| 11 | limit 9 to yr="1990 -Current" | 173329 |
| 12 | "F57".mp. | 97 |
| 13 | limit 10 to yr="1990 -Current" | 96 |
| 14 | 9 and 11 and 13 | 42 |
| 15 | limit 14 to (english or french) | 42 |

**Ac) Interrogation of Biological Abstracts (Ovid) Database:**

| **#** | **Search queries** | **Results**  **(2024-05-08)** |
| --- | --- | --- |
| 1 | "mycobacterium avium subsp paratuberculosis".mp. | 1980 |
| 2 | limit 1 to yr="1990 -Current" | 1979 |
| 3 | "mycobacterium avium ssp. paratuberculosis".mp. | 490 |
| 4 | limit 3 to yr="1990 -Current" | 489 |
| 5 | "mycobacterium avium subspecies paratuberculosis".mp. | 1060 |
| 6 | limit 5 to yr="1990 -Current" | 1060 |
| 7 | "Mycobacterium avium paratuberculosis".mp. | 3225 |
| 8 | limit 7 to yr="1990 -Current" | 3225 |
| 9 | 2 or 4 or 6 or 8 | 3751 |
| 10 | "polymerase chain reaction" or "PCR". mp. | 1000678 |
| 11 | limit 10 to yr="1990 -Current" | 999627 |
| 12 | "F57".mp. | 179 |
| 13 | limit 12 to yr="1990 -Current" | 178 |
| 14 | 9 and 11 and 13 | 73 |
| 15 | limit 14 to (english or french) | 72 |

**Ad) Interrogation of CAB Abstracts (Ovid) 1990 to 2024 Database:**

| **#** | **Search queries** | **Results**  **(2024-05-08)** |
| --- | --- | --- |
| 1 | "mycobacterium avium subsp paratuberculosis".mp. | 5643 |
| 2 | limit 1 to yr="1990 -Current" | 5643 |
| 3 | "mycobacterium avium ssp. paratuberculosis".mp. | 320 |
| 4 | limit 3 to yr="1990 -Current" | 320 |
| 5 | "mycobacterium avium subspecies paratuberculosis".mp. | 970 |
| 6 | limit 5 to yr="1990 -Current" | 970 |
| 7 | "Mycobacterium avium paratuberculosis".mp. | 122 |
| 8 | limit 7 to yr="1990 -Current" | 122 |
| 9 | 2 or 4 or 6 or 8 | 5744 |
| 10 | "polymerase chain reaction" or "PCR".mp. | 351261 |
| 11 | limit 10 to yr="1990 -Current" | 351260 |
| 12 | "F57".mp. | 205 |
| 13 | limit 12 to yr="1990 -Current" | 205 |
| 14 | 9 and 11 and 13 | 78 |
| 15 | limit 14 to (english or french) | 70 |
|  |  |  |

**Ae) Interrogation of FSTA (Ovid) Database:**

| **#** | **Search queries** | **Results**  **(2024-05-08)** |
| --- | --- | --- |
| 1 | "mycobacterium avium subsp paratuberculosis".mp. | 308 |
| 2 | limit 1 to yr="1990 -Current" | 308 |
| 3 | "mycobacterium avium ssp. paratuberculosis".mp. | 75 |
| 4 | limit 3 to yr="1990 -Current" | 75 |
| 5 | "mycobacterium avium subspecies paratuberculosis".mp. | 93 |
| 6 | limit 5 to yr="1990 -Current" | 93 |
| 7 | "Mycobacterium avium paratuberculosis".mp. | 24 |
| 8 | limit 7 to yr="1990 -Current" | 24 |
| 9 | 2 or 4 or 6 or 8 | 419 |
| 10 | "polymerase chain reaction" or "PCR".mp. | 39044 |
| 11 | limit 10 to yr="1990 -Current" | 39001 |
| 12 | "F57".mp. | 41 |
| 13 | limit 12 to yr="1990 -Current" | 41 |
| 14 | 9 and 11 and 13 | 22 |
| 15 | limit 14 to (english or french) | 22 |

**Af) Interrogation of Medline (Ovid) Database:**

| **#** | **Search queries** | **Results**  **(2024-05-08)** |
| --- | --- | --- |
| 1 | "mycobacterium avium subsp paratuberculosis".mp. | 3122 |
| 2 | limit 1 to yr="1990 -Current" | 3049 |
| 3 | "mycobacterium avium ssp. paratuberculosis".mp. | 317 |
| 4 | limit 3 to yr="1990 -Current" | 317 |
| 5 | "mycobacterium avium subspecies paratuberculosis".mp. | 968 |
| 6 | limit 5 to yr="1990 -Current" | 968 |
| 7 | "Mycobacterium avium paratuberculosis".mp. | 127 |
| 8 | limit 7 to yr="1990 -Current" | 127 |
| 9 | 2 or 4 or 6 or 8 | 3374 |
| 10 | "polymerase chain reaction" or "PCR".mp. | 987437 |
| 11 | limit 10 to yr="1990 -Current" | 985967 |
| 12 | "F57".mp. | 114 |
| 13 | limit 10 to yr="1990 -Current" | 110 |
| 14 | 9 and 11 and 13 | 57 |
| 15 | limit 14 to (english or french) | 56 |

**Ag) F*57* Publications**

TaqMan assays:

1. (Ricchi et al., 2016, Butot et al., 2019, Sange et al., 2019a, Russo et al., 2023)
2. (Donaghy et al., 2011, Pagliasso et al., 2021)
3. (Herthnek and Bolske, 2006, Meadus et al., 2008, Salgado et al., 2009, Salgado et al., 2011, Sidoti et al., 2011, Banche et al., 2015)
4. (Irenge et al., 2009b)
5. (Schönenbrücher et al., 2008, Fawzy et al., 2015, Selim and Gaede, 2015)
6. (Singh et al., 2014, Rojas-Ponce et al., 2022)
7. (Hruška et al., 2005, Slana et al., 2008, Slana et al., 2009, Khol et al., 2010a, Khol et al., 2010b, Kralik et al., 2010, Hruska et al., 2011, Kralik et al., 2011, Pribylova et al., 2011, Slana et al., 2011, Klanicova et al., 2012, Kralik et al., 2012, Pribylova et al., 2012, Forde et al., 2013, Kralik et al., 2014, Han et al., 2015b, Wolf et al., 2015, Khol et al., 2017, Corbett et al., 2018, Rhim et al., 2018, Husakova et al., 2020)

**Seven unique F*57* TaqMan assays (original publications):** (Herthnek and Bolske, 2006, Schönenbrücher et al., 2008, Slana et al., 2008, Irenge et al., 2009b, Donaghy et al., 2011, Singh et al., 2014, Ricchi et al., 2016)

SYBR Green chemistry: (Stephan et al., 2007, Seva et al., 2014, Blaiotta et al., 2016, de Kruijf et al., 2017a, Chaitanya et al., 2019, Chaitanya et al., 2021, Nouri et al., 2024)

Commercial diagnostic kits: (Elguezabal et al., 2011, Özpinar et al., 2015, Correa-Valencia et al., 2017, Hanifian, 2020, Jurado-Martos et al., 2023, Maldonado-Castro et al., 2023)

Others: (Coetsier et al., 2000, Enosawa et al., 2003, Vansnick et al., 2004, Godfroid et al., 2005, Tasara et al., 2005, Tasara and Stephan, 2005, Bosshard et al., 2006, Möbius et al., 2008, Selvam et al., 2009, Rocca et al., 2010, Tripathi and Stevenson, 2010, Akineden et al., 2011, Timms et al., 2011, Stief et al., 2012, El-Sayed et al., 2013, Hanifian et al., 2013, Mohan et al., 2013, Muñoz et al., 2013, Hanifian, 2014, Keller et al., 2014, Rani et al., 2014, Youssef et al., 2014, Timms et al., 2015, Hanifian and Khani, 2016, Ritter et al., 2016, Keshavarz et al., 2018, Pisanu et al., 2018, Karthikeyan et al., 2019, De Grossi et al., 2020, Sadeghi et al., 2020, Beinhauerova et al., 2021, Badia-Bringué et al., 2022, Moghaddam et al., 2023, Rasper-Hossinger et al., 2023)

**(B) ISMAP*02* PCR assays for MAP detection**

**Ba) Interrogation of Scopus Database:**

| **#** | **Search queries** | **Results**  **(2024-05-08)** |
| --- | --- | --- |
| 1 | TITLE-ABS-KEY("mycobacterium avium subsp paratuberculosis") AND PUBYEAR > 1989 | 3383 |
| 2 | TITLE-ABS-KEY("mycobacterium avium ssp. paratuberculosis") AND PUBYEAR > 1989 | 360 |
| 3 | TITLE-ABS-KEY("mycobacterium avium subspecies paratuberculosis") AND PUBYEAR > 1989 | 1153 |
| 4 | TITLE-ABS-KEY ("Mycobacterium avium paratuberculosis") AND PUBYEAR > 1989 | 215 |
| 5 | 1 or 2 or 3 or 4 | 3886 |
| 6 | TITLE-ABS-KEY("polymerase chain reaction" or "PCR" > 1989 | 1530235 |
| 7 | TITLE-ABS-KEY("ISMAP02") AND PUBYEAR > 1989 | 24 |
| 8 | 5 and 6 and 7 | 22 |
| 9 | 8 and (LIMIT-TO(LANGUAGE, "English") OR (LIMIT-TO(LANGUAGE, "French"))) | 22 |

**Bb) Interrogation of Agricola (Ovid) Database:**

| **#** | **Search queries** | **Results**  **(2024-05-08)** |
| --- | --- | --- |
| 1 | "mycobacterium avium subsp paratuberculosis".mp. | 2050 |
| 2 | limit 1 to yr="1990 -Current" | 1862 |
| 3 | "mycobacterium avium ssp. paratuberculosis".mp. | 233 |
| 4 | limit 3 to yr="1990 -Current" | 233 |
| 5 | "mycobacterium avium subspecies paratuberculosis".mp. | 484 |
| 6 | limit 5 to yr="1990 -Current" | 484 |
| 7 | "Mycobacterium avium paratuberculosis".mp. | 54 |
| 8 | limit 7 to yr="1990 -Current" | 53 |
| 9 | 2 or 4 or 6 or 8 | 1980 |
| 10 | "polymerase chain reaction" or "PCR".mp. | 173407 |
| 11 | limit 10 to yr="1990 -Current" | 173329 |
| 12 | "ISMAP02".mp. | 12 |
| 13 | limit 12 to yr="1990 -Current" | 12 |
| 14 | 9 and 11 and 13 | 10 |
| 15 | limit 14 to (english or french) | 10 |

**Bc) Interrogation of Biological Abstracts (Ovid) Database:**

| **#** | **Search queries** | **Results**  **(2024-05-08)** |
| --- | --- | --- |
| 1 | "mycobacterium avium subsp paratuberculosis".mp. | 1980 |
| 2 | limit 1 to yr="1990 -Current" | 1979 |
| 3 | "mycobacterium avium ssp. paratuberculosis".mp. | 490 |
| 4 | limit 3 to yr="1990 -Current" | 489 |
| 5 | "mycobacterium avium subspecies paratuberculosis".mp. | 1060 |
| 6 | limit 5 to yr="1990 -Current" | 1060 |
| 7 | "Mycobacterium avium paratuberculosis".mp. | 3225 |
| 8 | limit 7 to yr="1990 -Current" | 3225 |
| 9 | 2 or 4 or 6 or 8 | 3751 |
| 10 | "polymerase chain reaction" or "PCR".mp. | 1000678 |
| 11 | limit 10 to yr="1990 -Current" | 999627 |
| 12 | "ISMAP02".mp. | 22 |
| 13 | limit 12 to yr="1990 -Current" | 22 |
| 14 | 9 and 11 and 13 | 18 |
| 15 | limit 14 to (english or french) | 18 |

**Bd) Interrogation of CAB Abstracts (Ovid) 1990 to 2024 Database:**

| **#** | **Search queries** | **Results**  **(2024-05-08)** |
| --- | --- | --- |
| 1 | "mycobacterium avium subsp paratuberculosis".mp. | 5643 |
| 2 | limit 1 to yr="1990 -Current" | 5643 |
| 3 | "mycobacterium avium ssp. paratuberculosis".mp. | 320 |
| 4 | limit 3 to yr="1990 -Current" | 320 |
| 5 | "mycobacterium avium subspecies paratuberculosis".mp. | 970 |
| 6 | limit 5 to yr="1990 -Current" | 970 |
| 7 | "Mycobacterium avium paratuberculosis".mp. | 122 |
| 8 | limit 7 to yr="1990 -Current" | 122 |
| 9 | 2 or 4 or 6 or 8 | 5744 |
| 10 | "polymerase chain reaction" or "PCR".mp. | 351261 |
| 11 | limit 10 to yr="1990 -Current" | 351260 |
| 12 | "ISMAP02".mp. | 19 |
| 13 | limit 12 to yr="1990 -Current" | 19 |
| 14 | 9 and 11 and 13 | 17 |
| 15 | limit 14 to (english or french) | 17 |
|  |  |  |

**Be) Interrogation of FSTA (Ovid) Database:**

| **#** | **Search queries** | **Results**  **(2024-05-08)** |
| --- | --- | --- |
| 1 | "mycobacterium avium subsp paratuberculosis".mp. | 308 |
| 2 | limit 1 to yr="1990 -Current" | 308 |
| 3 | "mycobacterium avium ssp. paratuberculosis".mp. | 75 |
| 4 | limit 3 to yr="1990 -Current" | 75 |
| 5 | "mycobacterium avium subspecies paratuberculosis".mp. | 93 |
| 6 | limit 5 to yr="1990 -Current" | 93 |
| 7 | "Mycobacterium avium paratuberculosis".mp. | 24 |
| 8 | limit 7 to yr="1990 -Current" | 24 |
| 9 | 2 or 4 or 6 or 8 | 419 |
| 10 | "polymerase chain reaction" or "PCR".mp. | 39044 |
| 11 | limit 10 to yr="1990 -Current" | 39001 |
| 12 | "ISMAP02".mp. | 3 |
| 13 | limit 12 to yr="1990 -Current" | 3 |
| 14 | 9 and 11 and 13 | 2 |
| 15 | limit 14 to (english or french) | 2 |

**Bf) Interrogation of Medline (Ovid) Database:**

| **#** | **Search queries** | **Results**  **(2024-05-08)** |
| --- | --- | --- |
| 1 | "mycobacterium avium subsp paratuberculosis".mp. | 3122 |
| 2 | limit 1 to yr="1990 -Current" | 3049 |
| 3 | "mycobacterium avium ssp. paratuberculosis".mp. | 317 |
| 4 | limit 3 to yr="1990 -Current" | 317 |
| 5 | "mycobacterium avium subspecies paratuberculosis".mp. | 968 |
| 6 | limit 5 to yr="1990 -Current" | 968 |
| 7 | "Mycobacterium avium paratuberculosis".mp. | 127 |
| 8 | limit 7 to yr="1990 -Current" | 127 |
| 9 | 2 or 4 or 6 or 8 | 3374 |
| 10 | "polymerase chain reaction" or "PCR".mp. | 987437 |
| 11 | limit 10 to yr="1990 -Current" | 985967 |
| 12 | "ISMAP02".mp. | 20 |
| 13 | limit 12 to yr="1990 -Current" | 20 |
| 14 | 9 and 11 and 13 | 19 |
| 15 | limit 14 to (english or french) | 19 |

**Bg)** **ISMAP*02* Publications**

TaqMan assays:

1. (Irenge et al., 2009a, Han et al., 2015a)
2. (Sobrino et al., 2011, Sevilla et al., 2014)

**Two unique ISMAP*02* TaqMan assays** (original publication): (Irenge et al., 2009a, Sevilla et al., 2014)

SYBR Green chemistry: (Douarre et al., 2010, de Kruijf et al., 2017b, Nouri et al., 2024)

Commercial diagnostic kits: (Maio et al., 2011, Forde et al., 2013, Alajmi et al., 2016, Arango-Sabogal et al., 2024)

Others: (Stabel and Bannantine, 2005, Vijayarani and Kumaman, 2008, Pithua et al., 2010, Rad et al., 2010, Pithua et al., 2011a, Pithua et al., 2011b, Hanifian et al., 2013, Leite et al., 2013, Park et al., 2016, Rani et al., 2018, Rhim et al., 2018, Sange et al., 2019b)

**Search Records – publications:**

Akineden, Ö., J. A. Fernández-Silva, S. Weirich, A. Abdulmawjood, and M. Bülte. 2011. Comparison of two decontamination procedures, three culture media, and real time-PCR assay for the detection of Mycobacterium avium subsp. Paratubercuiosis (MAP) from artificially contaminated raw sausage. Archiv fur Lebensmittelhygiene 62(5):150-156.

Alajmi, A., G. Klein, N. T. Grabowski, S. Fohler, O. Akineden, and A. Abdulmawjood. 2016. Evaluation of a Commercial Real-Time PCR Kit for the Detection of Mycobacterium avium subspecies paratuberculosis in Milk. Curr. Microbiol. 73(5):668-675.

Arango-Sabogal, J. C., O. Labrecque, J. H. Fairbrother, S. Buczinski, J. P. Roy, J. Arsenault, V. Wellemans, and G. Fecteau. 2024. Comparison of 2 PCR assays on environmental samples cultured for Mycobacterium avium subsp. paratuberculosis. J. Vet. Diagn. Invest. 36(1):24-31.

Badia-Bringué, G., M. Canive, R. Casais, C. Blanco-Vázquez, J. Amado, N. Iglesias, A. González, M. Bascones, R. A. Juste, and M. Alonso-Hearn. 2022. Evaluation of a droplet digital PCR assay for quantification of Mycobacterium avium subsp. paratuberculosis DNA in whole-blood and fecal samples from MAP-infected Holstein cattle. Front. Vet. Sci. 9.

Banche, G., V. Allizond, R. Sostegni, A. Lavagna, M. Bergallo, F. Sidoti, M. Daperno, R. Rocca, and A. M. Cuffini. 2015. Application of multiple laboratory tests for Mycobacterium avium ssp. Paratuberculosis detection in Crohn's disease patient specimens. New Microbiol. 38(3):357-367.

Beinhauerova, M., M. Beinhauerova, S. McCallum, E. Sellal, M. Ricchi, R. O’Brien, B. Blanchard, I. Slana, V. Babak, and P. Kralik. 2021. Development of a reference standard for the detection and quantification of Mycobacterium avium subsp. paratuberculosis by quantitative PCR. Sci. Rep. 11(1).

Blaiotta, G., A. Di Cerbo, N. Murru, R. Coppola, and M. Aponte. 2016. Persistence of bacterial indicators and zoonotic pathogens in contaminated cattle wastes. BMC Microbiol. 16(1).

Bosshard, C., R. Stephan, and T. Tasara. 2006. Application of an F57 sequence-based real-time PCR assay for Mycobacterium paratuberculosis detection in bulk tank raw milk and slaughtered healthy dairy cows. J. Food Protection 69(7):1662-1667.

Butot, S., M. Ricchi, I. A. Sevilla, L. Michot, E. Molina, M. Tello, S. Russo, N. Arrigoni, J. M. Garrido, and D. Tomas. 2019. Estimation of performance characteristics of analytical methods for Mycobacterium avium subsp. paratuberculosis detection in dairy products. Front. Microbiol. 10(MAR).

Chaitanya, R. K., G. Priyanka, and B. Sreedevi. 2021. Evaluation of real time PCR for the detection of Mycobacterium avium subsp. paratuberculosis in faecal samples of cattle. Journal of Veterinary and Animal Sciences 52(4):414-417.

Chaitanya, R. K., Y. K. Reddy, G. D. Raj, and A. Thangavelu. 2019. Quantification of Mycobacterium avium subsp. paratuberculosis from the tissues of challenged mice using SYBR Green real time PCR assay for the assessment of vaccine efficacy. Ind. J. Ani. Res 53(7):944-948.

Coetsier, C., P. Vannuffel, N. Blondeel, J. F. Denef, C. Cocito, and J. L. Gala. 2000. Duplex PCR for differential identification of Mycobactelium bovis, M. avium, and M. avium subsp. paratuberculosis in formalin-fixed paraffin-embedded tissues from cattle. J. Clin. Microbiol. 38(8):3048-3054.

Corbett, C. S., H. W. Barkema, and J. De Buck. 2018. Quantifying fecal shedding of Mycobacterium avium ssp. paratuberculosis from calves after experimental infection and exposure. J. Dairy Sci. 101(2):1478-1487.

Correa-Valencia, N. M., N. F. Ramírez, M. Bülte, and J. A. Fernández-Silva. 2017. Fecal culture and two fecal-PCR methods for the diagnosis of mycobacterium avium subsp. Paratuberculosis in a seropositive herd. Revista Colombiana Ciencias Pecuarias 30(2):101-115.

De Grossi, L., D. Santori, A. Barone, S. Abbruzzese, M. Ricchi, and G. A. Marcario. 2020. Isolation of mycobacterium avium subsp. Paratuberculosis in the feces and tissue of small ruminants using a non-automated liquid culture method. Animals 10(1).

de Kruijf, M., A. Coffey, and J. O'Mahony. 2017a. The investigation of the truncated mbtA gene within the mycobactin cluster of Mycobacterium avium subspecies paratuberculosis as a novel diagnostic marker for real-time PCR. J. Microbiol. Methods 136:40-48.

de Kruijf, M., R. Govender, D. Yearsley, A. Coffey, and J. O'Mahony. 2017b. A comparative study evaluating the efficacy of IS_MAP04 with IS900 and IS_MAP02 as a new diagnostic target for the detection of Mycobacterium avium subspecies paratuberculosis from bovine faeces. Veterinary microbiology. 204:104-109.

Donaghy, J. A., J. Johnston, and M. T. Rowe. 2011. Detection of Mycobacterium avium ssp. paratuberculosis in cheese, milk powder and milk using IS900 and f57-based qPCR assays. J. Appl. Microbiol. 110(2):479-489.

Douarre, P. E., W. Cashman, J. Buckley, A. Coffey, and J. M. O'Mahony. 2010. Isolation and detection of Mycobacterium avium subsp. paratuberculosis (MAP) from cattle in Ireland using both traditional culture and molecular based methods. Gut Pathogens 2(1).

El-Sayed, A., S. Natour, N. E. M. I. Abdou, M. Salem, A. Hassan, W. Wolter, and M. Zschock. 2013. Detection of Mycobacterium avium subsp. paratuberculosis in manure and milk filters of apparently healthy dairy herds in Hesse, Germany. The Journal of American Science 9(1):469-473.

Elguezabal, N., F. Bastida, I. A. Sevilla, N. Gonzalez, E. Molina, J. M. Garrido, and R. A. Juste. 2011. Estimation of Mycobacterium avium subsp. paratuberculosis growth parameters: Strain characterization and comparison of methods. Appl. Environ. Microbiol. 77(24):8615-8624.

Enosawa, M., S. Kageyama, K. Sawai, K. Watanabe, T. Notomi, S. Onoe, Y. Mori, and Y. Yokomizo. 2003. Use of loop-mediated isothermal amplification of the IS900 sequence for rapid detection of cultured Mycobacterium avium subsp. paratuberculosis. J. Clin. Microbiol. 41(9):4359-4365.

Fawzy, A., T. Eisenberg, A. El-Sayed, and M. Zschock. 2015. Improvement of sensitivity for Mycobacterium avium subsp. paratuberculosis (MAP) detection in bovine fecal samples by specific duplex F57/IC real-time and conventional IS900 PCRs after solid culture enrichment. Trop. Anim. Health Prod. 47(4):721-726.

Forde, T., J. De Buck, B. Elkin, S. Kutz, F. V. der Meer, and K. Orsel. 2013. Contrasting results of culture-dependent and molecular analyses of mycobacterium avium subsp. Paratuberculosis from wood bison. Appl. Environ. Microbiol. 79(14):4448-4454.

Godfroid, J., C. Delcorps, L. M. Irenge, K. Walravens, S. Marché, and J. L. Gala. 2005. Definitive differentiation between single and mixed mycobacterial infections in red deer (Cervus elaphus) by a combination of duplex amplification of p34 and f57 sequences and Hpy188I enzymatic restriction of duplex amplicons. J. Clin. Microbiol. 43(9):4640-4648.

Han, J., Y. Jung, C. Choe, J. Yoo, S. Kang, H. Yoo, H. Park, E. Kwon, and Y. Cho. 2015a. Multiplex quantitative real-time polymerase chain reaction assay for rapid detection of Mycobacterium avium subsp. paratuberculosis in fecal samples. J. Vet. Clin. 32(3):219-223.

Han, J. I., Y. H. Jung, C. Choe, J. Yoo, S. J. Kang, H. Yoo, H. Park, E. G. Kwon, and Y. I. Cho. 2015b. Multiplex quantitative real-time polymerase chain reaction assay for rapid detection of Mycobacterium avium subsp. paratuberculosis in fecal samples. J. Vet. Clin. 32(3):219-223.

Hanifian, S. 2014. Survival of Mycobacterium avium subsp. paratuberculosis in ultra-filtered white cheese. Lett. Appl. Microbiol. 58(5):466-471.

Hanifian, S. 2020. Behavior of Mycobacterium avium paratuberculosis in Lighvan cheese tracked by propidium monoazide qPCR and culture. LWT 133.

Hanifian, S. and S. Khani. 2016. Tracking of Mycobacterium Avium Paratuberculosis Load in Milk Production Chain: A Real-Time qPCR And Culture Assay. J. Food Saf. 36(1):136-141.

Hanifian, S., S. Khani, A. Barzegari, and J. Shayegh. 2013. Quantitative real-time PCR and culture examination of Mycobacterium avium subsp. paratuberculosis at farm level. Vet. Microbiol. 162(1):160-165.

Herthnek, D. and G. Bolske. 2006. New PCR systems to confirm real-time PCR detection of Mycobacterium avium subsp. paratuberculosis. BMC Microbiol. 6(87).

Hruška, K., M. Bartos, P. Kralik, and I. Pavlik. 2005. Mycobacterium avium subsp. paratuberculosis in powdered infant milk: Paratuberculosis in cattle - the public health problem to be solved. Vet. Med. 50(8):327-335.

Hruska, K., I. Slana, P. Kralik, and I. Pavlik. 2011. Mycobacterium avium subsp. paratuberculosis in powdered infant milk: F57 competitive real time PCR. Vet. Med. 56(5):226-230.

Husakova, M., P. Kralik, V. Babak, and I. Slana. 2020. Efficiency of DNA isolation methods based on silica columns and magnetic separation tested for the detection of mycobacterium avium subsp. Paratuberculosis in milk and faeces. Mater. 13(22):1-11.

Irenge, L. M., K. Walravens, M. Govaerts, J. Godfroid, V. Rosseels, K. Huygen, and J.-L. Gala. 2009a. Development and validation of a triplex real-time PCR for rapid detection and specific identification of M. avium sub sp. paratuberculosis in faecal samples. Vet. Microbiol. 136(1-2):166-172.

Irenge, L. M., K. Walravens, M. Govaerts, J. Godfroid, V. Rosseels, K. Huygen, and J. L. Gala. 2009b. Development and validation of a triplex real-time PCR for rapid detection and specific identification of M. avium sub sp. paratuberculosis in faecal samples. Vet. Microbiol. 136(1-2):166-172.

Jurado-Martos, F., F. Cardoso-Toset, C. Tarradas, A. Galan-Relano, J. M. Sanchez-Carvajal, I. Ruedas-Torres, E. Vera, F. Larenas-Munoz, L. Gomez-Gascon, I. M. Rodriguez-Gomez, L. Carrasco, J. Gomez-Laguna, B. H. Lorenzo, and I. Luque. 2023. Diagnostic performance of faecal and tissue multiplex qPCR IS900/F57 for the detection of Mycobacterium avium subspecies paratuberculosis in cattle. Res. Vet. Sci. 161:156-162.

Karthikeyan, A., L. Gunaseelan, K. Porteen, and B. S. M. Ronald. 2019. Bio-load of mycobacterium avium subspecies paratuberculosis in buffaloes. Buffa. Bul. 38(3):497-504.

Keller, S. M., R. Stephan, R. Kuenzler, M. Meylan, and M. M. Wittenbrink. 2014. Comparison of fecal culture and F57 real-time polymerase chain reaction for the detection of Mycobacterium avium subspecies paratuberculosis in Swiss cattle herds with a history of paratuberculosis. Acta Vet. Scand. 56(1):68.

Keshavarz, R., N. Mosavari, K. Tadayon, and M. Haghkhah. 2018. Effectiveness of an inactivated paratuberculosis vaccine in iranian sheep flocks using the mycobacterium avium subsp paratuberculosis 316F strain. Iran. J. Microbiol. 10(2):117-122.

Khol, J. L., V. Beran, P. Kralik, M. Trckova, I. Pavlik, and W. Baumgartner. 2010a. Grass silage contaminated with Mycobacterium avium subspecies paratuberculosis (MAP): A possible source of paratuberculosis infection in ruminants? Vet. Med. 55(5):225-232.

Khol, J. L., A. L. Braun, I. Slana, P. Kralik, and T. Wittek. 2017. Testing of milk replacers for Mycobacterium avium subsp. paratuberculosis by PCR and bacterial culture as a possible source for Johne's disease (paratuberculosis) in calves. Prev. Vet. Med. 144:53-56.

Khol, J. L., P. Kralik, I. Slana, V. Beran, C. Aurich, W. Baumgartner, and I. Pavlik. 2010b. Consecutive excretion of Mycobacterium avium subspecies paratuberculosis in Semen of a breeding bull compared to the distribution in feces, tissue and blood by IS900 and F57 quantitative real-time PCR and culture examinations. J. Vet. Med. Sci. 72(10):1283-1288.

Klanicova, B., I. Slana, P. Roubal, I. Pavlik, and P. Kralik. 2012. Mycobacterium avium subsp. paratuberculosis survival during fermentation of soured milk products detected by culture and quantitative real time PCR methods. Int. J. Food Microbiol. 157(2):150-155.

Kralik, P., V. Babak, and R. Dziedzinska. 2014. Repeated cycles of chemical and physical disinfection and their influence on Mycobacterium avium subsp. paratuberculosis viability measured by propidium monoazide F57 quantitative real time PCR. Vet. J. 201(3):359-364.

Kralik, P., V. Beran, and I. Pavlik. 2012. Enumeration of Mycobacterium avium subsp. paratuberculosis by quantitative real-time PCR, culture on solid media and optical densitometry. BMC Res. Notes 5.

Kralik, P., A. Nocker, and I. Pavlik. 2010. Mycobacterium avium subsp. paratuberculosis viability determination using F57 quantitative PCR in combination with propidium monoazide treatment. Int. J. Food Microbiol. 141(Suppl. 1):S80-S86.

Kralik, P., I. Slana, A. Kralova, V. Babak, R. H. Whitlock, and I. Pavlik. 2011. Development of a predictive model for detection of Mycobacterium avium subsp. paratuberculosis in faeces by quantitative real time PCR. Vet. Microbiol. 149(1-2):133-138.

Leite, F. L., K. D. Stokes, S. Robbe-Austerman, and J. R. Stabel. 2013. Comparison of fecal DNA extraction kits for the detection of Mycobacterium avium subsp. paratuberculosis by polymerase chain reaction. J. Vet. Diagn. Invest. 25(1):27-34.

Maio, E., T. Carta, A. Balseiro, I. A. Sevilla, A. Romano, J. A. Ortiz, M. Vieira-Pinto, J. M. Garrido, J. M. P. de la Lastra, and C. Gortazar. 2011. Paratuberculosis in European wild rabbits from the Iberian Peninsula. Res. Vet. Sci. 91(2):212-218.

Maldonado-Castro, E., A. L. Hernández-Reyes, B. Arellano-Reynoso, C. G. Gutiérrez, M. Alonso-Hearn, and G. Chávez-Gris. 2023. Clinical course and pathogenicity of a Mycobacterium avium subsp. paratuberculosis isolate from scimitar oryx (Oryx dammah) in experimentally infected rabbits. Span. J. Agric. Res. 21(4).

Meadus, W. J., C. O. Gill, P. Duff, M. Badoni, and L. Saucier. 2008. Prevalence on beef carcasses of Mycobacterium avium subsp. paratuberculosis DNA. Int. J. Food Microbiol. 124(3):291-294.

Möbius, P., H. Hotzel, A. Raßbach, and H. Köhler. 2008. Comparison of 13 single-round and nested PCR assays targeting IS900, ISMav2, f57 and locus 255 for detection of Mycobacterium avium subsp. paratuberculosis. Vet. Microbiol. 126(4):324-333.

Moghaddam, T. G., M. Haghkhah, and G. Mohammadi. 2023. Investigating the Prevalence of Mycobacterium avium Subspecies Paratuberculosis (MAP) in Industrial Dairy herds using Ziehl-Neelsen Staining, Culture, and PCR in Mashhad, Iran. Iran. J. Veterinary Sci. Technol. 15(2):11-18.

Mohan, A., P. Das, N. Kushwaha, K. Karthik, and A. K. Niranjan. 2013. Investigation on the status of Johne's disease based on agar gel immunodiffusion, ziehl-neelsen staining and nested PCR approach in two cattle farm. Vet. World 6(10):778-784.

Muñoz, M. C., G. M. Basulto, T. R. Evangelista, F. M. Navarro, V. G. Vizcarra, and G. L. Valencia. 2013. Molecular characterization of Mycobacterium avium subspecies paratuberculosis from sheep and cattle in Mexicali, Baja California, Mexico. Rev. Mex. Cienc. Pecuarias 4(4):489-500.

Nouri, F., A. Shahrjerdi, H. A. Zarnegarpour, and N. Mosavari. 2024. Diagnostic advancements: Isolating Mycobacterium avium ssp. paratuberculosis and unveiling its molecular identity with nested-PCR. Cell. Mol. Biol. 70(2):18-23.

Özpinar, H., İ. H. Tekiner, O. Karaman, and Y. Kurt. 2015. Investigation of Mycobacterium avium subsp. Paratuberculosis (MAP) in fecal and bulk milk samples from dairy farms in thrace region of Turkey. Kafkas Universitesi Veteriner Fakultesi Dergisi 21(2):247-252.

Pagliasso, G., A. Di Blasio, N. Vitale, A. Romano, L. Decastelli, A. Quasso, M. Ricchi, A. Dondo, P. Pastorino, M. S. Gennero, and S. Bergagna. 2021. Goat Paratuberculosis: Experimental Model for the Evaluation of Mycobacterium Persistence in Raw Milk Cheese. Microorganisms 9(10):2032.

Park, H., M. Shin, H. Park, Y. Cho, and H. Yoo. 2016. PCR-based detection of Mycobacterium avium subsp. paratuberculosis infection in cattle in South Korea using fecal samples. J. Vet. Med. Sci. 78(9):1537-1540.

Pisanu, S., T. Cubeddu, C. Cacciotto, Y. Pilicchi, D. Pagnozzi, S. Uzzau, S. Rocca, and M. F. Addis. 2018. Characterization of paucibacillary ileal lesions in sheep with subclinical active infection by Mycobacterium avium subsp. Paratuberculosis. Vet. Res. 49(1).

Pithua, P., S. M. Godden, S. J. Wells, and J. R. Stabel. 2011a. Evaluation of the risk of paratuberculosis in adult cows fed Mycobacterium avium subsp paratuberculosis DNA-positive or -negative colostrum as calves. Am. J. Vet. Res. 72(11):1456-1464.

Pithua, P., S. J. Wells, and S. M. Godden. 2011b. Evaluation of the association between fecal excretion of Mycobacterium avium subsp paratuberculosis and detection in colostrum and on teat skin surfaces of dairy cows. J. Am. Vet. Med. Assoc. 238(1):94-100.

Pithua, P., S. J. Wells, S. M. Godden, S. Sreevatsan, and J. R. Stabel. 2010. Experimental validation of a nested polymerase chain reaction targeting the genetic element ISMAP02 for detection of Mycobacterium avium subspecies paratuberculosis in bovine colostrum. J. Vet. Diagn. Invest. 22(2):253-256.

Pribylova, R., L. Kubickova, V. Babak, I. Pavlik, and P. Kralik. 2012. Effect of short- and long-term antibiotic exposure on the viability of Mycobacterium avium subsp. paratuberculosis as measured by propidium monoazide F57 real time quantitative PCR and culture. Vet. J. 194(3):354-360.

Pribylova, R., I. Slana, P. Kralik, A. Kralova, V. Babak, and I. Pavlik. 2011. Correlation of Mycobacterium avium subsp. paratuberculosis counts in gastrointestinal tract, muscles of the diaphragm and the masseter of dairy cattle and potential risk for consumers. Int. J. Food Microbiol. 151(3):314-318.

Rad, A. H. F., M. R. Bassami, and A. Mirzapoor. 2010. Prevalence of MAP in a large dairy herd and its effect on reproductive and production indices. Journal of Animal and Veterinary Advances 9(1):149-154.

Rani, M., D. Narang, D. Kumar, M. Chandra, S. T. Singh, and G. Filia. 2018. ISMap02 element targeted nested polymerase chain in the detection of Mycobacterium avium subsp. paratuberculosis in fecal samples of cattle and buffaloes. Vet. World 11(3):397-401.

Rani, P. S., S. N. Doddam, S. Agrawal, S. E. Hasnain, L. A. Sechi, A. Kumar, and N. Ahmed. 2014. Mycobacterium avium subsp. paratuberculosis is not discerned in diabetes mellitus patients in Hyderabad, India. Int. J. Med. Microbiol. 304(5-6):620-625.

Rasper-Hossinger, M., M. Biggel, R. Stephan, F. Seehusen, and S. Scherrer. 2023. Strain diversity in Mycobacterium avium subsp. paratuberculosis-positive bovine fecal samples collected in Switzerland. Front. Vet. Sci. 10.

Rhim, H., Y. I. Cho, H. J. Jang, K. J. Na, and J. I. Han. 2018. High prevalence of mycobacterium avium subsp. Paratuberculosis in wild ducks in the middle area of South Korea. J. Vet. Clin. 35(1):7-9.

Ricchi, M., R. Savi, L. Bolzoni, S. Pongolini, I. R. Grant, C. De Cicco, G. Cerutti, G. Cammi, C. A. Garbarino, and N. Arrigoni. 2016. Estimation of Mycobacterium avium subsp. paratuberculosis load in raw bulk tank milk in Emilia-Romagna Region (Italy) by qPCR. MicrobiologyOpen 5(4):551-559.

Ritter, C., R. Wolf, C. L. Adams, D. F. Kelton, C. Pickel, S. Mason, K. Orsel, J. De Buck, and H. W. Barkema. 2016. Short communication: Herd-level preitalence of Mycobacterium avium ssp paratuberculosis is not associated with participation in a voluntary Alberta Johne's disease control program. J. Dairy Sci. 99(3):2157-2160.

Rocca, S., T. Cubeddu, A. M. Nieddu, S. Pirino, S. Appino, E. Antuofermo, F. Tanda, R. Verin, L. A. Sechi, E. Taccini, and A. Leoni. 2010. Detection of Mycobacterium avium spp. paratuberculosis (Map) in samples of sheep paratuberculosis (Johne's disease or JD) and human Crohn's disease (CD) using liquid phase RT-PCR, in situ RT-PCR and immunohistochemistry. Small Ruminant Res. 88(2-3):126-134.

Rojas-Ponce, G., D. Sauvageau, R. Zemp, H. W. Barkema, and S. Evoy. 2022. Use of uncoated magnetic beads to capture Mycobacterium smegmatis and Mycobacterium avium paratuberculosis prior detection by mycobacteriophage D29 and real-time-PCR. J. Microbiol. Methods 197.

Russo, S., C. Cortimiglia, A. Filippi, G. Palladini, C. Garbarino, E. Massella, and M. Ricchi. 2023. Validation of digital PCR assay for the quantification of Mycobacterium avium subsp. paratuberculosis in bovine faeces according to the ISO 20395:2019. J. Microbiol. Methods 213.

Sadeghi, N., A. Jamshidi, and M. Seyyedin. 2020. Detection of mycobacterium avium sub sp. paratuberculosis in pasteurized milk samples in northeast of Iran by culture, direct nested PCR and PCR methods. Iran. J. Chem. Chem. Eng. 39(6):251-258.

Salgado, M., M. T. Collins, F. Salazar, J. Kruze, G. Bölske, R. Söderlund, R. Juste, I. A. Sevilla, F. Biet, F. Troncoso, and M. Alfaro. 2011. Fate of Mycobacterium avium subsp. paratuberculosis after application of contaminated dairy cattle manure to agricultural soils. Appl. Environ. Microbiol. 77(6):2122-2129.

Salgado, M., D. Herthnek, G. Bölske, S. Leiva, and J. Kruze. 2009. First isolation of Mycobacterium avium subsp. paratuberculosis from wild guanacos (Lama guanicoe) on tierra del fuego Island. J. Wildl. Dis. 45(2):295-301.

Sange, M. D., A. Becker, A. A. Hassan, M. Buelte, M. Ganter, U. Siebert, and A. Abdulmawjood. 2019a. Development and validation of a loop-mediated isothermal amplification assay-a rapid and sensitive detection tool for Mycobacterium avium subsp. paratuberculosis in small ruminants. J. Appl. Microbiol. 127(1):47-58.

Sange, M. D., A. Becker, A. A. Hassan, M. Bülte, M. Ganter, U. Siebert, and A. Abdulmawjood. 2019b. Development and validation of a loop-mediated isothermal amplification assay—a rapid and sensitive detection tool for Mycobacterium avium subsp. paratuberculosis in small ruminants. J. Appl. Microbiol. 127(1):47-58.

Schönenbrücher, H., A. Abdulmawjood, K. Failing, and M. Bülte. 2008. New triplex real-time PCR assay for detection of Mycobacterium avium subsp. paratuberculosis in bovine feces. Appl. Environ. Microbiol. 74(9):2751-2758.

Selim, A. M. and W. Gaede. 2015. Comparative evaluation of PCR assay for direct detection of mycobacterium avium subsp. paratuberculosis in ruminant. Asian J. Anim. Vet. Adv. 10(11):761-771.

Selvam, A., K. Vijayarani, and K. Kumanan. 2009. Detection of mycobacterium avium subsp paratuberculosis in milk of asymptomatic sheep by polymerase chain reaction. Indian J. Anim. Sci. 79(8):784-785.

Seva, J., J. M. Sanes, G. Ramis, A. Mas, J. J. Quereda, B. Villarreal-Ramos, D. Villar, and F. J. Pallares. 2014. Evaluation of the single cervical skin test and interferon gamma responses to detect Mycobacterium bovis infected cattle in a herd co-infected with Mycobacterium avium subsp paratuberculosis. Vet. Microbiol. 171(1-2):139-146.

Sevilla, I. A., J. M. Garrido, E. Molina, M. V. Geijo, N. Elguezabal, P. Vazquez, and R. A. Juste. 2014. Development and evaluation of a novel multicopy-element-targeting triplex PCR for detection of Mycobacterium avium subsp. paratuberculosis in feces. Appl. Environ. Microbiol. 80(12):3757-3768.

Sidoti, F., G. Banche, S. Astegiano, V. Allizond, A. M. Cuffini, and M. Bergallo. 2011. Validation and standardization of IS900 and F57 real-time quantitative PCR assays for the specific detection and quantification of Mycobacterium avium subsp. paratuberculosis. Can. J. Microbiol. 57(5):347-354.

Singh, U., D. Arutyunov, U. Basu, H. D. Santos Seckler, C. M. Szymanski, and S. Evoy. 2014. Mycobacteriophage lysin-mediated capture of cells for the PCR detection of Mycobacterium avium subspecies paratuberculosis. Anal. Methods 6(15):5682-5689.

Slana, I., P. Kralik, A. Kralova, and I. Pavlik. 2008. On-farm spread of Mycobacterium avium subsp. paratuberculosis in raw milk studied by IS900 and F57 competitive real time quantitative PCR and culture examination. Int. J. Food Microbiol. 128(2):250-257.

Slana, I., M. Liapi, M. Moravkova, A. Kralova, and I. Pavlik. 2009. Mycobacterium avium subsp. paratuberculosis in cow bulk tank milk in Cyprus detected by culture and quantitative IS900 and F57 real-time PCR. Prev. Vet. Med. 89(3-4):223-226.

Slana, I., R. Pribylova, A. Kralova, and I. Pavlik. 2011. Persistence of Mycobacterium avium subsp. paratuberculosis at a farm-scale biogas plant supplied with manure from paratuberculosis-affected dairy cattle. Appl. Environ. Microbiol. 77(9):3115-3119.

Sobrino, R., O. Aurtenetxe, T. Carta, L. Mamian, X. Gerrikagoitia, A. Balseiro, A. Oleaga, I. A. Sevilla, M. Barral, J. M. Garrido, and C. Gortazar. 2011. Lack of evidence of paratuberculosis in wild canids from southwestern Europe. Eur. J. Wildl. Res. 57(3):683-688.

Stabel, J. R. and J. P. Bannantine. 2005. Development of a nested PCR method targeting a unique multicopy element, ISMap02, for detection of Mycobacterium avium subsp. paratuberculosis in fecal samples. J. Clin. Microbiol. 43(9):4744-4750.

Stephan, R., S. Schumacher, T. Tasara, and I. R. Grant. 2007. Prevalence of mycobacterium avium subspecies paratuberculosis in swiss raw milk cheeses collected at the retail level. J. Dairy Sci. 90(8):3590-3595.

Stief, B., P. Möbius, H. Türk, U. Hörügel, C. Arnold, and D. Pöhle. 2012. Paratuberculosis in a miniature donkey (Equus asinus f. asinus). Berl. Munch. Tierarztl. Wochenschr. 125(1-2):38-44.

Tasara, T., L. E. Hoelzle, and R. Stephan. 2005. Development and evaluation of a Mycobacterium avium subspecies paratuberculosis (MAP) specific multiplex PCR assay. Int. J. Food Microbiol. 104(3):279-287.

Tasara, T. and R. Stephan. 2005. Development of an F57 sequence-based real-time PCR assay for detection of Mycobacterium avium subsp. paratuberculosis in milk. Appl. Environ. Microbiol. 71(10):5957-5968.

Timms, V. J., M. M. Gehringer, H. M. Mitchell, G. Daskalopoulos, and B. A. Neilan. 2011. How accurately can we detect Mycobacterium avium subsp paratuberculosis infection? J. Microbiol. Methods 85(1):1-8.

Timms, V. J., H. M. Mitchell, and B. A. Neilan. 2015. Optimisation of DNA extraction and validation of PCR assays to detect Mycobacterium avium subsp. paratuberculosis. J. Microbiol. Methods 112:99-103.

Tripathi, B. N. and K. Stevenson. 2010. Molecular characterisation of Indian strains of Mycobacterium avium subspecies paratuberculosis by pulsed-field gel electrophoresis. J. Appl. Anim. Res. 37(2):247-251.

Vansnick, E., P. De Rijk, F. Vercammen, D. Geysen, L. Rigouts, and F. Portaels. 2004. Newly developed primers for the detection of Mycobacterium avium subspecies paratuberculosis. Vet. Microbiol. 100(3-4):197-204.

Vijayarani, K. and K. Kumaman. 2008. A rapid method for PCR amplification of specific genes of Mycobacterium avium sub sp. paratuberculosis. Indian J. Anim. Sci. 78(10):1049-1051.

Wolf, R., K. Orsel, J. De Buck, and H. W. Barkema. 2015. Calves shedding Mycobacterium avium subspecies paratuberculosis are common on infected dairy farms Modeling Johne's disease: From the inside out Dr Ad Koets and Prof Yrjo Grohn. Vet. Res. 46(1).

Youssef, D. G. S., F. A. Sallam, S. F. Darwish, and A. S. Amin. 2014. Evaluation of conventional and real-time PCR assays for molecular diagnosis of Johne's disease in dairy cattle. International Journal of Current Microbiology and Applied Sciences 3(10):969-981.
