# Supplementary material for: Systematic evaluation and optimization of TaqMan qPCR assays targeting F57, ISMAP02, and IS900 for multiplex detection of Mycobacterium avium subsp. paratuberculosis
Source: J Clin Microbiol. 2025 Dec 29;64(2):e00872-25. doi: 10.1128/jcm.00872-25 (PMC12892987; doi:10.1128/jcm.00872-25)
Supplement: Table S5 — Statistical analysis of the qPCR (Cq) results obtained with the moderate MAP shedders using the 18 multiplex-qPCR assays. [file jcm.00872-25-s0007.docx]

**Supplemental Table S5.** Results of the 18 qPCR multiplex assays for the detection of MAP in fecal samples from cows excreting moderate level of MAP (F11 to F16)^1^.

| Design  (IS*900*-ISMAP*02*-F*57*) | IS*900* target | | ISMAP*02* target^2^ | | F*57* target | |
| --- | --- | --- | --- | --- | --- | --- |
| Herthnek-Irenge-Herthnek | 26.56 ± 0.59^efi^ | (25.39, 27.73) | 27.65 ± 0.69^a^ | (26.28, 29.03) | 29.82 ± 0.67^ab^ | (28.48, 31.15) |
| Herthnek-Irenge-Irenge | 26.56 ± 0.63^bcdefg^ | (25.31, 27.82) | 27.82 ± 0.66^a^ | (26.51, 29.13) | 29.49 ± 0.61^ab^ | (28.28, 30.70) |
| Herthnek-Irenge-Ricchi | 26.51 ± 0.56^efghi^ | (25.40, 27.63) | 27.80 ± 0.67^a^ | (26.47, 29.12) | 29.65 ± 0.72^ab^ | (28.22, 31.08) |
| Herthnek-Sevilla-Herthnek | 26.87 ± 0.56^abcd^ | (25.75, 27.98) | 27.95 ± 0.63^a^ | (26.70, 29.21) | 29.87 ± 0.67^ab^ | (28.55, 31.20) |
| Herthnek-Sevilla-Irenge | 27.03 ± 0.61^a^ | (25.81, 28.25) | 27.94 ± 0.66^a^ | (26.64, 29.25) | 29.67 ± 0.60^ab^ | (28.49, 30.86) |
| Herthnek-Sevilla-Ricchi | 27.03 ± 0.61^a^ | (25.81, 28.25) | 27.92 ± 0.68^a^ | (26.57, 29.27) | 29.75 ± 0.63^ab^ | (28.50, 31.00) |
| Kim-Irenge-Herthnek | 26.22 ± 0.73^dij^ | (24.78, 27.67) | 28.22 ± 0.87^a^ | (26.49, 29.96) | 29.99 ± 0.74^ab^ | (28.52, 31.46) |
| Kim-Irenge-Irenge | 26.35 ± 0.71^cdij^ | (24.94, 27.77) | 28.41 ± 0.91^a^ | (26.60, 30.22) | 29.71 ± 0.69^ab^ | (28.35, 31.08) |
| Kim-Irenge-Ricchi | 26.37 ± 0.72^cdij^ | (24.94, 27.79) | 28.23 ± 0.82^a^ | (26.61, 29.85) | 31.02 ± 1.09^a^ | (28.85, 33.19) |
| Kim-Sevilla-Herthnek | 26.27 ± 0.60^ghj^ | (25.08, 27.46) | 27.96 ± 0.66^a^ | (26.65, 29.26) | 29.82 ± 0.65^ab^ | (28.53, 31.11) |
| Kim-Sevilla-Irenge | 26.34 ± 0.64^fghi^ | (25.08, 27.61) | 28.02 ± 0.69^a^ | (26.64, 29.39) | 29.74 ± 0.64^ab^ | (28.46, 31.02) |
| Kim-Sevilla-Ricchi | 26.25 ± 0.61^hj^ | (25.05, 27.46) | 27.90 ± 0.65^a^ | (26.60, 29.20) | 29.66 ± 0.64^ab^ | (28.38, 30.94) |
| Slana-Irenge-Herthnek | 26.84 ± 0.72^abe^ | (25.40, 28.28) | 27.99 ± 0.65^a^ | (26.69, 29.28) | 29.77 ± 0.62^ab^ | (28.54, 31.00) |
| Slana-Irenge-Irenge | 26.75 ± 0.74^abefgh^ | (25.28, 28.21) | 27.92 ± 0.63^a^ | (26.67, 29.17) | 29.58 ± 0.63^ab^ | (28.32, 30.83) |
| Slana-Irenge-Ricchi | 26.67 ± 0.68^abcefg^ | (25.31, 28.03) | 27.95 ± 0.62^a^ | (26.72, 29.18) | 29.48 ± 0.76^b^ | (27.97, 30.99) |
| Slana-Sevilla-Herthnek | 26.55 ± 0.64^bcdefgh^ | (25.28, 27.82) | 28.04 ± 0.58^a^ | (26.88, 29.20) | 29.79 ± 0.57^ab^ | (28.66, 30.91) |
| Slana-Sevilla-Irenge | 26.79 ± 0.65^abe^ | (25.50, 28.07) | 28.10 ± 0.56^a^ | (26.98, 29.21) | 29.63 ± 0.54^ab^ | (28.56, 30.70) |
| Slana-Sevilla-Ricchi | 26.80 ± 0.68^abe^ | (25.45, 28.15) | 28.15 ± 0.58^a^ | (27.00, 29.30) | 29.76 ± 0.64^ab^ | (28.50, 31.02) |

^1^qPCR cycle of quantification values obtained for each target and multiplex assay are presented as adjusted means ± SE, and 95% CI are indicated in parentheses; mean differences between designs were evaluated using multiple comparisons with a Tukey adjustment; ^a-j^values in the same column with different superscripts differ (*P* < 0.05).

^2^The mean Cq value (± SD) using the VetMAX-Gold MAP Detection Kit was 28.41 (± 1.56).
