# Supplementary material for: Systematic evaluation and optimization of TaqMan qPCR assays targeting F57, ISMAP02, and IS900 for multiplex detection of Mycobacterium avium subsp. paratuberculosis
Source: J Clin Microbiol. 2025 Dec 29;64(2):e00872-25. doi: 10.1128/jcm.00872-25 (PMC12892987; doi:10.1128/jcm.00872-25)
Supplement: Figure S1 — Quantification of MAP excreted in feces (Cq value) and anti-MAP antibody levels (serum ELISA, %S/P) according to the age of animals of low (A), moderate (B) and high (C) shedders and MAP level (Cq) from the environnemental samples (D). [file jcm.00872-25-s0001.pdf]

Index

A) Low-MAP level excreting cows (F05-F10) ....Page 1

B) Moderate-MAP level excreting cows (F11-F16) ....Page 2

C) High-MAP level excreting cows (F17-F25) ....Page 3

D) Environmental MAP level in A-D herds (E01-E08) ....Pages 5-6

A) Low-MAP level excreting cows

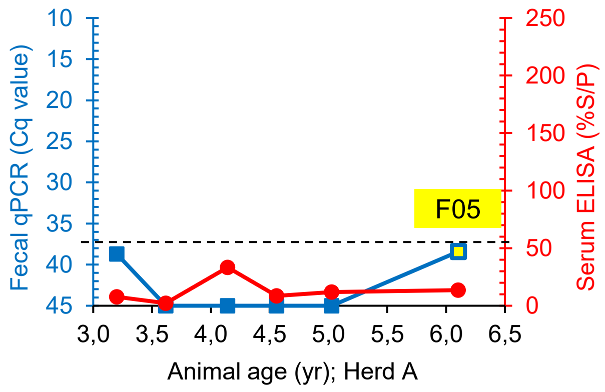

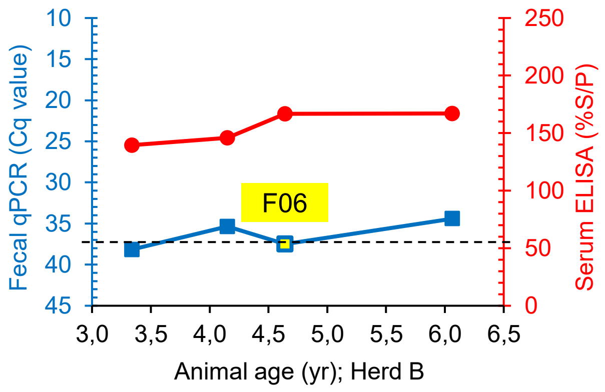

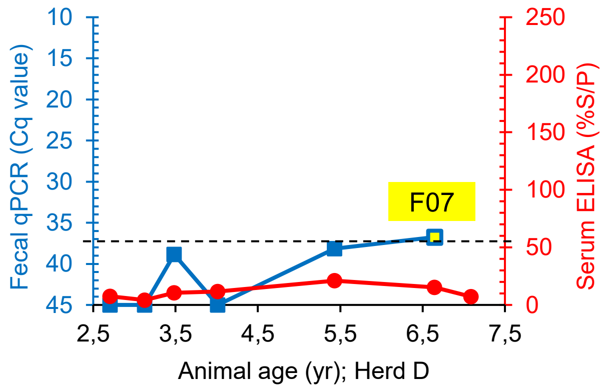

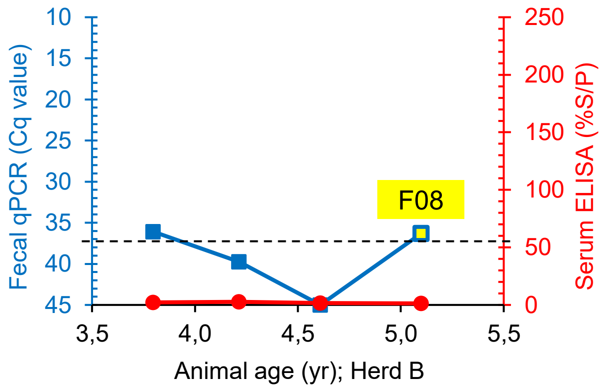

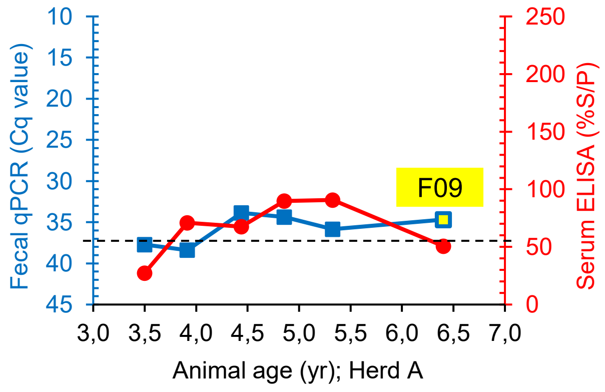

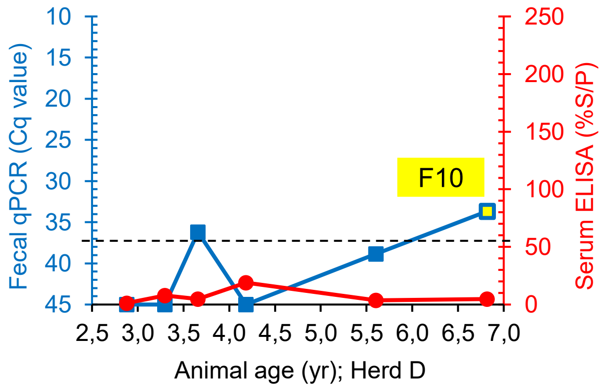

B) Moderate-MAP level excreting cows

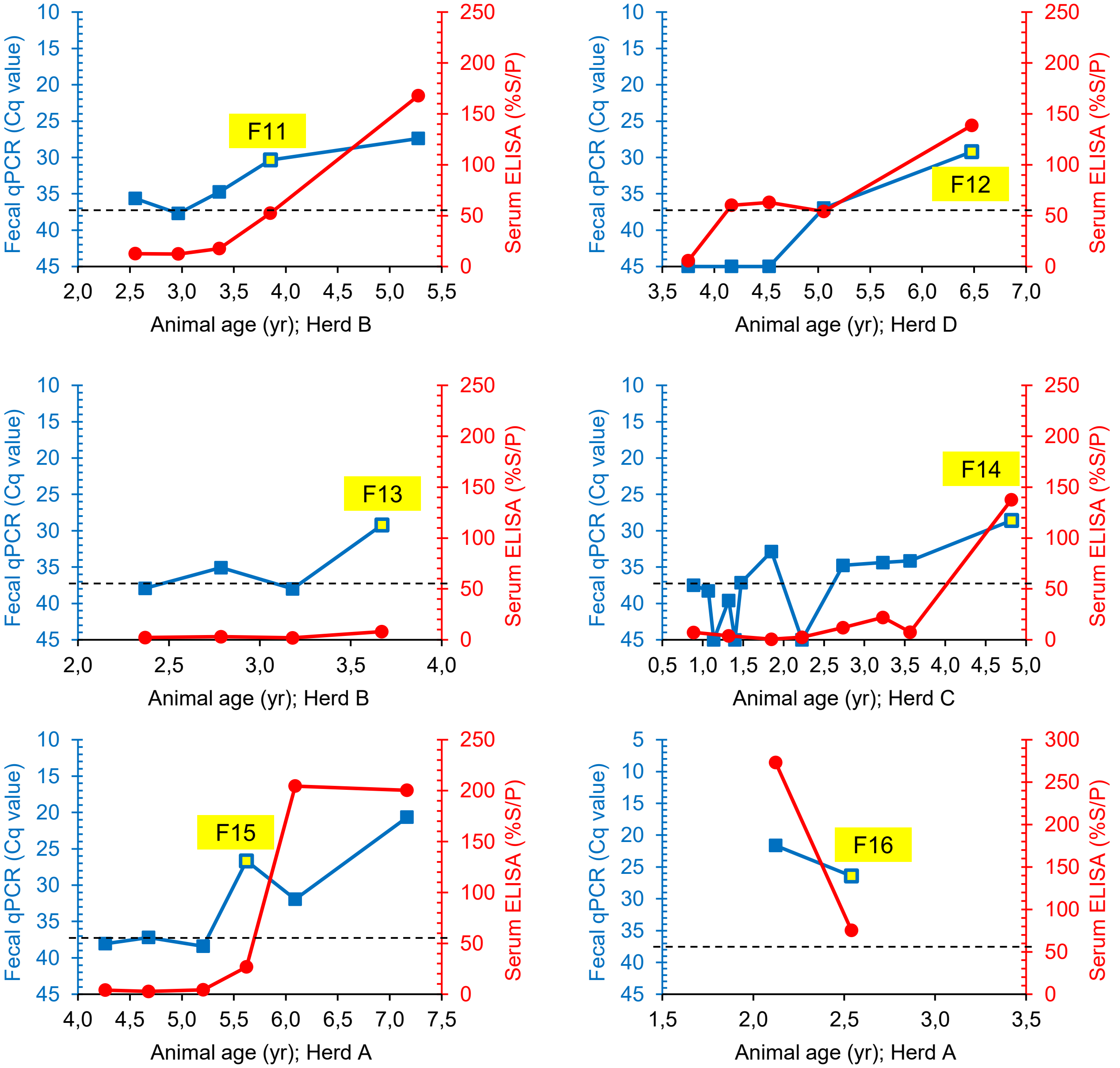

C) High-MAP level excreting cows

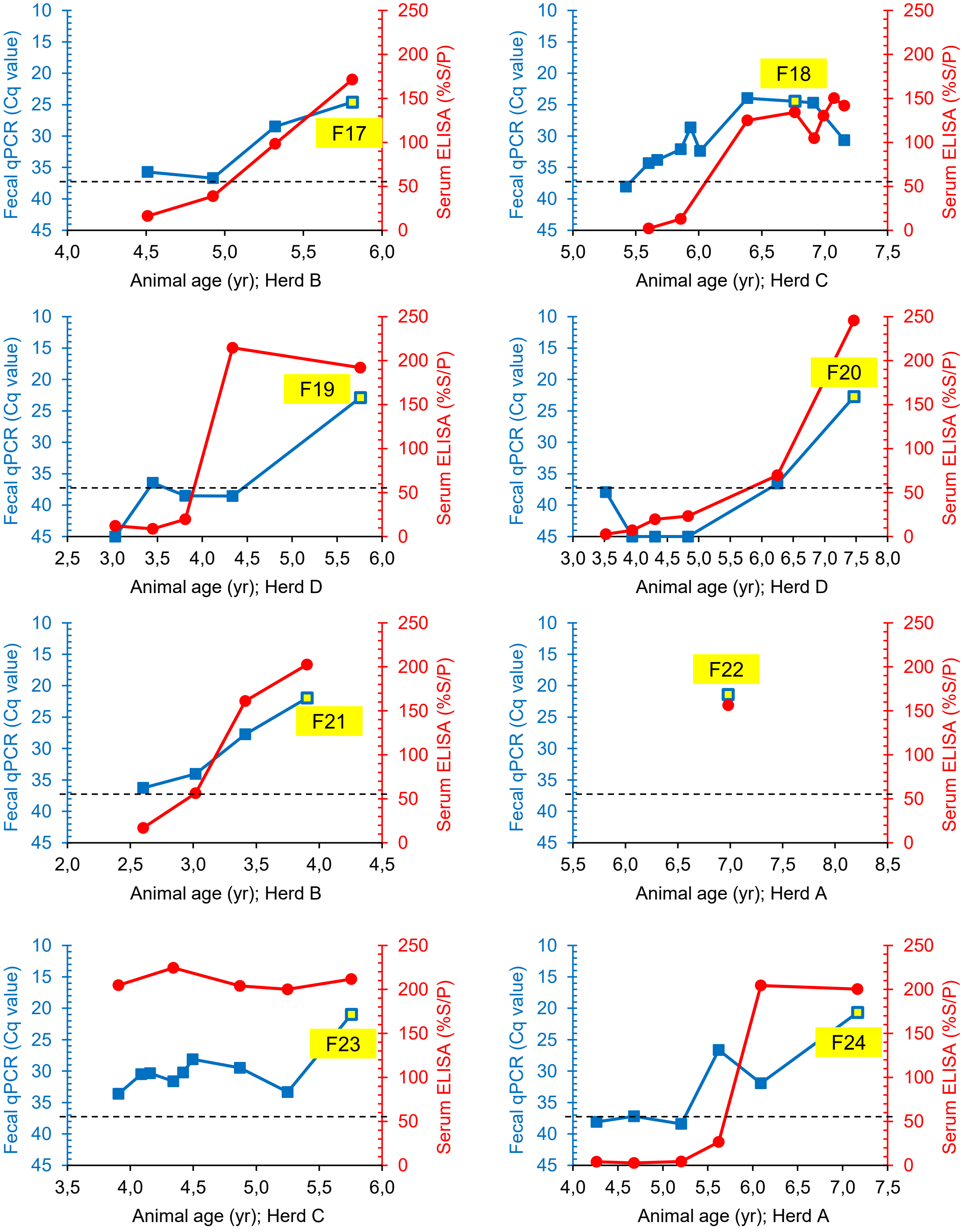

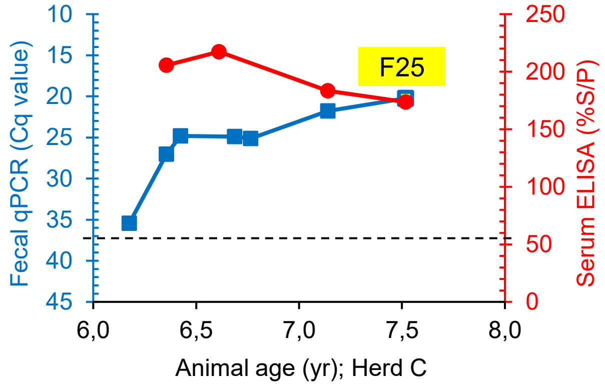

Legend of Supplemental Fig. S1 A-C. Quantification of MAP levels excreted in feces (Cq value) and anti-MAP antibody levels (serum ELISA, %S/P) according to the age of animals included in the current study (from four herds, labeled A to D). The red line with circular markers represents the serum MAP ELISA %S/P values at each sampling time point, while the blue line with square markers indicates the Cq values obtained from fecal qPCR analysis using the USDA-licensed VetMAX-Gold MAP detection kit. For each sample, the reported values represent the mean of two independent serum ELISA tests and two qPCR assays performed on DNA extracted from two separate fecal aliquots. Fecal samples labeled F05 to F25, which were used for the comparative evaluation of 18 multiplex qPCR assays, are highlighted in yellow. Dashed lines indicate the positivity thresholds recommended by the respective commercial kits: ≥55% S/P (right Y-axis) for ELISA and Cq <37 (left Y-axis) for qPCR.

D) Environmental MAP-level in four herds

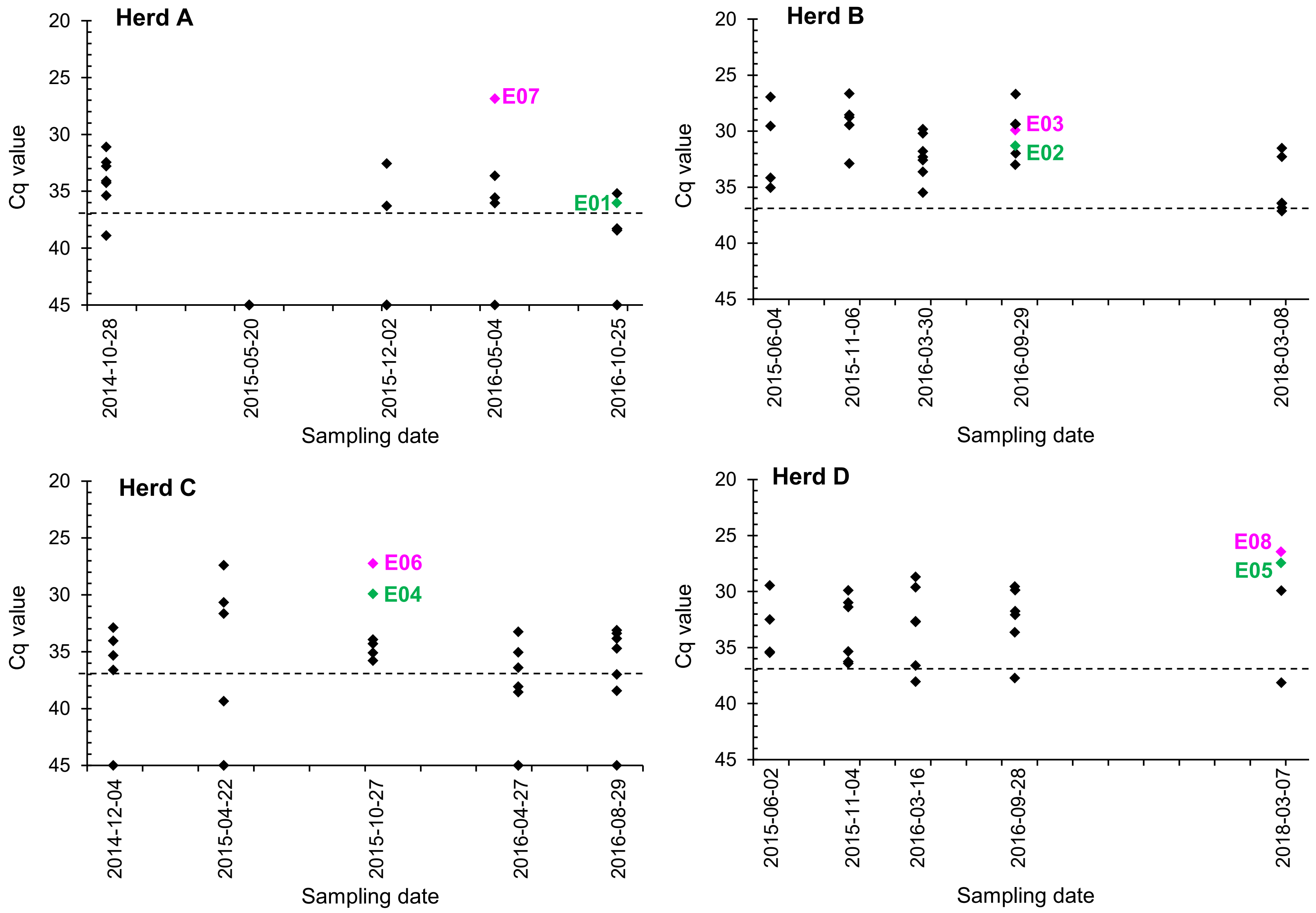

Legend of Supplemental Fig. S1 for environmental MAP-level. Quantification of MAP levels in environmental samples collected from four herds (A to D) at various sampling time points. Each dot represents the quantification cycle (Cq) value obtained from qPCR

analysis using the USDA-licensed VetMAX-Gold MAP detection kit. For each sample, the reported Cq value corresponds to the average of two independent qPCR assays performed on DNA extracted from two separate aliquots of the environmental sample. Environmental samples labeled E01 to E08, which were used in the comparative evaluation of 18 multiplex qPCR assays, are shown for each herd. The dashed line indicates the Cq threshold (<37) recommended by the commercial kit to classify a sample as MAP-positive.
